# Socioeconomic Differences and Lung Cancer Survival—Systematic Review and Meta-Analysis

**DOI:** 10.3389/fonc.2018.00536

**Published:** 2018-11-27

**Authors:** Isabelle Finke, Gundula Behrens, Linda Weisser, Hermann Brenner, Lina Jansen

**Affiliations:** ^1^Division of Clinical Epidemiology and Aging Research, German Cancer Research Center (DKFZ), Heidelberg, Germany; ^2^Medical Faculty Heidelberg, University of Heidelberg, Heidelberg, Germany; ^3^Division of Preventive Oncology, German Cancer Research Center (DKFZ) and National Center for Tumor Diseases (NCT), Heidelberg, Germany; ^4^German Cancer Consortium (DKTK), German Cancer Research Center (DKFZ), Heidelberg, Germany

**Keywords:** socioeconomic status, lung cancer, cancer survival, area-based, education, income, occupation, index

## Abstract

**Background:** The impact of socioeconomic differences on cancer survival has been investigated for several cancer types showing lower cancer survival in patients from lower socioeconomic groups. However, little is known about the relation between the strength of association and the level of adjustment and level of aggregation of the socioeconomic status measure. Here, we conduct the first systematic review and meta-analysis on the association of individual and area-based measures of socioeconomic status with lung cancer survival.

**Methods:** In accordance with PRISMA guidelines, we searched for studies on socioeconomic differences in lung cancer survival in four electronic databases. A study was included if it reported a measure of survival in relation to education, income, occupation, or composite measures (indices). If possible, meta-analyses were conducted for studies reporting on individual and area-based socioeconomic measures.

**Results:** We included 94 studies in the review, of which 23 measured socioeconomic status on an individual level and 71 on an area-based level. Seventeen studies were eligible to be included in the meta-analyses. The meta-analyses revealed a poorer prognosis for patients with low individual income (pooled hazard ratio: 1.13, 95 % confidence interval: 1.08–1.19, reference: high income), but not for individual education. Group comparisons for hazard ratios of area-based studies indicated a poorer prognosis for lower socioeconomic groups, irrespective of the socioeconomic measure. In most studies, reported 1-, 3-, and 5-year survival rates across socioeconomic status groups showed decreasing rates with decreasing socioeconomic status for both individual and area-based measures. We cannot confirm a consistent relationship between level of aggregation and effect size, however, comparability across studies was hampered by heterogeneous reporting of socioeconomic status and survival measures. Only eight studies considered smoking status in the analysis.

**Conclusions:** Our findings suggest a weak positive association between individual income and lung cancer survival. Studies reporting on socioeconomic differences in lung cancer survival should consider including smoking status of the patients in their analysis and to stratify by relevant prognostic factors to further explore the reasons for socioeconomic differences. A common definition for socioeconomic status measures is desirable to further enhance comparisons between nations and across different levels of aggregation.

## Introduction

### Rationale

With 34.2 and 13.6 lung cancer cases per 100,000 per year for men and women around the world, respectively, lung cancer has the highest incidence rate for men and the fourth highest incidence rate for women (1). Regarding mortality, lung cancer has the highest rate in men and the second highest rate in women worldwide (1). Five-year survival rates vary considerably across countries with estimates between 10 and 20 % (2). These differences were even observed when comparing countries of similar structures in health care and access to care, such as the Scandinavian countries Sweden, Norway, and Denmark (3). Variations in the distribution of prognostic factors, such as stage, are likely to at least partly explain these differences (3). Numerous other prognostic factors have been investigated which include tumor-related factors like lung cancer subtype but also patient-related factors, such as age, gender, and comorbidities as well as smoking status and cancer treatment (4). For example, a later stage at diagnosis, male gender and current smoking at diagnosis have been shown to predict poor prognosis in lung cancer patients (5–7).

Another well-established prognostic factor for various cancer sites is socioeconomic status (SES) (8). Socioeconomic differences in cancer survival have been investigated and summarized by systematic reviews for different cancer types, such as breast (9, 10), colorectal (11), and prostate cancer (12). A recent meta-analysis reported lower breast cancer survival for women with lower SES even after adjustment for tumor characteristics, treatment, comorbidity or lifestyle-factors (10). Manser and Bauerfeind (11) reported in their systematic review significantly lower 1- and 5-year colorectal cancer survival rates for the lowest socioeconomic group compared to the highest socioeconomic group. Generally for all cancer types, neither stage at diagnosis nor treatment factors could entirely explain the association between SES and cancer survival (13).

For lung cancer, socioeconomic differences in incidence, mortality and treatment patterns have been summarized in systematic reviews, meta-, and pooled-analyses. A meta-analysis reported an increased risk in lung cancer incidence for lower socioeconomic groups with similar effect estimates in studies adjusting and not adjusting for smoking status (14). These results were confirmed by a recent international pooled analysis of case-control studies including detailed information on occupations and smoking behavior of around 17,000 cases and 20,000 controls (15). An analysis including 16 European populations reported higher lung cancer mortality rates in groups with lowest educational attainment (16). Another systematic review focused on lung cancer and showed higher lung cancer incidence and mortality in socioeconomically deprived areas (17). Tumor stage was not found to be associated with deprivation. However, stage might still confound associations between deprivation and lung cancer survival (18). Regarding treatment of lung cancer (19), the probability of receiving any type of treatment, surgery, and chemotherapy was lower in more deprived groups compared to the least deprived groups (19). To date, a systematic summary of findings regarding socioeconomic differences and lung cancer survival outcomes has not yet been provided.

SES can be measured for each patient individually (for example via questionnaire) or by using an ecological approach, meaning that a particular SES level is assigned to the residential area of each study participant (20). The latter can be called area-based studies which are often conducted if no individual SES data are available or if the effect of the area-based SES on health-related outcomes of a study participant is investigated (20). In such area-based studies, the aggregation level might be important. For patients with a diagnosis of breast cancer resident in England, it has been shown that the difference in crude survival between the most and the least deprived groups was 25 % smaller when using larger geographic units compared to smaller units (21). This dilution effect is caused by an increase in social heterogeneity the larger the area-level is (21). Another example from Australia reported stronger associations between socioeconomic disadvantage and the risk of cancer death and a more consistent socioeconomic gradient for the smaller geographical unit (22). However, this effect has not been investigated for lung cancer and has often been neglected in systematic reviews and meta-analyses. Furthermore, detailed meta-analyses regarding prognostic factors and their potential confounding in the association between socioeconomic measures and lung cancer survival have not yet been provided.

### Objectives

In our systematic review and meta-analysis, we provide a comprehensive summary on the current literature on socioeconomic differences in lung cancer survival with a focus on the impact of aggregation and adjustment level. The results of our review may inform health care planners about disparities in the prognosis of lung cancer patients and might help to more precisely identify socioeconomic deprived groups to counteract these differences.

### Research question

We investigated three research questions:

1. What is the current state of research on socioeconomic differences in lung cancer survival with regard to studies measuring individual or aggregated socioeconomic status?
2. To what extend does a potential gradient in lung cancer survival by socioeconomic status vary by level of exposure definition (e.g., individual level, community level)?
3. Which prognostic factors have an impact on differences in socioeconomic status, particularly regarding the association with lung cancer survival?

## Methods

### Systematic review protocol

The systematic review was conducted and reported in accordance with the Preferred Reporting Items for Systematic Reviews and Meta-Analysis (PRISMA) guidelines (23) and the extended version for equity-focused systematic reviews PRISMA-E 2012 (24). This review is registered in the international prospective register for systematic reviews PROSPERO (www.crd.york.ac.uk/PROSPERO, registration number: CRD42017072607).

### Literature search

The main information sources for the literature search were four databases: Medline/PubMed (1966 to December 6, 2017), Web of Science (Science Citation Index Expanded, Social Science Citation Index, 1945 to December 7, 2017), The Cochrane Library (1992 to December 6, 2017), and GESIS Sowiport (1910 to December 8, 2017). The online portal Sowiport is organized by the GESIS Leibniz Institute for the Social Sciences (25) and included several social science related databases until its termination in December 2017. For our search strategy, a combination of key words regarding lung cancer survival and SES was applied. Key words related to SES were for example: socioeconomic, deprivation, disparit^*^, segregation, education, income, occupation, [social AND (status OR class OR position OR inequality^*^)]. The detailed search strategies for all databases including the respective thesaurus terms are displayed in Table S1. In addition, reference lists of included papers have been searched.

### Inclusion and exclusion criteria—population

To be eligible, studies had to investigate a population of patients with a primary diagnosis of lung cancer. If other cancer sites were additionally investigated, studies were only included if results for lung cancer patients were reported separately.

### Inclusion and exclusion criteria—exposure(s)

We focused our search on the main socioeconomic factors education, income and occupation as explanatory variable, measured either on an individual or area-based level. As many area-based studies used combined SES measurements, also called indices, we additionally included all combined measures or indices. Categorical and continuous measurements of socioeconomic measures were included.

### Inclusion and exclusion criteria—outcome

The primary outcome of interest is survival after lung cancer diagnosis reported stratified by socioeconomic group. We focused on effect estimates from survival regression models (Cox or Poisson), 1-, 3-, or 5-year survival rates and median survival time after diagnosis. Other measures of survival were additionally included. The description of our results in the text focused on the regression models and 5-year survival rates.

### Inclusion and exclusion criteria—types of studies

Observational studies published in a peer-reviewed journal in English or German language were eligible for inclusion in our review. Non-original articles, such as guidelines, comments, book-chapters, editorials, reviews, and methods-papers were excluded. There was no further restriction regarding the period of publication or the study design.

### Inclusion and exclusion criteria—meta-analysis

To be eligible for inclusion in our meta-analysis, included studies had to fulfill further criteria. First, a study had to report hazard ratios including respective 95 % confidence intervals. Second, the studies should report on the same socioeconomic measure in a comparable manner to be able to combine the results in a meta-analysis. Third, socioeconomic measures had to be reported as categorical variables to identify low SES and high SES groups. Lastly, studies had to have a quality score of at least 6 out of 8 stars (for definition of the score see quality assessment below). This criterion was defined after writing the review protocol but before study results were summarized and interpreted. A cut-off of 6 was chosen by trading off the aim to include as many studies as possible against the aim to guarantee a high quality of the included studies. However, we additionally conducted sensitivity analyses including all studies irrespective of the quality score. In case of overlapping populations, we decided to hierarchically include the study with the most comprehensive inclusion of all stage groups, the longest period of diagnosis, and the longest follow-up period.

### Study selection and data extraction/screening

Titles, abstracts, and full texts retrieved were screened by one reviewer (IF). If no full text was available, studies were excluded if published before 1980, otherwise retrieved from The German National Library of Medicine (ZB MED) (26). EndNote software X7 was used to remove duplicates, retrieve full text articles, and manage citations. Data extraction of relevant information from included studies was performed by at least two reviewers for each study (IF, LW, and GB). Disagreements were resolved through discussion with a fourth member (LJ) of the review team. If relevant information was not reported in a study, the corresponding author was contacted via email. Sixteen authors were contacted and 10 answered to our request. Data items extracted from articles included the following: First author, publication year, country, study type, study setting, sample characteristics (n, age, gender), measure of SES (education, income, occupation, index), level of measurement (individual/area-based), outcome measure, prognostic factors, risk of bias evaluation and main results. If a study used two different SES measurements separately, results for both measures were extracted. Model results were reported for the full model including all adjustments.

### Quality assessment

To assess the methodologic and reporting quality of included studies, a modified version of the Newcastle-Ottawa-Scale (NOS) was used (27). The NOS consists of seven items to judge the quality of a study regarding the selection and comparability of study groups and ascertainment of the outcome (cohort studies) or exposure (case-control studies). One star was awarded for each item, except the comparability item which was modified so studies controlling for age in their analysis were awarded with one star and one additional star if any other factor was controlled for. In total, a study could be awarded with a maximum of 8 stars. We did not restrict the coding manual to a specific follow-up length, as the assessment of an adequate follow-up period refers to the study aim of the respective article. For example, if a study reported 3 months survival rates, the follow-up period had to be at least 3 months. The coding manual of our modified NOS can be found in the Supplementary Material.

### Statistical analysis and sensitivity analysis

We computed random effects models and assessed heterogeneity across studies by using *I*^2^ and Q statistics (28). The inverse variance method was used to assign the weight of each study in the analysis. For each study, we compared hazard ratios of the lowest SES group with the highest SES group as a reference. This was necessary as the categorizations of socioeconomic measures were very heterogeneous between the studies. Subgroup analyses were performed if possible by adjustment for smoking status, stage, and treatment. To assess the possible risk of bias and heterogeneity across studies included in our meta-analyses, we generated funnel plots and performed Begg's and Egger's test of plot asymmetry. All analyses were performed in the R statistical software (version 3.3.1) by using the metafor library (version 2.0-0).

## Results

### Study selection and characteristics

**Figure 1:**
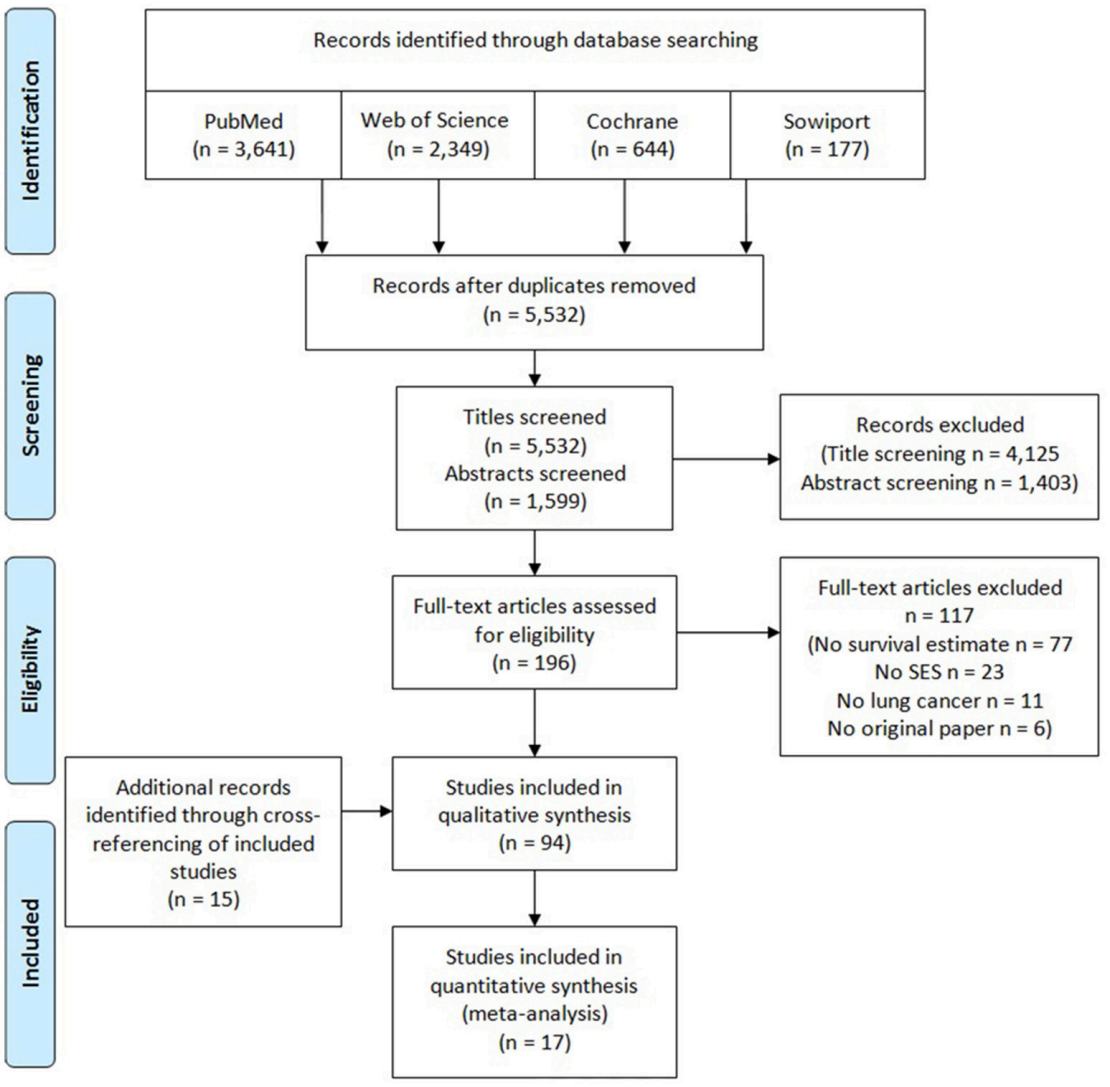
PRISMA flow diagram of study selection process for a systematic review and meta-analysis on socioeconomic differences and lung cancer survival.

Based on our search strategy, the initial search resulted in 5,532 publications potentially relevant for the systematic review (Figure 1). After title and abstract screening, 196 articles were selected for full-text screening. Assessment of the full-texts led to the exclusion of 117 articles, mainly due to not investigating survival after lung cancer or not using a measure of education, income, occupation or an index. Fifteen publications were identified by reviewing of reference lists of included articles (29–43). In total, 94 articles (5, 6, 22, 29–119) were included in the qualitative synthesis and 17 (44–48, 54–56, 60–62, 88, 90, 98, 112, 114, 115) of these were eligible to be included in the meta-analyses.

Characteristics of included studies are shown in Tables 1 and 2. There were 23 studies (30, 32, 39, 42, 44–62) reporting on socioeconomic measures on individual level (Table 1), 70 studies (5, 6, 22, 29, 31, 33, 35–38, 40, 41, 43, 63–119) reporting on area-based level (Table 2) and one study reporting on both levels (34) (Table 2). One study included both individual and aggregated measures and performed a multilevel analysis (34) (Table 2). Most studies have been published within the last 10 years. Studies on individual SES measures used mostly data from Scandinavia, the United States (US) and Italy, while the majority of studies including area-based SES measures used data from the US, Great Britain and Australia/New Zealand. Data sources for cancer survival were usually national cancer registries but also cohort studies and clinical trials (50, 53). Most studies reported on all types of lung cancer, but 20 studies restricted analyses to non-small-cell lung cancer (NSCLC) patients (5, 34, 44, 45, 50, 56, 63, 66, 68, 72, 76, 80, 88–90, 93, 96, 97, 101, 112, 115) and three studies were restricted to small-cell lung cancer (SCLC) patients (6, 92, 114).

**Table 1:** Characteristics of included studies with individual measurements of socioeconomic status.

**Paper, Data source^1^**	**Country**	**Years of diagnosis, Follow-up length, Age (range)**	**Sample size^2^**	**SES indicator(s)**^**3**^				**Outcome**						**Adjustment**^**4**^					**QS**
									**Survival**									
				**Education**	**Income**	**Occupation**	**Index**	**HR**	**Median**	**Year**	**OS**	**RS**	**CSS**	**Age**	**Sex**	**Stage**	**Smoking**	**Other**
**EUROPE, NORTH**																			
Dalton et al. (49), PBC (REG)	Denmark	1994–2003, FU: 2006, 30–79 yrs	21,492	3	3	3.6^5^				1,5,KM		X		X	X				8
Dalton et al. (48), PBC (REG)	Denmark	2004–2010, FU: 2011, 51–81 yrs	13,045	3	3			X			X			X	X	X		X^a^	8
Pokhrel et al. (39), PBC (REG)	Finland	1971–2005, FU: 2005, ≥25 yrs	66,014	3						5	X	X	X	X	X				8
Kravdal (55), PBC (REG)	Norway	1955–1986, FU: 1960–1991, 50–79 yrs	NA^6^	4		14^7^		X						X	X	X		X^b^	7
Skyrud et al. (58), PBC (REG)	Norway	2002–2011, FU: 2013, ≤ 30 yrs	24,565	3	3							X^8^		X	X	X		X^c^	8
Berglund et al. (45), PBC (REG)	Sweden	1996–2004, FU: 2006, 30–94 yrs	3,370 (NSCLC)	3	2	2		X		1,3			X	X	X	X	X	X^d^	8
Hussain et al. (54), PBC (REG)	Sweden	1990–2004, FU: 2004, 30–64 yrs	17,936	4				X					X	X	X			X^e^	7
Vågerö et al. (59), PBC (REG)	Sweden	1961–1979, FU: 1979, 20–64 yrs	7,817			3				5,KM		X			X				7
**EUROPE, OTHER**																			
Grivaux et al. (52), PCo	France	2000, FU: 2005–2006, all ages	5,447			7				5	X								4
Di Maio et al. (50), CT	Italy	1996–2005 (conduction of trials), FU: median 26.3 mths, 29–86 yrs	1,680 (NSCLC)	2				X	X	KM	X				X	X		X^f^	4
Pagano et al. (56), PBC (REG)	Italy	2000–2003, FU: 2006, all ages	2,259 (NSCLC)	3				X			X			X	X	X		X^g^	8
Pastorino et al. (57), PBC (REG)	Italy	1976–1979, FU: ≥ 9 yrs, 34–85 yrs	222			3				5	X								6
Smailyte et al. (42), PBC (REG)	Lithuania	2001–2009, FU: 2009, 30–74 yrs	8,812	3						5		X		X	X				8
Aarts et al. (44), PBC (REG)	NL	1991–2008, FU: 2009, 15–75 yrs	274 (NSCLC)	4				X		1,3,KM	X			X	X	X	X	X^h^	7
**USA**																			
Chirikos et al. (30), PBC (REG)	USA	1977–1981, FU: NA, Age: NA	NA^9^		2	2				1,3,KM			X			X			8
Clement-Duchene et al. (47), PCo	USA	2003–2005, FU: 2012, all ages	3,410	3	4			X						X	X	X	X^10^	X^i^	8
Herndon et al. (53), CT	USA	1988–2001^11^, FU:2005, all ages	1,577	5				X	X	KM	X								1
**ASIA**																			
Yeole et al. (61), PBC (REG)	India	1987–1991, FU: until 1996, all ages	1,995	4				X		5		X		X		X		X^j^	8
Yeole et al. (60), PBC (REG)	India	1992–1994, FU: until 1999, all ages	1,230	4				X		5	X			X		X		X^k^	8
Fujino (51), CoS	Japan	1988–1990^12^, FU: 2003, 40–79 yrs	1,098	3				X						X	X			X^l^	5
Fujino (32), CoS	Japan	1988–1990^12^, FU: 2003, 40–79 yrs	NA			6,3,4^5^		X						X	X			X^l^	5
Yim et al. (62), RCo	Korea	2000, FU: ≤ 48 mths, all ages	261		3			X		3,KM	X		X	X	X	X		X^m^	7
Chang et al. (46), PGB (INS)	Taiwan	2002, FU: 5 years, all ages	4,698		6			X		5,KM	X			X	X	X		X^n^	8

*CSS, Cause-specific survival; CoS, Cohort study; CT, Data from clinical trials; FU, Follow-up length; HR, Hazard ratio; mths, months; INS, Insurance; KM, Study provided Kaplan-Meier-Curve(s) or other survival curves stratified by SES; NA, Not available; NL, The Netherlands; NSCLC, Non-small cell lung cancer; OS, Overall survival; PBC, Population-based cohort; PCo, Prospective cohort; QS, Quality score; RCo, Retrospective cohort; REG, Registry; RS, Relative survival; SEER, Surveillance, Epidemiology, and End Results Program; SES, Socioeconomic status; Yrs, years of age*;

1 *Data sources for survival data*;

2 *Only lung cancer patients*;

3 *Numbers indicate number of groups, excluding unknown/missing, more detail in Table S2*;

4 *Adjustment by stratification and standardization was also considered; if both model and survival rates were calculated, only model adjustments are reported*;

5 *Study assessed two (three) indicators for occupation*;

6 *Sample size: 114,000 (all cancers)*;

7 *Combination of education and occupation*;

8 *Study reported relative excess risk*;

9 *Sample size: 1,180 (all cancers, only men)*;

10 *Study included only non-smokers*;

11 *Enrollment dates*;

12 *Study start*;

a *Comorbidities, first-line treatment, performance status, period*;

b *Histologic type/grade, period/year, sub-site*;

c *Health services region, radiotherapy, surgery*;

d *Histopathology, performance status, treatment*;

e *Period*;

f *Birth cohort, histology, performance status*;

g *Comorbidity, marital status, pattern of care*;

h *Alcohol, comorbidities, period, physical activity*;

i *Race, comorbidity, insurance, health care setting, histology, surgery, chemotherapy, radiotherapy*;

j *Marital status, treatment*;

k *Marital status, religion, treatment*;

l *Area of study*;

m *Family history, out-patient-visits per month, performance status*;

n *Comorbidities, hospital characteristics, treatment modality*.

**Table 2:** Characteristics of included studies with aggregated measurements of socioeconomic status.

**Paper, Data source^1^**	**Country**	**Years of diagnosis, Follow-up length, Age (range)**	**Sample size^2^**	**SESindicator(s)**^**3**^				**SES Level^4^**	**Outcome**						**Adjustment**^**5**^					**QS**
									**Survival**									
				**Education**	**Income**	**Occupation**	**Index**		**HR**	**Median**	**Year**	**OS**	**RS**	**CSS**	**Age**	**Sex**	**Stage**	**Smoking**	**Other**
**EUROPE**																				
Chouaid et al. (70), RCo	France	2011, FU: 2013, all ages	41,115				4	Com-mune	X		1,2	X			X	X			X^a^	7
Jansen et al. (35), PBC (REG)	Germany	1997–2006, FU: 2006, ≥15 yrs	105,688				5	District			5		X^6^		X		X			8
Aarts et al. (63), PBC (REG)	NL	2001–2012, FU: 2014, all ages	5,428 NSCLC stage IV				4	PC	X	X	1	X			X	X			X^b^	8
Louwman et al. (94), PBC (REG)	NL	1997–2006, FU: NA, all ages	12,945				3	PC	X		1	X			X	X			X^c^	8
Schrijvers (106), PBC (REG)	NL	1980–1989, FU: 1991, all ages	4,591				5	PC	X		5		X		X		X		X^d^	8
Pollock and Vickers (102), PBC (REG)	England	1987–1992, FU: 1992, 40–99 yrs	22,842				10	ED			5, KM		X		X	X				8
Schrijvers (107), PBC (REG)	England	1980–1989, FU: 1992, 30–99 yrs	40,279				5	ED	X		5		X		X	X	X		X^e^	8
Berglund et al. (64), PBC (REG)	England	2006–2008, FU: 2009, 0–80+ yrs	15,582		5			LSOA	X		3, KM	X			X	X	X		X^f^	8
Nur et al. (99), PBC (REG)	England	2001–2005, FU: 2011, 15–99 yrs	145,532				5	LSOA			1,5, 10, KM		X^6^		X	X				8
Rachet et al. (40), PBC (REG)	England	1996–2006, FU: 2007, 15–99 yrs	303,422				5	LSOA					X^7^		X	X			X^g^	8
Riaz et al. (104), PBC	England	2003–2007, FU: 2008, all ages	150,939		5			LSOA			1	X				X			X^h^	7
Rich et al. (105), PBC	England	2004–2008 (data entry), FU: 2008, all ages	60,059				5	LSOA	X						X	X	X		X^i^	8
Coleman et al. (71), PBC (REG)	England/Wales	1971–1990, FU: 1995, all ages	144,604				5	ED			1,5		X		X					7
Rachet et al. (103), PBC (REG)	England/ Wales	1986–1999, FU: 2001, 15–99 yrs	392,000		5			LSOA			5					X			X^g^	6
Sloggett et al. (41), PBC	England/Wales	1981–1997, FU: 2000, ≥45 yrs	4,271			6	5	Ward/IND					X^6^		X	X			X^j^	8
Coleman et al. (31), PBC (REG)	England/Wales	1986–1990, FU: 2001, 15–99 yrs	107,317				5	Electoral ward					X^7^			X				7
Campbell et al. (29), PBC (REG)	Scotland	1991–1995, FU: 1995, all ages	19,449				5	OA			1	X								8
Shack et al. (108), PBC (REG)	Scotland	1986–2000, FU: 2004, 15–99 yrs	20,851				5	Postcode sector			5		X		X	X				8
Iyen–Omofoman et al. (86), PCo	UK	2000–2009, FU: 2009, all ages	12,135				5	OA	X	X	1,5	X								6
O'Dowd et al. (100), PCo	UK	2000–2013, FU: 3 mths, ≥30 yrs	20,142				5	OA			1,3 mth	X								6
Cheyne et al. (69), RCo	UK	2008–2010, FU: NA, 31–97 yrs	1,432			5	5	LSOA		X	1	X								4
Ellis et al. (75), PBC (REG)	UK	2001–2005, FU: 2009, ≥35 yrs	145,206				5	LSOA			1,5		X^7^			X		X		8
Forrest et al. (78), PBC (REG)	UK	2006–2009, FU: ≥ 2 yrs, all ages	22,967		5			LSOA			2	X								8
Jack et al. (87), PBC (REG)	UK	1998, FU:NA, all ages	695				5	Ward			1	X								8
Vercelli et al. (113), PBC (REG)	Europe	1990–1994, FU: ≥ 5 yrs, 65–84 yrs	657,541		X^8^			Country			5		X			X				7
Evans and Pritchard (77), PBC	Europe/USA	Europe: 1983–1985, USA: 1983–1989, FU: 1995,0–84 yrs	10 countries		X^8^			Country			5		X		X	X				8
**CANADA/USA**																				
Mackillop et al. (95), PBC (REG)	Canada	1982–1991, FU: NA, Age: NA	357,530 all cancers		5			Postal code	X		5,KM			X	X	X			X^k^	8
Booth et al. (66), PBC (REG)	Canada	2003–2007, FU: ≥ 1 year, Age: NA	12,276 NSCLC		5			Com–munity	X		3,5	X		X	X		X			8
Dabbikeh et al. (73), PBC (REG)	Canada	1993–2009, FU: 2013, all ages	122,889		5		5	EA/DA	X		5			X	X	X				8
Boyd et al. (67), PBC (REG/SEER)	Canada/USA	1987–1992, FU: 1994, ≥20 yrs	NA^9^		5			USA: CeT, Canada: EA	X		5,KM			X	X	X			X^g^	8
Gorey et al. (33), PBC (REG/SEER)	Canada/USA	Canada:1986–1992, FU: 1993, ≥25 yrs USA:1984, FU: 1991, ≥25 yrs	Canada: 58,202 USA: 76,055		3			CeT			1,5^10^					X				8
Zhang–Salomons et al. (43), PBC (REG/SEER)	Canada/USA	Canada:1989–1993, FU: 1998, ≥25 yrs USA: 1988–1992, FU: 1997, ≥25 yrs	Canada: 8,209, USA: 15,261		5			CeT	X		5			X	X	X				8
Gomez et al. (79), PBC (REG)	USA	2000–2010, FU: 2012, all ages	3,832 Chinese ethnicity				5	CBG	X	X		X			X	X	X		X^l^	8
Hastert et al. (82), PBC (SEER)	USA	2000–2002, FU: 2010, 50–76 yrs	52,186	4	5		5	CBG	X						X	X			X^m^	8
Lara et al. (92), PBC (REG)	USA	1998–2012, FU: 2013, all ages	22,863 SCLC				2	CBG	X					X	X	X	X		X^n^	8
Ou et al. (5), PBC (REG)	USA	1989–2003, FU: median 53 mths, all ages	19,702^14^ NSCLC, stage I				5	CBG	X						X	X	X		X^N^	8
Ou et al. (101), PBC (REG)	USA	1989–2003, FU: median 53 mths, all ages	19,702^14^ NSCLC, stage I				5	CBG	X	X	5,KM	X			X	X			X^o^	8
Ou et al. (6), RCo	USA	1991–2005, FU: ≥77 mths, all ages	3,428 ED-SCLC				5	CBG	X	X	1,2	X			X	X		X	X^p^	7
Caposole et al. (68), PBC (REG)	USA	1998–2012, FU:>12 yrs, all ages	3,531 NSCLC		4			CeT		X		X								6
Erhunmwunsee et al. (76), PBC (REG)	USA	1995–2007, FU: ≥2 yrs, 20–105 yrs	4,820 NSCLC	2	2			CeT		X	6,KM			X						5
Greenwald et al. (34), PBC (REG)	USA	1980–1982, FU: 1987, Mean age 67.6 yrs	78 (NSCLC, stage II)		X			Multi- level (CeT+ IND)	X						X	X				8
Greenwald et al. (80), PBC (SEER)	USA	1978–1982, FU: ≥10 yrs, ≤ 75 yrs	5,132 NSCLC		10			CeT	X		5	X			X	X			X^q^	8
Johnson et al. (88), PBC (REG)	USA	2000–2009, FU: 2011, 50–85 yrs	32,711 NSCLC	4	4			CeT	X			X			X	X	X		X^r^	8
Johnson et al. (89), PBC (REG)	USA	2000–2009, FU: 2012, 30–85 yrs	8,322 early stage NSCLC	4	4			CeT	X						X	X			X^s^	8
Lara et al. (93), PBC (REG)	USA	1998–2009, FU: 2011, all ages	114,451 NSCLC				3	CeT	X	X				X	X	X	X		X^t^	8
Lipworth et al. (38), PBC (REG)	USA	1959–1963, FU: 3 yrs, all ages	246		2			CeT			1,3		X			X				5
Niu et al. (98), PBC (REG)	USA	1986–1999, FU: 2004, all ages	64,206		4			CeT	X		5			X	X	X	X		X^u^	8
Shugarman et al. (109), PBC (SEER)	USA	1995–1999, FU: NA, ≥65 yrs	26,073		3			CeT	X						X	X	X		X^v^	7
Tannenbaum et al. (112), PBC (REG)	USA	1996–2007, FU: ≥3 yrs, 18–104 yrs	98,541 NSCLC		4			CeT	X	X	1,3,5, KM	X				X	X	X	X^w^	8
Yang et al. (117), PBC (REG)	USA	1998–2002, FU: 2006, all ages	97,046		4			CeT	X	X	KM	X			X	X	X	X	X^x^	8
Yu et al. (118), PBC (SEER)	USA	2000–2002, FU: ≥5 yrs, Age: NA	97,046				5	CeT						X						7
Khullar et al. (90), PBC (NCDB)	USA	2003–2006, FU: NA, Mean 66.0 yrs ± SD 10.33 yrs	92,929 NSCLC	4	4			Zip code	X		KM	X			X	X	X		X^y^	8
McMillan et al. (96), PBC (NCDB)	USA	2004–2012, FU: 2013, all ages	14,154 NSCLC, stage III		2			Zip code	X			X			X	X			X^z^	8
Melvan et al. (97), PBC (NCDB)	USA	2003–2011 (resection date),FU: 30 days, ≥60 yrs	215,645 NSCLC	4	4			Zip code			30day	X								6
Wen and Christakis (116), PBC (REG)	USA	1993, FU: 1999, all ages	NA				X^11^	Zip code	X					X						6
Wang et al. (114), PBC (SEER)	USA	1983–2012, FU: NA, 30–75+ yrs	293,471 NSCLC		3			County	X		1		X		X	X			X^u^	8
Wang et al. (115), PBC (SEER)	USA	1983–2012, FU: NA, all ages	56,220 SCLC		3			County	X		1,2,3,5		X		X	X			X^u^	8
**AUSTRALIA/NEW ZEALAND**																				
Bonett et al. (65), PBC (REG)	Australia	1977–1982, FU: 1983, all ages	2,934		X			CD	X					X						8
Hall et al. (81), PBC	Australia	1982–1996, FU: ≥ 5 yrs, all ages	9,080				5	CD	X		5	X			X	X			X^A^	8
Tervonen et al. (22), PBC (REG)	Australia	2000–2008, FU: 2008, all ages	26,415				5	CD/SLA	X						X	X	X		X^B^	8
Currow et al. (72), PBC (REG)	Australia	2003–2007, FU: 2008, all ages	3,040 NSCLC				5	POA	X						X	X			X^D^	8
Denton et al. (74), PCo	Australia	2001–2014 (case discussion),FU: NA, Mean age 68 ± 11 (SD) yrs	2,369				5	POA	X		5	X			X	X	X		X^E^	7
Hui et al. (84), PBC (REG)	Australia	1996, FU: ≥ 4 yrs, 32–91 yrs	526				5	POA		X	KM	X	X							6
Stanbury et al. (110), PBC (REG)	Australia	1991–2008, FU: 2008, 15–89 yrs	33,942				5	LGA			5		X^6^		X	X	X		X^F^	8
Yu et al. (119), PBC (REG)	Australia	1992–2000, FU: 2001, 15–89 yrs	15,251				5	LGA			5		X^6^		X	X	X		X^F^	8
Jeffreys et al. (36), PBC (REG)	NZ	1994–2003, FU: 2004, 15–99 yrs	13,643				4	MB					X^7^		X					7
Sutherland and Aitken (111), RCo	NZ	1997–1999, FU: ≥ 5 yrs,27–92yrs	102				10	MB			X^12^	X								4
Haynes et al. (83), PBC	NZ	1994–2001, FU: 2004, Mean age 69 yrs	12,420				4	CAU	X						X	X	X		X^G^	8
**ASIA**																				
Ito et al. (85), PBC (REG)	Japan	1993–2004, FU: ≥ 5 yrs,Age:NA	39,621				5	Cho-Aza			1,5		X^13^			X			X^H^	7
Kwak and Kim (91), PBC (REG)	Korea	2010–2011, FU: 2014, all ages	1,426				4	Dong	X		1,3,5	X			X	X	X	X	X^L^	8
Kwak (37), PBC (REG)	Korea	2000–2011, FU: 2013, all ages	13,801				4	Dong	X	X	1,2,3, KM	X			X	X				8

*BMI, Body mass index; CAU, Census Area Unit; CBG, Census block group; CD, Census Collection District; CeT, Census tract; CSS, Cause-specific survival; DA, Dissemination area; EA, Enumeration area; ED, Enumeration district; ED-SCLC, Extensive disease small-cell lung cancer; FU, Follow-up length; HR, Hazard ratio; IND, Individual; IQR, Inter quartile range; KM, Study provided Kaplan-Meier-Curve(s) or other survival curves stratified by SES; LGA, Local Government Area; LSOA, Lower Super Output Area; MB, Meshblock; mth(s), month(s); NA, Not available; NCDB, National Cancer Data Base (American Cancer Society); NL, The Netherlands; NSCLC, Non-small cell lung cancer; NZ, New Zealand; OA, Output area; OS, Overall survival; PBC, Population-based cohort; PC, Postal code; PCo, Prospective cohort; POA, Postal code area; QS, Quality score; RCo, Retrospective cohort; REG, Registry; RS, Relative survival; SCLC, Small-cell lung cancer; SEER, Surveillance, Epidemiology, and End Results Program; SES, Socioeconomic status; SLA, Statistical Local Area; UK, United Kingdom; wks, Weeks; yrs, Years of age*;

1 *Data sources for survival data*;

2 *Only lung cancer patients*;

3 *Numbers indicate number of groups, excluding unknown/missing*;

4 *More details about SES levels in Table S2*;

5 * Adjustment by stratification and standardization was also considered; if both model and survival rates were calculated, only model adjustments are reported*;

6 *Study reported relative excess risk*;

7 *Study reported deprivation gap*;

8 *Rank order of % Gross domestic product expenditure on health*;

9 *Sample size for all cancers: USA n = 486,327, Canada n = 187,650*;

10 *Study reported survival rate ratios*;

11 *SES index has been measured on a continuous scale*;

12 *Survival rate: 6 weeks, 3 and 6 months, 1, 2, and 5 years, according to correspondence with author*;

13 *Study reported net survival*;

14 *Studies included same patient population*;

a *Comorbidities, population density*;

b *Chemotherapy, comorbidity, grade, histology, location of metastasis, period*;

c *Presence of concomitant diseases*;

d *Follow-up period, histology, treatment*;

e *Follow-up period, period of diagnosis*;

f *Comorbidity, resection, radiotherapy, chemotherapy*;

g *Year of diagnosis*;

h *Urban/rural*;

i *Histology, performance status*;

j *Marital status, north/south geographic zone, period of diagnosis, year of follow-up*;

k *Cancer center catchment area, year of diagnosis*;

l *Cancer center, chemotherapy, health insurance, histologic subtype, marital status, nativity, neighborhood ethnic enclave, radiation, surgery type, urban/rural, year of diagnosis*;

m *Race/ethnicity, marital status*;

n *Race, treatment, urban/rural, year of diagnosis*;

o *Chemotherapy, ethnic origin, histologic grade, histology, marital status, radiation, surgery, tumor lobar location*;

p *Chemotherapy, ethnicity, marital status, radiation, surgery*;

q *Race, surgery*;

r *Race, treatment, tumor grade*;

s *Elderly concentration, place of residence, race, racial segregation, random census tract effect, surgery, tumor grade*;

t *Histology, race, treatment, urban/rural, year of diagnosis*;

u *Race*;

v *Urban/rural, race, marital status, Medicaid, comorbidity, year of diagnosis, treatment, English speaking, health professional shortage area, health care provider supply characteristics*;

w *Comorbidities, geographic location, grade, histological type, hospital volume, insurance status, lymph node status, marital status, race/ethnicity, teaching hospital, treatment*;

x *Comorbidities, grade, insurance, lymph node status, race/ethnicity, tumor size, histology, surgery, radiation, chemotherapy*;

y *Comorbidity, facility type, grade, histology, insurance, lymph nodes, primary tumor site, race, radiation before surgery, surgery, urban/rural, year of diagnosis*;

z *Comorbidity, distance between residence and hospital, facility type, grade, histology, insurance, race, TN classification, tumor location, tumor size, chemotherapy, radiation fractions, radiation treatment time*;

A *Calendar period, comorbidity, histology, indigenous status, insurance status, location/status of hospital, marital status, remoteness, surgical status*;

B *Country of birth, remoteness, year of diagnosis*;

D *Comorbidity, country of birth, histology, insurance status, local health districts, lung location, remoteness, resection*;

E *Place of residence*;

F *Follow-up year*;

G *Ethnic group, travel to primary care, travel to cancer center*;

H *Period of diagnosis*;

L *BMI, diagnosis path (by regular checkup, by chance, by symptom), drinking*;

N *Chemotherapy, ethnic origin, histologic grade, histology, radiation, surgery, tumor lobar location, tumor size, period of diagnosis*.

Regarding individual socioeconomic status, 16 studies measured educational attainment, eight studies measured income and eight studies assessed the occupation of the patients. Studies investigating area-based SES most often used an index (42 studies) or income measures (30 studies) with diverse levels of aggregation from postal codes in The Netherlands (~8–17 households) (63, 94, 106) to comparisons of whole countries (77, 113). More details and definitions of socioeconomic measures and aggregated levels are provided in Table S2.

### Association of individual SES and survival—modeling results

**Figure 2:**
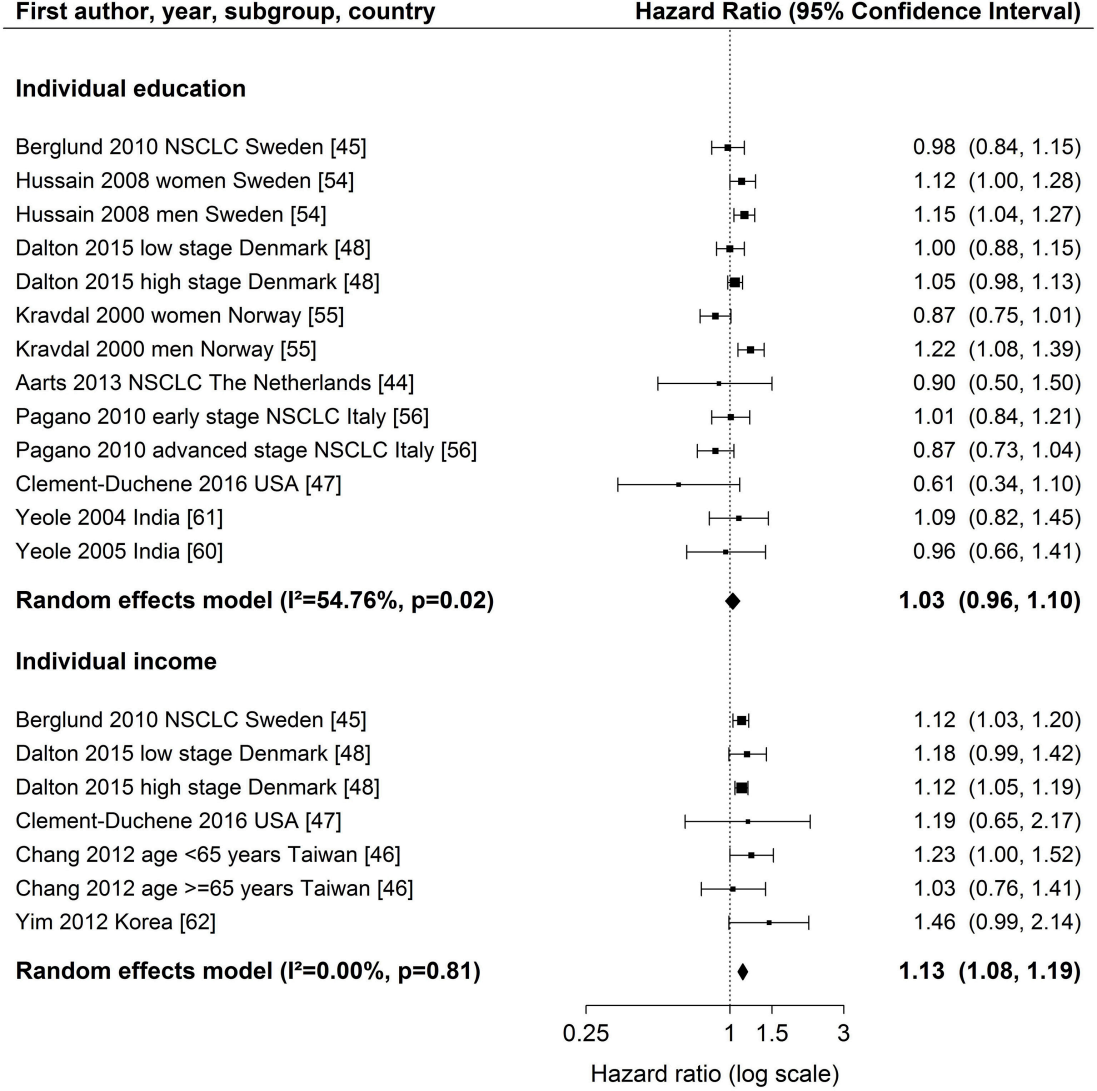
Meta-analyses of studies on the association of individual education / income (reference: high income/education) and survival after lung cancer. NSCLC, non-small cell lung cancer. Kravdal (55): highest educational group, men = 17+ years, women = 13–17+ years. Chang et al. (46): high income category = high individual AND high neighborhood income (reference), low income category = low individual AND low neighborhood income.

Detailed modeling results for all studies with individual measures are displayed in Table S3. The majority of studies adjusted for age, gender, stage, and treatment. Three studies adjusted for smoking (44, 45, 47) (Table 1). Overall, there was no consistent difference in survival between studies with different levels of adjustment for prognostic factors (Figure 2).

For individual education (Figure 2), nine studies (44, 45, 47, 48, 54–56, 60, 61) were included in the meta-analysis. The summary estimate from the random effects model revealed no association between education and lung cancer survival (hazard ratio (HR) 1.03, 95 % confidence interval (CI): 0.96–1.10). The results of these studies were rather heterogeneous (*I*^2^ = 54.76 %, *p* = 0.02). A stratified meta-analysis by stage at diagnosis was possible with three studies (45, 48, 56), but no significant associations were observed (early stage: HR 1.03, 95 % CI 0.92–1.15; late stage: HR 0.94, 95 % CI 0.81–1.08; Figure S9). We conducted stratified meta-analyses for studies that included stage, smoking or treatment in Cox models (Figures S2–S4). These analyses showed smaller effect estimates in studies that adjusted for stage (stage adjustment: HR 1.00, 95 % CI 0.92–1.08; no stage adjustment: HR 1.14, 95 % CI 1.05–1.23, Figure S2) or smoking status (smoking adjustment: HR 0.91, 95 % CI 0.72–1.14; no smoking adjustment: HR 1.04, 95 % CI 0.97–1.12, Figure S3), but confidence intervals were wide and overlapping. Stratified meta-analyses by studies that included treatment in Cox models did not suggest a difference in effect estimates (Figure S4). Three studies (50, 51, 53) were not included in the meta-analysis because of low scores for quality assessment. We conducted a sensitivity analysis by including these three studies into the meta-analysis. Results were similar to the main analysis (HR 1.05, 95 % CI 0.99–1.12, Figure S5).

For individual income (Figure 2), five studies (45–48, 62) were included in the meta-analysis showing a lower survival after lung cancer diagnosis for patients in the lowest income group compared to patients in the highest income group (HR 1.13, 95 % CI: 1.08–1.19). The studies were homogeneous (*I*^2^ = 0.00 %, *p* = 0.81). All studies included in the meta-analysis of individual income adjusted for stage (Table 1). A stratified meta-analysis by smoking adjustment gave similar estimates as for the main analysis (smoking adjustment: HR 1.12, 95 % CI 1.03–1.22; no smoking adjustment: HR 1.14, 95 % CI 1.07–1.20, Figure S6). Exclusion of one study not adjusting for treatment (62) resulted in a marginal change of estimate (HR 1.13, 95 % CI 1.08–1.18, Figure S7). One study was not included in the meta-analysis because of reporting on a continuous scale (34) and indicated an association between higher income and lower risk of death after lung cancer diagnosis (Table S3).

Individual occupation was investigated in three studies (32, 45, 55) (Table S3). As the measures were very heterogeneous, a meta-analysis was not possible. In summary, no lower survival with decreasing SES was reported for occupational groups. Fujino (32) conducted analyses stratified by gender and reported a higher risk of dying after lung cancer diagnosis for housewives (women) and unemployed women compared to employed women but he did not consider other confounding factors besides gender. Kravdal (55) stratified occupational groups by education and reported for the low educational group a lower risk of death in non-manual occupations and a lower survival in farmers compared to manual occupations within the same educational group (Table S3). High-level non-manual occupations with medium education had a lower risk compared to low educated manual occupations (55).

No study reported hazard ratios for the association between an individually measured SES index and lung cancer survival (Table 1).

### Association of area-based SES and survival—modeling results

**Figure 3:**
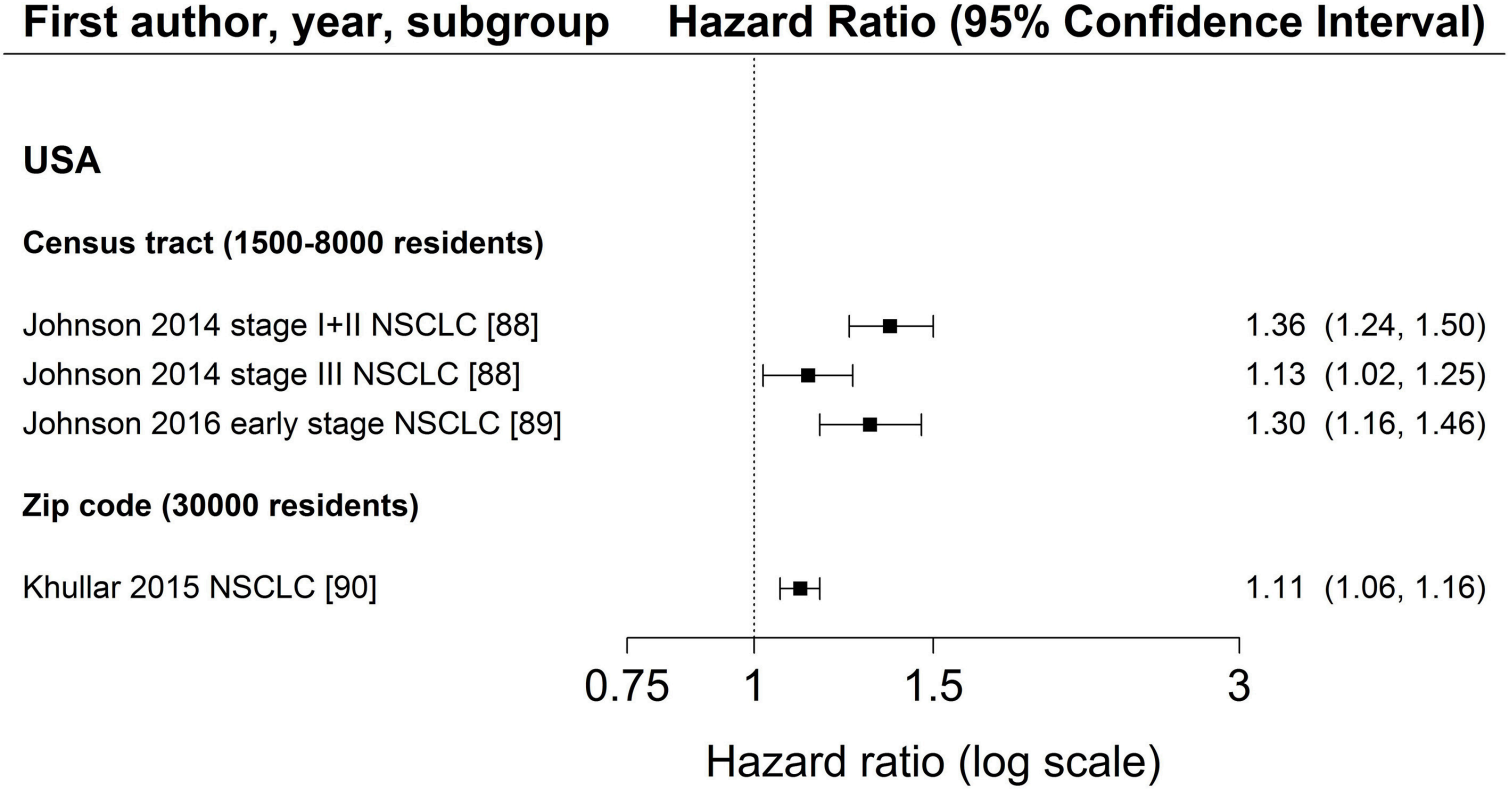
Association of area-based education (reference: high education) and survival after lung cancer. Order: small to large area level. NSCLC, non-small cell lung cancer.

**Figure 4:**
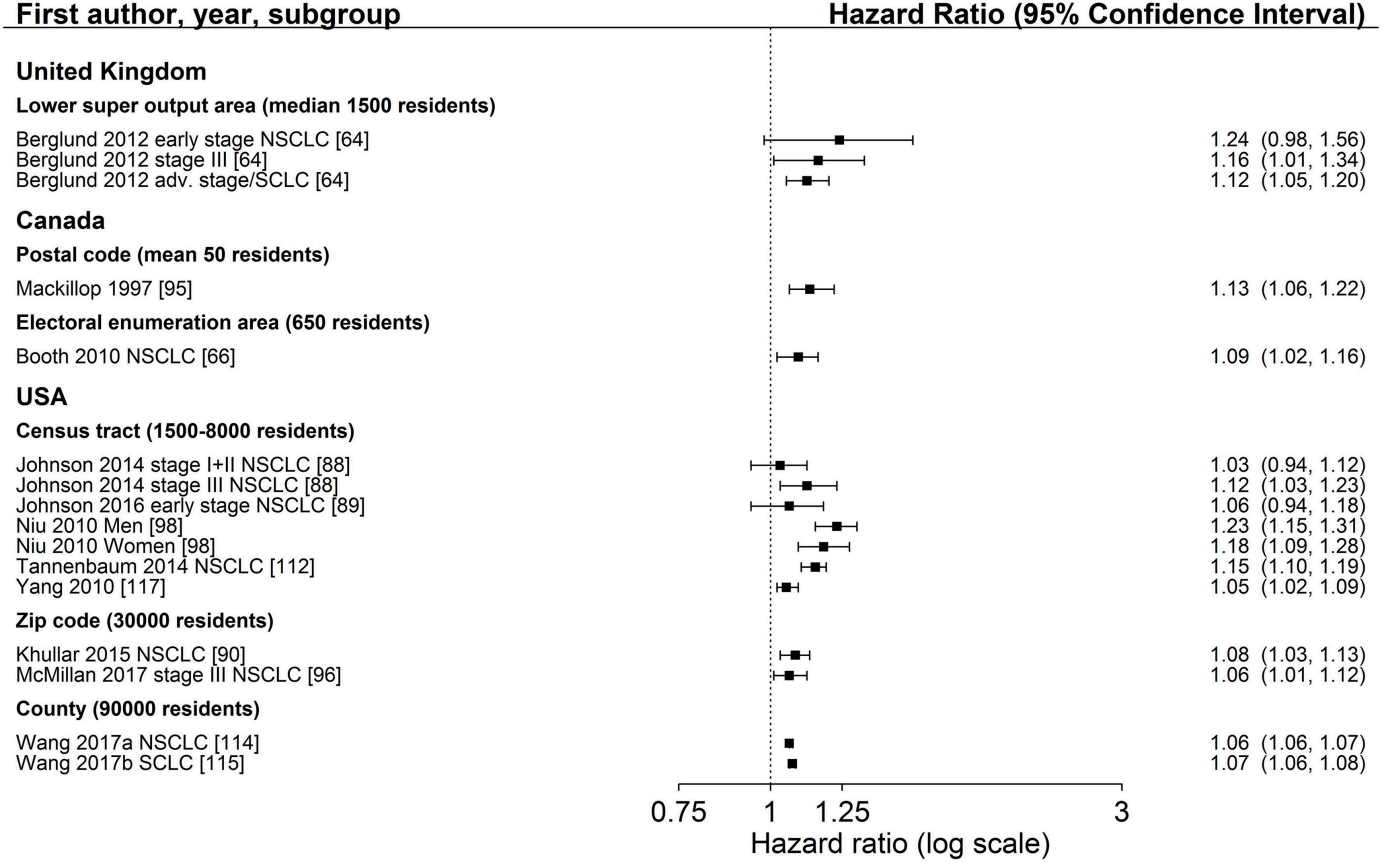
Association of area-based income (reference: high income) and survival after lung cancer. Order: small to large area level. NSCLC, non-small cell lung cancer; SCLC, small cell lung cancer.

**Figure 5:**
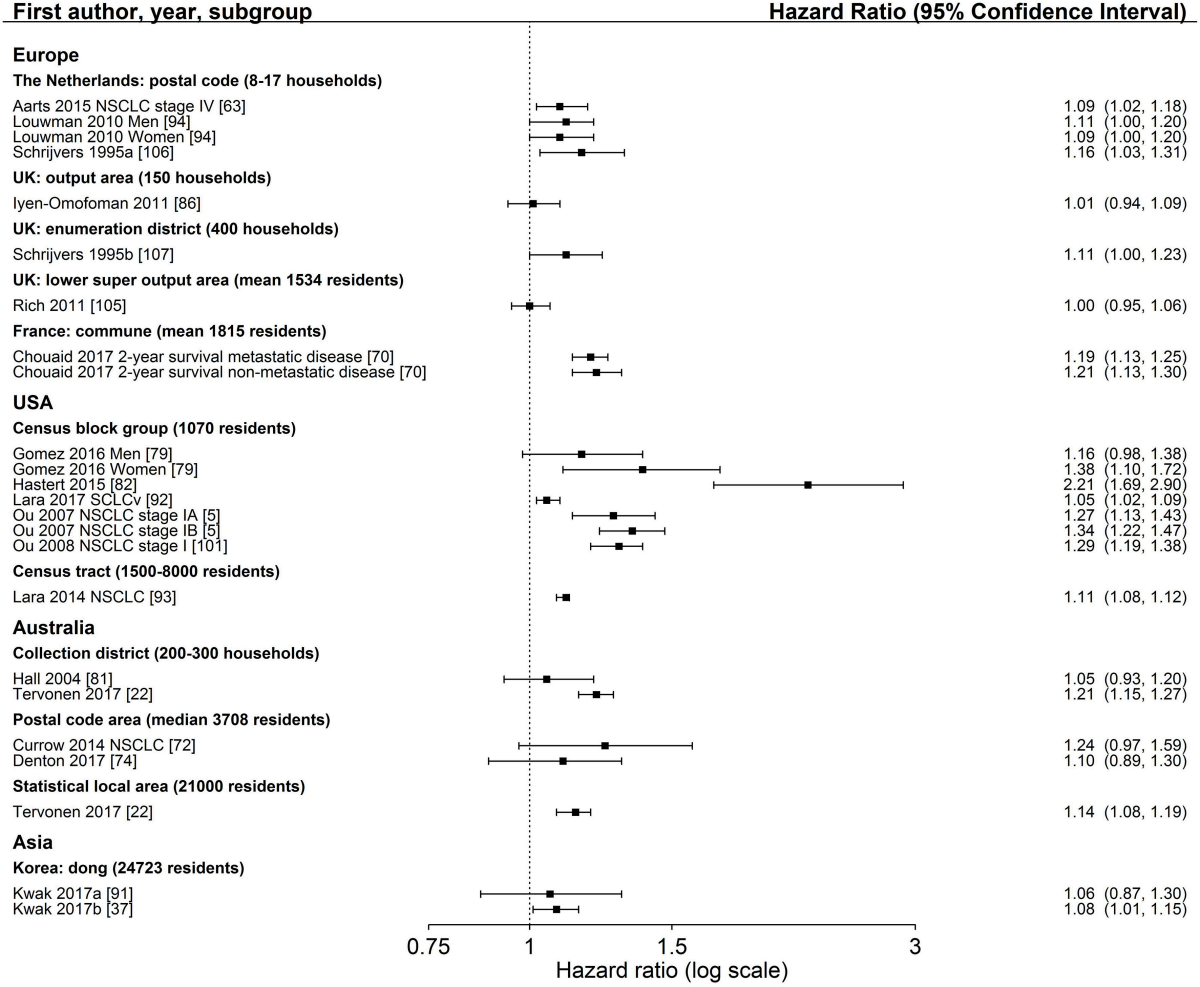
Association of area-based index measures (reference: high socioeconomic group) and survival after lung cancer. Order: region and small to large area level. NSCLC, non-small cell lung cancer; SCLC, small cell lung cancer; UK, United Kingdom.

**Figure 6:**
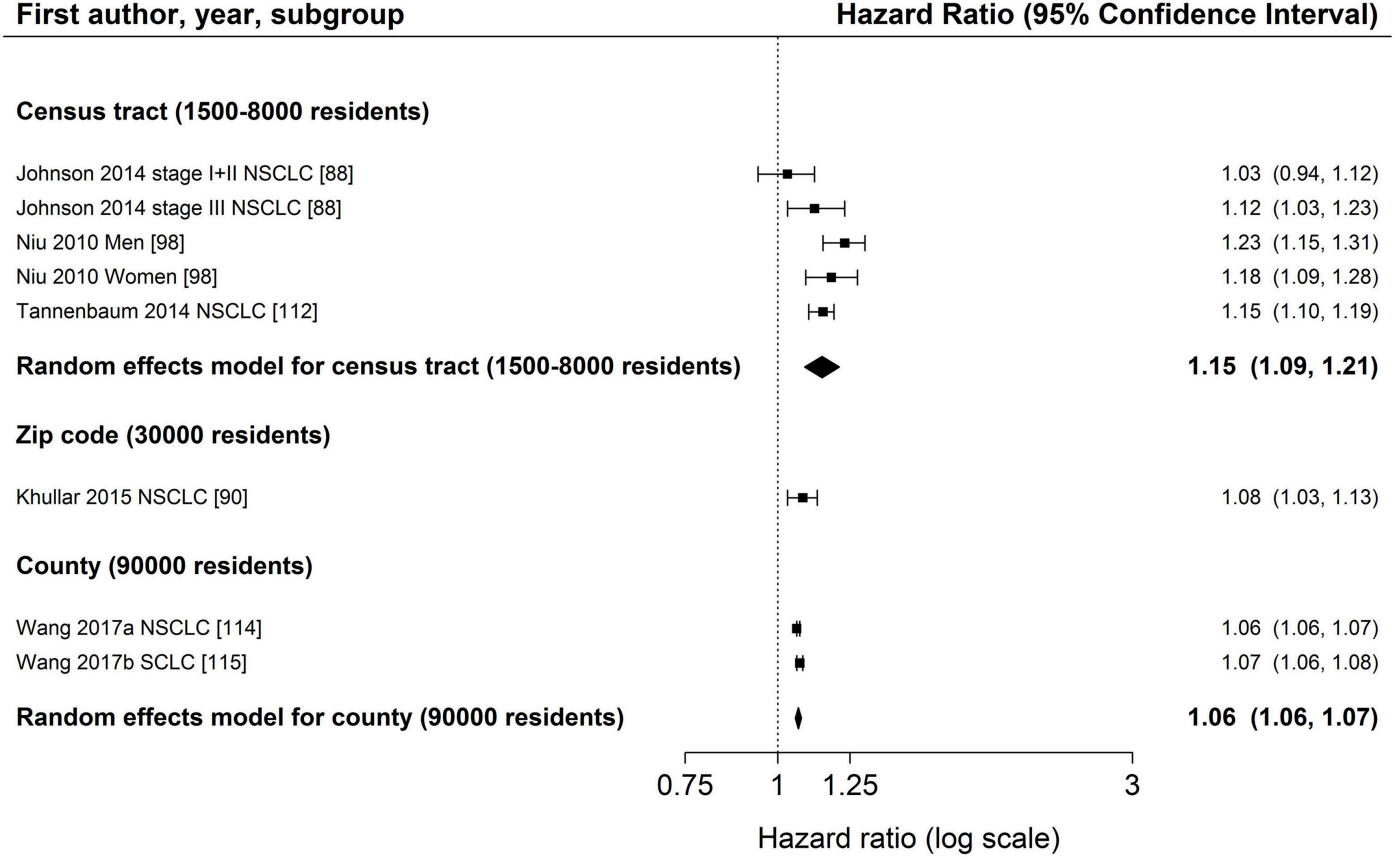
Meta-analysis of studies from the United States on the association of area-based income (reference: high income) and survival after lung cancer. Order: small to large area level. NSCLC, non-small cell lung cancer; SCLC, small cell lung cancer.

Characteristics of SES exposure of most studies on area-based SES measurements were too heterogeneous to conduct meta-analyses. However, for studies reporting hazard ratios for SES group comparisons, the hazard ratios for low SES vs. high SES (reference) are shown in Figure 3 (education), Figure 4 (income) and Figure 5 (index), sorted by region and area-level (small to large). Figure 6 additionally displays a meta-analysis for studies on area-based income from the US. Ten studies were not displayed in figures because they did not report confidence intervals (43, 73, 83, 109), did not show results (65), assessed SES on a continuous scale (6, 34, 80, 116) or did not use low or high SES as reference category (67). Results of all studies are reported in detail in Table S4.

Three studies (88–90) investigated area-based measurements of education and all reported a lower survival after lung cancer diagnosis in areas with the lowest education levels (Figure 3, Table S4). All studies adjusted for age, sex, and stage at diagnosis and included patients diagnosed with NSCLC residing in the US. The extent of the association did not depend on the size of area-level (Figure 3). Results of area-based studies were more homogeneous and reported stronger associations compared to studies investigating individual education.

The association between area-based income and lung cancer survival was investigated in 19 studies (34, 43, 64–67, 73, 80, 88–90, 95, 96, 98, 109, 112, 114, 115, 117). Twelve studies (64, 66, 88–90, 95, 96, 98, 112, 114, 115, 117) displayed in Figure 4 in general show a lower survival for the lowest income group compared to the highest group (range: HR 1.03–1.24, Figure 4). Estimates of seven studies (64, 88–90, 98, 112, 117) adjusting for stage at diagnosis were similar to estimates of studies not adjusting for stage (Table 2, Figure 4). The meta-analyses of six US studies (88, 90, 98, 112, 114, 115) revealed a slightly larger summary estimate for the smaller area-level of census tracts (HR 1.15, 95 % CI 1.09–1.21, Figure 6) than for the two larger area-levels zip code and county (zip code: HR 1.08, 95 % CI 1.03–1.13; county: HR 1.06, 95 % CI 1.06–1.07, Figure 6). However, not all of these studies adjusted for stage, which hampers their comparability. Two studies had been excluded from this meta-analysis due to overlapping study populations. The study by McMillan et al. (96) has overlapping population with the study by Khullar et al. (90). We decided to include Khullar et al. (90) in our meta-analysis as all stages were analyzed compared to McMillan et al. (96) which included solely patients diagnosed with stage III. We excluded the study by Yang et al. (117) because there is overlapping population with the study by Tannenbaum et al. (112). Although Tannenbaum et al. (112) included solely patients diagnosed with non-small cell lung cancer, they included a longer period of diagnosis compared to Yang et al. (117).

The majority of studies reported lower survival in lower income areas (Table S4).

Twenty-two studies reported hazard ratios on the association between an area-based SES index measure and lung cancer survival (5, 6, 22, 37, 63, 70, 72–74, 79, 81–83, 86, 91–94, 101, 105–107, 116) (Table S4). Group comparisons of 18 studies (5, 22, 37, 63, 70, 72, 74, 79, 81, 82, 86, 91–94, 101, 105–107) showed significant associations between lower income areas and a lower survival after lung cancer diagnosis in 10 studies (5, 22, 37, 70, 79, 82, 92, 93, 101, 106), with a range of HR 1.05–2.21 (Figure 5). Nine studies (5, 22, 74, 79, 91–93, 105, 107) adjusted for stage at diagnosis (Table 2). Notably, no study reported a hazard ratio below 1.00. Within-country comparisons did not reveal a tendency for larger or smaller estimates depending on the size of the area-level (Figure 5).

The majority of studies adjusted for age, gender and stage. Two income studies (112, 117) and two SES index studies (6, 91) included smoking status in their models (Table 2). The latter two studies reported slightly lower estimates than studies without adjustment for smoking (Table S4).

### Combined effects of individual and area-based SES—modeling results

Two studies investigated both individual and area-based SES (34, 82). However, only one study investigated directly combined effects of individual and area-based income (34). These analyses are based on a population size of *N* = 78 patients with stage II NSCLC and showed a significantly lower survival only for higher individual income. In the combined model, the area-level variable did not add any explanatory power to the model including individual income (34) (Table S4). The other study analyzed area-based SES with adjustment for individual SES in the Cox model (82). The study reported a significant association between lower area-level SES and lung cancer survival in both models with and without adjustment for individual SES (82). The estimate of the model including individual SES adjustment was considerably smaller (including individual SES: HR 1.43, 95 % CI 1.07–1.91; without individual SES: HR 2.21, 95 % CI 1.69–2.90).

### SES and survival time, survival rate, and other survival measures

Overall, 67 studies (6, 30, 33, 35, 37–39, 42–46, 49, 50, 52, 53, 57, 59–64, 66–71, 73–81, 84–87, 90, 91, 93–95, 97–104, 106–108, 110–115, 117–119) reported median survival time or survival rates after lung cancer stratified by SES (Tables S5, S6). Fifteen (30, 39, 42, 44–46, 49, 50, 52, 53, 57, 59–62) and 52 studies (6, 29, 33, 35, 37, 38, 43, 63, 64, 66–71, 73–81, 84–87, 90, 91, 93–95, 97–104, 106–108, 110–115, 117–119) used an individual or area-based SES measure, respectively. Nine individual (30, 39, 42, 45, 46, 49, 50, 59, 62) and 45 area-based (6, 29, 33, 35–38, 43, 63, 64, 66–69, 73–76, 78–81, 84–87, 90, 93–95, 97–101, 104, 106, 107, 110–112, 114, 115, 117, 118) SES studies reported lower lung cancer survival in lower SES groups (Tables S5, S6). The remaining 6 individual (44, 52, 53, 57, 60, 61) and 9 area-based (36, 70, 71, 77, 91, 103, 108, 113, 119) studies reported no difference or no gradient across socioeconomic categories in survival time or survival rates.

Forty-one studies (33, 35, 36, 39, 42, 43, 46, 49, 52, 57, 59–61, 66, 67, 71, 73– 77, 80, 81, 85, 86, 90, 91, 95, 98, 101–103, 106, 107, 110–114, 118, 119) reported 5-year survival after lung cancer diagnosis and 30 (33, 35, 36, 39, 42, 43, 46, 49, 59, 66, 67, 71, 73–76, 80, 81, 85, 90, 95, 98, 101, 106, 107, 110–112, 114, 119) of these showed lower survival rates in lower SES groups (Tables S5, S6). The range of differences between survival rates for lowest and highest SES groups was larger in studies considering area-based SES than in studies assessing individual SES (Individual SES: range 1.0–12.8 % units; area-based SES: range 0.9–22.9 % units, Tables S5, S6) but did not depend on the SES measure or the population size of the area. When we compared area-based US studies, studies using the smaller census tract level (33, 43, 67, 76, 80, 98, 112) reported larger differences in 5-year survival between high and low income areas. But those studies also observed a larger range of differences in survival rates (1.0–22.9 %) than studies assessing SES by zip codes (90) and counties (114) (range 1.2–7.7 %, Table S6).

Differences in survival between highest and lowest SES groups were similar when comparing studies reporting 1 or 3-year survival rates (1-year survival: range 1.4–11 %; 3-year survival: range 0.4–11 %, Tables S5, S6). In general, there was no distinct pattern regarding higher effect sizes in studies showing shorter survival rates.

One individual study (58) and four area-based studies (35, 41, 110, 119) calculated the relative excess risk (RER) and indicated a lower risk for higher SES groups (Tables S5, S6). Eight area-based studies (31, 36, 40, 71, 75, 85, 103, 108) used the deprivation gap which indicates the survival difference between the highest and lowest SES group and is mostly used in the UK. All of these studies reported a negative deprivation gap, meaning that the highest SES group has a higher survival rate than the lowest SES group (Table S6).

### Risk of bias

Table S7 displays the risk of bias assessment for included studies according to a modified Newcastle-Ottawa-Scale. Overall, the mean quality scores of individual and area-based studies were rather in line, both ranging from 7 to 8 out of 8 points. As the majority used data of national or regional cancer registries, many studies scored high within the categories selection and outcome, representing for example adequacy of follow-up or representativeness of study population.

Both funnel plots for the meta-analyses of individual education and income studies did not reveal any asymmetry (Education: Begg's test *p* = 0.13, Egger's test *p* = 0.07, Figure S8; income: Begg's test *p* = 0.38, Egger's test *p* = 0.34, Figure S9). The funnel plot of individual education analysis appeared to be cylindrical which might be due to the larger heterogeneity between these studies (Figure 2 and Figure S8).

## Discussion

This systematic review provides a comprehensive overview of the current literature on socioeconomic differences in lung cancer survival by including both individual and area-based measurements of socioeconomic status. Meta-analyses for individual SES and lung cancer survival revealed a weak association for studies using income measures but no consistent association for education measures. For studies using individual income measures, no consistent difference across level of adjustment for smoking status was observed and stratified meta-analyses by stage and treatment were not possible. For individual education, results indicated that adjusting for stage and smoking status might result in smaller effect estimates. Studies using occupational measures did not report lower lung cancer survival with decreasing SES. Group comparisons for hazard ratios of area-based studies indicated lower survival for lower SES irrespective of the socioeconomic measure. Meta-analyses for US studies reporting on area-based income showed a slightly larger estimate for the smaller geographical unit census tract compared to zip code and county level. However, comprehensiveness of adjustment was different across these studies. For the remaining area-based studies, the extent of association did not depend on the size of area-level but most studies reported a hazard ratio above 1.00. Compared to model results of individual SES studies, area-based studies in general reported stronger associations between SES and survival. Most studies reporting on survival time and survival rates revealed lower lung cancer survival in lower socioeconomic groups, not depending on individual or different area levels.

Compared to results for other cancer types, the association between individual income and survival after lung cancer diagnosis was weak. Cancers occurring in lung tissue are mostly detected in later stages (120) which limits opportunities for cancer therapy (121). Nevertheless, despite good treatment options for some patients, survival is still rather low (121). Given these circumstances, the effect of SES on differences in lung cancer survival might be not as relevant as for other cancer types. The smaller effect estimates for individual education studies adjusting for stage at diagnosis supports this assumption, as this cancer type is mainly diagnosed at later stages (120). For cancers of intermediate or good prognosis, such as colorectal or breast, higher relative risks were observed (10, 122).

Results of meta-analyses including individual education compared to income were rather different. This was an unexpected finding as other systematic reviews reported lower survival in low educational groups for several cancer types (20), such as breast (10) and prostate cancer (12). Furthermore, educational attainment influences occupational status which as well determines income (20). One explanation might be that many income studies were conducted in countries where income has a higher impact on access to and quality of health care; however, significant associations were as well reported in Scandinavian countries with universal health care systems.

Summary estimates of meta-analyses for individual and area-based income were similar, especially in studies using the smaller geographical unit US census tract. This was an unexpected finding as all area-based studies included in the meta-analyses were conducted in the US, a country with a non-universal health care system, and individual income studies included both types of health care systems. Therefore, we would have expected larger effect sizes for studies conducted in the United States but due to area-based measurements of income, effects might have been diluted. The comparisons of different area-level income studies revealed a slightly higher summary estimate for the smaller US census tract unit. However, not all of these studies adjusted for stage at diagnosis. Our results partly confirm results of a study comparing SES measures for different geographical units in two US states in which census tract SES measures detected gradients in all-cause mortality more consistently compared to zip code level SES measures (123). In contrast, another study examining area-based SES variables at census tract and zip code level reported small differences in effect estimates of self-rated health (124). In other countries, we could not observe larger effect sizes for studies using smaller areas consistently, but studies reported rather heterogeneously. Group comparisons of area-based studies using composite measures of SES did not reveal stronger or more consistent associations depending on the size of the geographical unit, although no study reported a HR below 1.00. This result does not confirm the discussion about the importance of the use of smaller area-levels to minimize or avoid ecological fallacy (20, 125). Due to the lack of individual index studies, it was not possible to compare area-based index studies with individual studies, thus we cannot exclude ecological bias.

One study (34) included in our systematic review investigated directly combined effects of individual and area-based income and reported the aggregated median income on US census tract level to not add any explanatory power to the model including individual income. In this study, area-based income was not valuable as proxy measure for individual income, however, it might be reasonable to interpret area-based income as its own concept, for example regarding access to health care. The study by Greenwald and colleagues (34) included only a small number (*N* = 78) of patients diagnosed with stage II lung cancer resident in the US. To further explore differences and relationships between individual and aggregated SES measures in the context of lung cancer survival, larger studies conducted in different countries are required.

The level of adjustment for prognostic factors was very heterogeneous across studies. Most studies adjusted for age, gender, and stage and many studies additionally included variables for treatment and comorbidity. Although strongly associated with lung cancer incidence, mortality, and survival (126), smoking was only considered by three individual (44, 45, 47) and five area-based studies (6, 75, 91, 112, 117). Our meta-analyses stratified by adjustment for smoking suggested lower effect estimates for individual education studies adjusting for smoking status which indicates the importance of controlling for this prognostic factor. A recent analysis confirmed the contribution of smoking to socioeconomic inequalities in mortality among 14 European countries (127). Since many individual studies, especially in Scandinavia, used cancer registry data and linked these data to other registries for the socioeconomic status, there might be no information on individual smoking status. Area-based studies using census data could have linked their data to area-based information on smoking status by other censuses or administrative sources. Such an approach should be considered in future studies.

Mechanisms that might lead to socioeconomic differences in lung cancer survival can include factors related to diagnosis, treatment modalities, and patients themselves (20). Access to health care can be both influenced by the affluence of a country or a residential area and the individual. More deprived areas can have less health care resources which could result in a delay in diagnosis and delay in start of treatment (20). However, a meta-analysis on the effect of SES on stage at lung cancer diagnosis did not reveal an association (18). The stratified meta-analysis of individual education studies in the present review did as well not show any differences which confirm the results of Forrest and colleagues (18). For cancer therapy, socioeconomic differences have been reported regarding the administration of specific treatments as well as the referral to specialists or to oncology centers (20). For instance, lung and breast cancer patients belonging to deprived groups were less frequently treated by surgery in a study from England (128). Due to the lack of studies stratifying by treatment in the present review we could not investigate this issue.

Our study has important strengths and some limitations. The current literature search was conducted in four databases, which might have missed out relevant articles. We restricted our search terms to only “lung cancer” due to the large amount of search results when using the term “cancer.” This might be the reason why the number of articles found through searching reference list of included papers was high. Nevertheless, the amount of detected literature through database search was still rather large and it was possible to include databases specialized to the social sciences to assure inclusion of articles not only indexed in biomedical science focused databases. In addition, we enhanced the quality of extracted data by contacting authors if results were not reported clearly or incompletely to give a comprehensive view of all included studies. While we cannot completely rule out the presence of a publication bias, which would lead to an overestimation of socioeconomic differences in cancer survival, our funnel plots for the meta-analyses did not reveal asymmetries suggesting that the probability of publication bias is rather low.

In general, studies were very heterogeneous, not only in the use of socioeconomic measures and aggregated levels but also in reporting of survival measures and in the level of adjustment. The studies have been conducted in several countries around the world including very different settings. The adjustment for key prognostic factors such as stage was often not possible. Thus, like in most epidemiologic studies, we cannot rule out that findings might be influenced by confounding. Furthermore, our comparisons of summary estimates across subgroups (e.g., by adjustment and aggregation level) were not based on statistical tests and observed trends might be chance findings. Thus, comparison of results across studies and the conclusions derived from this review must be interpreted with caution.

The generalizability of our results to low-income countries is limited, as they were highly underrepresented and no study from Africa or South America was found. One reason for this might be the restriction to publications in English or German language in our literature search. In our study, most individual studies were conducted in Scandinavian countries and most area-based studies were conducted in the US or United Kingdom. For other European countries as well as Asian countries, further studies are needed.

We did not carry out meta-analyses stratified by gender. Considering papers with the largest study populations included in our review, studies reported in general a higher survival in women compared to men. However, the majority of these studies also reported similar results for women and men regarding a potential gradient according to SES. This was true for both individual and aggregated SES measurements.

Although the Newcastle-Ottawa-Scale (NOS) is a tool for quality assessment of studies which is widely used, there is some critique about its validity (129). However, the NOS gives an overview of the quality of included articles and helps to exclude studies that are not suitable to be included in a meta-analysis. We excluded three studies from our meta-analyses because of a low quality score. These studies were also less comparable to the other studies due to other reasons: The first study used data from clinical trials (50) and was therefore not representative of the underlying population, the second study only reported univariate hazard ratios without adjustment (53) and the third study used data of 24 institutions which could voluntarily participate in the study (51). As the cut-off quality score was not set a priori, a sensitivity analysis including these three studies was conducted and revealed similar estimates. Another limitation was that there is no specific NOS coding manual for studies relying on registry data. We used the manual for cohort studies, therefore many registry studies were rated too low in the outcome section because they did not describe how mortality data were collected although it could be assumed that these data were retrieved by administrative sources with good quality (130). On the other hand, studies using registry data might be awarded too many points (stars) in the comparability section as their quality of measurement of potential confounders might not be as high as in usual cohort studies.

The interpretation and summary of both model and survival rate results among studies remained difficult due to diversity in SES measurements used, in particular across different countries or continents. In their review on socioeconomic differences and the risk of lung or colorectal cancer, Kuznetsov and Mielck (17) already found very heterogeneously reported SES measurements and therefore could not conduct a meta-analysis. However, we were still able to perform meta-analyses by using hazard ratios of the lowest and highest socioeconomic group which was reported by most studies. Furthermore, we focused on model results of the studies, as most studies that reported survival rates showed age-standardized rates without any further adjustment for other prognostic factors. Our restriction of using the highest and lowest SES categories for comparing the model results enabled us to conduct meta-analyses with studies assessing the SES on different categories like tertiles or quintiles. The downside of this approach is that we compared different levels of SES (e.g., the lower quintile might correspond to a lower SES as compared to the lower tertile). However, as studies reported SES measures heterogeneously, this was the only way to show summarized measures for the effect of SES on lung cancer survival.

Another limitation was that it was not possible to perform stratified meta-analyses by subtypes of lung cancer because no individual study reported on SCLC patients only. Nevertheless, meta-analyses of other important prognostic factors (stage, treatment, and smoking) were conducted and revealed no major differences compared to the main analyses.

In conclusion, the body of evidence in this review provides some support for the hypothesis that lower individual income is associated with a lower survival after lung cancer diagnosis. There was no evidence for an association between individual education or occupation and lung cancer survival. Group comparisons for hazard ratios of area-based studies indicated lower survival for lower SES groups, irrespective of the socioeconomic measure. However, effect sizes are generally smaller than and not as consistent as found for other cancer types. Future research should focus on a combined analysis of individual and aggregated SES measures, for example by constructing aggregated measures from individual data. This approach would allow to investigate associations between survival and both individual and aggregated measures, whilst also taking prognostic factors such as stage and smoking into account. Furthermore, a standardized socioeconomic measure would be desirable to enhance comparability across nations and across different levels of aggregation.

## Author contributions

Study designed by IF, LJ, and HB. Literature search performed by IF. Data extraction and quality check completed by IF, GB, and LW. Data synthesis of selected studies completed by IF. Meta-analyses performed by IF, GB, and LJ. Abstract, cover letter and manuscript drafted by IF and LJ. Abstract, cover letter, and manuscript reviewed and edited by HB, GB, and LW.

### Conflict of interest statement

The authors declare that the research was conducted in the absence of any commercial or financial relationships that could be construed as a potential conflict of interest.
