# Supplementary material for: Socioeconomic Differences and Lung Cancer Survival—Systematic Review and Meta-Analysis
Source: Front Oncol. 2018 Nov 27;8:536. doi: 10.3389/fonc.2018.00536 (PMC6277796; doi:10.3389/fonc.2018.00536)
Supplement: Supplementary file 1 [file Table_1.DOCX]

**Supplement: Table S1.** Search strategies. ^1^Alle Felder (engl. all fields)

| **Database** | **Search strategy** |
| --- | --- |
| Cochrane Library | ((MeSH descriptor: [Mortality] explode all trees) OR (MeSH descriptor: [Survival] explode all trees) OR (MeSH descriptor: [Survival Rate] explode all trees) OR (MeSH descriptor: [Disease-Free Survival] explode all trees) OR mortality OR survival)  AND  ((MeSH descriptor: [Lung Neoplasms] explode all trees) OR (MeSH descriptor: [Carcinoma, Non-Small-Cell Lung] explode all trees) OR (MeSH descriptor: [Small Cell Lung Carcinoma] explode all trees) OR (small-cell lung cancer) OR (non-small cell lung cancer) OR (lung AND (cancer OR carcinoma OR neoplasm)))  AND  (MeSH descriptor: [Healthcare Disparities] explode all trees) OR (MeSH descriptor: [Health Status Disparities] explode all trees) OR (MeSH descriptor: [Socioeconomic Factors] explode all trees) OR (MeSH descriptor: [Social Class] explode all trees) OR (MeSH descriptor: [Income] explode all trees) OR (MeSH descriptor: [Occupations] explode all trees) OR socioeconomic OR deprivation OR disparit* OR segregation OR education OR income OR occupation OR (social AND (status OR class OR position OR inequalit*)) |
| PubMed | (mortality OR survival OR (”mortality"[Subheading]) OR ("Mortality"[Mesh] OR "Survival"[Mesh] ) OR ("Survival Analysis"[Mesh]))  AND  ((lung AND (Cancer OR carcinoma OR neoplasm)) OR “small-cell lung cancer” OR “non-small cell lung cancer” OR ("Lung Neoplasms"[Mesh]))  AND  ((social AND (status OR class OR position OR inequalit*)) OR socioeconomic OR deprivation OR disparit* OR segregation OR education OR income OR occupation OR ("Healthcare Disparities"[Mesh]) OR ("Health Status Disparities"[Mesh]) OR ("Social Class"[Mesh]) OR ("Socioeconomic Factors"[Mesh]) OR ("Social Determinants of Health"[Mesh])) |
| Sowiport | (Alle Felder^1^:(survival OR mortality))  AND  (Alle Felder^1^:(lung AND cancer))  AND  (Alle Felder^1^:(socioeconomic OR deprivation OR social OR segregation OR education OR income OR occupation)) |
| Web of Science | (survival OR mortality)  AND  ((lung AND (cancer OR carcinoma OR neoplasm)) OR “small-cell lung cancer” OR “non-small cell lung cancer”)  AND  (socioeconomic OR deprivation OR disparit* OR segregation OR education OR income OR occupation OR (social AND (status OR class OR position OR inequalit*))) |
