# Supplementary material for: Socioeconomic Differences and Lung Cancer Survival—Systematic Review and Meta-Analysis
Source: Front Oncol. 2018 Nov 27;8:536. doi: 10.3389/fonc.2018.00536 (PMC6277796; doi:10.3389/fonc.2018.00536)
Supplement: Supplementary file 2 [file Table_2.docx]

**Supplement: Table S2.** Detailed description of socioeconomic status indicators of included studies. Abbreviations: ACORN = A geodemographic segmentation tool; GDP = Gross domestic product; Pop. = Population; Q1-Q4 = quartiles; Q1-Q5 = quintiles; SEIFA = Socioeconomic Indexes for Areas; SES = Socioeconomic status; Yrs = years of age; ^1^Information retrieved from Forrest 2015; ^2^Information retrieved from Erhunmwunsee 2012; ^3^Information retrieved from Hui 2005 and Australian Bureau of Statistics; ^4^Information retrieved from Booth 2010; ^5^Correspondence with author; ^6^Information retrieved from Hastert 2015; ^7^Information retrieved from Krieger 2002; ^8^Information retrieved from Iyen-Omofoman 2011; ^9^Information retrieved from Sun 2014 and US Census Bureau; ^10^Information retrieved from Stanbury 2016; ^a^Based on four census variables: male unemployment, lack of car ownership, overcrowding, and low social class; ^b^Based on six domains: income, employment, health deprivation and disability, education skills and training, housing, and geographical access to services; ^c^Based on several area-level variables, for example: education, English speaking, employment, marital status, car ownership, housing, employment, income; ^d^Based on four variables: unemployment, non-car ownership, non-home ownership, household overcrowding

| **Paper** | **Level, country**  **(approximated size)** | **SES Indicator(s)** | | | |
| --- | --- | --- | --- | --- | --- |
|  |  | **Education** | **Income** | **Occupation** | **Index** |
| Aarts 2013 [44] | Individual | Level 1: primary school only,  Level 2: lower vocational and lower secondary school,  Level 3: intermediate vocational and intermediate/higher secondary school,  Level 4: higher vocational school and university |  |  |  |
| Aarts 2015 [63] | Postal code, The Netherlands  (8-17 households) |  |  |  | House value and household income: low, intermediate, high SES, institutionalized, unknown |
| Berglund 2010 [45] | Individual | Low: ≤ 9 yrs /mandatory school,  Middle: 10-12 yrs/ high school,  High: ≥ 13 yrs/ post high school /college /university | Lowest 50% and highest 50% household disposable income | Low: blue collar and low level white collar workers,  High: intermediate and high level white collar workers and the self-employed,  Unknown: no employment or missing |  |
| Berglund 2012 [64] | Lower super output area level, England  (Median pop. = 1534)^1^ |  | Based on income domain of 2007 Indices of Deprivation,  quintiles  Q1: most affluent,  Q5: most deprived |  |  |
| Bonett 1984 [65] | Collection district, Australia  (200-300 dwellings) |  | Median male income |  |  |
| Booth 2010 [66] | Electoral enumeration area, Canada  (max. 650 residents) |  | Median household income  quintiles  Q1: $24000  Q2:$31000  Q3:$36000  Q4:$44000  Q5:$55000 |  |  |
| Boyd 1999 [67] | Census tract, USA (Median pop. = 3800)  County level, USA  (Median pop. = 19000)  Census enumeration area, Canada  (Median pop. = 700)  Census subdivision, Canada  (Median pop. = 9000) |  | Median household income  quintiles  Q1: wealthiest 20%  Q5: poorest 20 % |  |  |
| Campbell 2000 [29] | Output area, Scotland (Median pop. = 124 households) |  |  |  | Carstairs index^a^  quintiles  Q1: least deprived  Q5: most deprived |
| Caposole 2014 [68] | Census tract, USA  (Pop. range 1500-8000)^2^ |  | Poverty status in past 12 mths  SES 1: <5 %  SES 2: 5-10 %  SES 3: 10-20 %  SES 4: >20 % |  |  |
| Chang 2012 [46] | Individual |  | Individual and aggregated level  Individual  Income-related insurance payment  High: ≥ US$1142 per month (NT$40001);  Moderate: US$571–1141 per month (NT$20000–40000);  Low: < US$571 per month (NT$ 20000);  NT$ = New Taiwan Dollar  Neighborhood  Advantaged: higher-than median household income,  Disadvantaged: lower-than median household income |  |  |
| Cheyne 2013 [69] | Lower super output area level, UK  (Median pop. = 1534)^1^ |  |  | ACORN group:  WA wealthy achievers, UP urban professional, CO comfortably off, MM moderate means, HP hard pressed | Index of multiple deprivation (IMD)^b^  quintiles  Q1: least deprived  Q5: most deprived |
| Chirikos 1984 [30] | Individual |  | Continuous and categories:  High: >$12999  Low:<$6000 | White collar/blue collar |  |
| Chouaid 2017 [70] | Commune, France  (Mean pop. = 1815) |  |  |  | Social deprivation index (SDI): unemployment rate, median household income, % high school graduates in adult population, % blue-collar workers in active population  quartiles  Q1: most deprived  Q2: deprived  Q3: privileged  Q4: most privileged |
| Clement-Duchene 2016 [47] | Individual | Not applicable, some grade school, high school graduate, some college or more, missing | Annual household income  less than $20000, $20000-$40000, $40000-$60000, $60000 or more, missing |  |  |
| Coleman 2001 [71] | Census enumeration district, England/Wales  (Mean pop. = 500) |  |  |  | Carstairs index^a^  quintiles  Q1: affluent  Q5: deprived |
| Coleman 2004 [31] | Electoral ward, England  (Mean pop. = 6600) |  |  |  | Carstairs index^a^ (patients diagnosed 1986-95)  IMD income domain (patients diagnosed 1996-99) |
| Currow 2014 [72] | Postal code area, Australia  (Median pop. = 3708)^3^ |  |  |  | SEIFA Index of Relative Socio-Economic Disadvantage^c^,  quintiles  Q1: least disadvantaged  Q5: most disadvantaged |
| Dabbikeh 2017 [73] | Enumeration area  (max. 650 residents)  dissemination area  (400-700 residents),  Canada^4^ |  | Gross median household income Quintiles  Q5: wealthiest 20%  Q1: poorest 20%  and categories  $20000, $40000, $60000, $80000, $100000 |  |  |
| Dalton 2008  [49] | Individual | Basic/high school, vocational education, higher education, unknown | Household income of taxation and interest per person  Low: 1st quartile,  Medium: 2nd-3rd quartile,  High: 4th quartile | *Occupation 1*  Working, unemployed, early retirement pension  *Occupation 2*  Creative core, creative professionals, bohemians, service class, manual class, agricultural class, unknown |  |
| Dalton 2015 [48] | Individual | Short: mandatory education of up to 7 or 9 yrs for people born before or after 1958, respectively,  Medium: between 8-10 and 12 yrs, last grades of primary /secondary/vocational education,  Higher: > 12 yrs | Household income of taxation and interest per person,  Low: 1st quartile,  Medium: 2nd-3rd quartile,  High: 4th quartile |  |  |
| Denton 2017 [74] | Postal code area, Australia  (Median pop. = 3708) |  |  |  | SEIFA Index of Relative Socio-Economic Disadvantage^c^,  quintiles  Q1: least disadvantaged  Q5: most disadvantaged |
| Di Maio 2012 [50] | Individual | High: at least high school diploma,  Low: less than high school diploma |  |  |  |
| Ellis 2014 [75] | Lower super output area level, UK  (Median pop. = 1500)^5^ |  |  |  | Index of multiple deprivation (IMD)^b^  quintiles  Q1: least deprived  Q5: most deprived |
| Erhunmwunsee 2012 [76] | Census tract, USA  (Pop. range 1500-8000) | Education 1: % at least high school,  Education 2: % bachelor´s degree, quartiles | Income 1: % living below poverty line,  Income 2: median household income, quartiles |  |  |
| Evans and Pritchard 2000 [77] | Country level, Europe/USA |  | % GDP expenditure on health |  |  |
| Forrest 2015 [78] | Lower super output area level, UK  (Median pop. = 1534) |  | Based on income domain of Index of Multiple Deprivation^b^ 2007 + 2010  quintiles  Q1: least deprived  Q5: most deprived |  |  |
| Fujino 2007a [51] | Individual | Educational background:  ≤15, 16-18, ≥19 |  |  |  |
| Fujino 2007b [32] | Individual |  |  | Type of employment: employed, part time, self-employed, housewife, unemployed, others  Type of jobs 1: office work, manual work, others  Type of jobs 2: sedentary work, sedentary and standing, standing position, moving |  |
| Gomez 2016 [79] | Census block group, USA  (Median population: 1070)^6^ |  |  |  | Index:  Education, occupation, employment, household income, poverty, rent and house values  quintiles  Q1 (low SES)  Q5 (high SES) |
| Gorey 1997 [33] | Census tract, USA/Canada  (Mean pop. USA=3661, Canada=4843) |  | Income group: high, middle, low  terciles  annual household income  Canada: low=CAD$24400;  USA: low=USD$11700  1990 Toronto-Detroit comparison on median income status by tertile, in US dollars, was as follows:  Canada:  High= $56 600  Middle= $43 300  Low= $30 400  USA:  High= $51 500  Middle= $35 700  Low= $17 800 |  |  |
| Greenwald 1994 [34] | Individual +  Census tract, USA  (Pop. range 1500-8000)^2^ | Number of years of schooling | Family income |  |  |
| Greenwald 1998 [80] | Census tract, USA  (Pop. range 1500-8000)^2^ |  | Median family income, deciles |  |  |
| Grivaux 2011 [52] | Individual |  |  | Socio-professional category: Farmer, manager, service provider, self-employed, unemployed, employee, manual worker |  |
| Hall 2004 [81] | Collection district, Australia  (200-300 dwellings) |  |  |  | SEIFA Index of Relative Socio-Economic Disadvantage^c^,  quintiles  Q1: least disadvantaged  Q5: most disadvantaged |
| Hastert 2015 [82] | Census block group, USA  (Median population: 1070) | High school graduate/ General Educational Development (GED) or below,  some college/technical school,  college graduate,  advanced degree | Annual household income  <$20 000, $20 000–39 999, $40 000–59 999, $60 000–79 999 and $80 000 or more |  | Index: log of median value of owner-occupied housing units, log of median  household income, % households receiving net rental,  interest or dividend income, % adults ages 25 and older who completed high school and who completed college, and % employed persons ages 16 and older in professional and managerial occupations  quintiles  Q1: high SES  Q5: low SES |
| Haynes 2008 [83] | Census area units, New Zealand  (approx. 2300 residents) |  |  |  | 2001 NZ deprivation index:  Car access, tenure, benefit receipt, unemployment, low income, telephone access, single parent families, qualifications, living space  Low: lowest quartile (whole sample),  Medium: quartiles two and three incorporating half the records around the median,  High: records between the 75 and 95 percentiles,  Highest: the highest 5 % of records |
| Herndon 2008 [53] | Individual | Grades 1-8, grades 9-11, high school graduate, some college, college degree |  |  |  |
| Hui 2005 [84] | Postal code area, Australia  (Median pop. = 3708) |  |  |  | SEIFA Index of Relative Socio-Economic Disadvantage^c^,  quintiles  Q1: least disadvantaged  Q5: most disadvantaged |
| Hussain 2008 [54] | Individual | <9 yrs, 9-11 yrs, 12-13 yrs, university graduate |  |  |  |
| Ito 2014 [85] | Cho-aza level, Japan  (av. Pop. = 3000) |  |  |  | Areal deprivation index (ADI)  quintiles  Q1: least deprived  Q5: most deprived |
| Iyen-Omofoman 2011 [86] | Output area, UK  (Median pop. = 150 households) |  |  |  | Townsend Index of multiple deprivation^d^  quintiles  Q1: least deprived  Q5: most deprived |
| Jack 2006 [87] | Ward level, UK  (Median pop. = 4974) |  |  |  | Index of multiple deprivation (IMD)^b^  quintiles  Q1: least deprived  Q5: most deprived |
| Jansen 2014 [35] | District level, Germany  (Median pop. = 126571) |  |  |  | German Index of Multiple Deprivation  quintiles  Q1: most affluent  Q5: most deprived |
| Jeffreys 2009 [36] | Mesh-block, New Zealand  (minimum 100) |  |  |  | New Zealand deprivation index (NZDep)  nine variables: % of people  (a) with no access to a telephone  (b) aged 18 to 59 years, receiving a means-tested benefit  (c) aged 18 to 59 years, unemployed (d) living in households with equivalized income below an income threshold  (e) with no access to a car  ( f) aged <60 years, living in a single-parent family  (g) aged 18 to 59 years, without any qualifications  (h) not living in their own home  (i) living in households below the equivalized bedroom occupancy threshold.  Deciles (four groups):  D1-D4 (least deprived)  D5+D6  D7+D8  D9+D10 (most deprived) |
| Johnson 2014 [88] | Census tract, USA  (Pop. range 1500-8000)^2^ | Educational attainment,  quartiles  Q1: high  Q4: low | Economic deprivation,  quartiles  Q1: low  Q4: high |  |  |
| Johnson 2016 [89] | Census tract, USA  (Pop. range 1500-8000)^2^ | Educational attainment,  quartiles  Q1: high  Q4: low | Economic deprivation,  quartiles  Q1: low  Q4: high |  |  |
| Khullar 2015 [90] | Zip code, USA  (Average pop.: 30000)^7^ | % adults not graduating high school: ≥29, 20-28.9, 14-19.9, <14 % | Median household income:  <$30000, $30000-$34999, $35000-45999, $46000+ |  |  |
| Kravdal 2000 [55] | Individual | Compulsory/7-9 years of schooling,  Secondary/10-12 years of schooling,  Lower-level post-secondary/normally requiring 13-16 years of schooling,  High-level post-secondary/defined as a level corresponding to at least Master`s degree and thus normally requiring 17 or more years of schooling |  | Combined occupation/education  Low education/7-12yrs: Manual (except *), Non-manual (except *) (*=hotel and restaurant workers, ship's officers, deck and engine-room crew, Farmer, Fisherman, No occupation recorded)  Medium education/13-16yrs: (Largely) manual, Non-manual (low level), Non-manual (high level, except teacher), Teacher  High education/17 or more yrs: Teacher, Physician, Others  Medium or high education: No occupation recorded |  |
| Kwak 2007a [91] +  Kwak 2017b [37] | Dong, Korea  (approx. 24723 residents) |  |  |  | Deprivation index, 8 indicators:  1. residents living alone,  2. female household head,  3. no housing ownership,  4. low education level (less than high school graduation among those aged 35-64),  5. low occupational social class (low social class among economically active household heads aged 15-64 according to occupation based social class classification),  6. the divorced or separated in marital status among those aged 15 or over,  7. population aged 65 or over,  8. unemployment among males aged 15-64; continuous and categorical  quartiles  Q1: least deprived  Q4. most deprived |
| Lara 2014 [93] | Census tract, USA  (Pop. range 1500-8000)^2,5^ |  |  |  | Composite score:  education median household income, % living 200% below poverty level, % of blue-collar workers, % older than 15 years in workforce, without job, median rent, median house value;  Lowest SES (quintiles 1, 2)  Mid SES (quintile 3)  Highest SES (quintiles 4,5) |
| Lara 2017 [92] | Census block group, USA  (Median population: 1070)^6^ |  |  |  | Neighborhood socioeconomic status (imputed): education, occupation, unemployment, household income, poverty, rent, house values  SES1(lowest), SES5 (highest) |
| Lipworth 1970 [38] | Census tract, USA  (Pop. range 1500-8000)^2^ |  | Median family income:  < $5000  >$5000 |  |  |
| Louwmam 2010 [94] | Postal code, The Netherlands  (8-17 households) |  |  |  | Individual fiscal data, economic value of the home and household income deciles: Low=deciles1-3,  Medium=deciles 4-7,  High=deciles 8-10),  separate class for long-term care providing institutions (e.g. nursing home) |
| Mackillop 1997 [95] | 64 % Postal code  (Mean pop. = 50)  32 % residence codes  (Mean pop. = 9000)  Canada |  | Median household income:  >50000, 40000-50000, 30000-40000, 20000-30000, <20000 |  |  |
| McMillan 2017 [96] | Zip code, USA  (Average pop.: 30000)^7^ |  | Median income:  <$63000, ≥$63000 |  |  |
| Melvan 2015 [97] | Zip code, USA  (Average pop.: 30000)^7^ | % adults not graduating high school: ≥29, 20-28.9, 14-19.9, <14 % | Median household income:  <$30000, $30000-$34999, $35000-45999, $46000+ |  |  |
| Niu 2010 [98] | Census tract, USA  (Pop. range 1500-8000)^2^ |  | Poverty rate:  % of population classified as being below the official poverty threshold  <5 %, 5-10 %, 10-20 %, ≥20 % |  |  |
| Nur 2015 [99] | Lower super output area level, England  (Median pop. = 1534) |  |  |  | Index of multiple deprivation (IMD)^b^  quintiles  Q1: least deprived  Q5: most deprived |
| O’Dowd 2015 [100] | Output area, UK  (150 households)^8^ |  |  |  | Townsend Index of multiple deprivation^d^  quintiles  Q1: least deprived  Q5: most deprived |
| Ou 2007 [5] +  Ou 2008 [101] +  Ou 2009 [6] | Census block group, USA  (Median population: 1070)^6^ |  |  |  | Composite measure: median school years, % high school graduates, median income, % living below poverty level, median rent, median house value, % blue-collar job, % > 16 yrs of age in workforce without job; % below 200% of poverty level  quintiles, Q5: highest SES |
| Pagano 2010 [56] | Individual | High: university degree  Intermediate: high school or some secondary education  Low: elementary school  Missing: not declared |  |  |  |
| Pastorino 1990 [57] | Individual |  |  | High: intellectual workers  Mid: craftsmen or skilled manual workers  Low: unskilled manual workers |  |
| Pokhrel 2010 [39] | Individual | Highest attained educational degree or certificate  Basic: lasting typically <10 yrs  Secondary: 10-12 yrs  High: 13 yrs or more |  |  |  |
| Pollock 1997 [102] | Enumeration district, UK  (Pop. range 200-600) |  |  |  | Townsend Index of multiple deprivation^d^  deciles  D1: lowest SES  D10: highest SES |
| Rachet 2008 [103] | Lower super output area level, England  (Median pop. = 1500)^5^ |  | Income domain score of IMD 2004,  quintiles  Q1 most affluent  Q5 most deprived |  |  |
| Rachet 2010 [40] | Lower super output area level, England  (Median pop. = 1500) |  | Income domain score of IMD 2004,  quintiles  Q1 most affluent  Q5 most deprived |  |  |
| Riaz 2011 [104] | Lower super output area level, England  (Median pop. = 1500) |  | Income domain score of IMD 2004,  quintiles  Q1: most affluent  Q5: most deprived |  |  |
| Rich 2011 [105] | Lower super output area level, England  (Median pop. = 1534)^1^ |  |  |  | Townsend Index of multiple deprivation^d^  quintiles  Q1: least deprived  Q5: most deprived |
| Schrijvers 1995a [106] | Postal code, The Netherlands  (8-17 households) |  |  |  | 45 categories of a socioeconomic classification: original classification divided into 5 categories, average number of years of education of main income earners  quintiles  Q1: high SES  Q5: low SES |
| Schrijvers 1995b [107] | Census enumeration district, England  (Average 400 households) |  |  |  | Carstairs index^a^  quintiles  Q1: affluent  Q5: deprived |
| Shack 2007 [108] | Postcode sector  (Pop. = 4660)  Data zone  (Pop. = 770)  Scotland |  |  |  | Carstairs Index^a^  quintiles  1991 (for patients diagnosed 1986-1995)  Index of Multiple Deprivation (IMD)^b^  quintiles  2004 (for patients diagnosed 1996-2000) |
| Shugarman 2008 [109] | Census tract, USA  (Pop. range 1500-8000)^2^ |  | Median income:  <$29000, $29000-$41000, >$41000 |  |  |
| Skyrud 2016 [58] | Individual | Education  Low: <10 yrs  Middle: 10-12 yrs  High: ≥13 yrs | Household income  Low: <20th percentile,  Middle: 20-80th percentile,  High: >80th percentile |  |  |
| Sloggett 2007 [41] | Ward level, England/Wales  Median pop. = 4974) |  |  |  | Registrar General´s Social Class  coded 1–6 for classes I-V respectively  6-point scale: professional to unskilled  Carstairs index^a^  quintiles  Q1: less disadvantaged  Q5: more disadvantaged |
| Smailyte 2016 [42] | Individual | Higher: at least 14 yrs of schooling,  Secondary: 10-13 yrs of schooling,  Lower than secondary: up to 9 yrs of schooling,  (missing included in lowest group) |  |  |  |
| Stanbury 2016 [110] | Local government areas, Australia  (aver. pop = 35954) |  |  |  | Index of education and occupation score  quintiles  Q1: least disadvantaged  Q5: most disadvantaged |
| Sutherland 2008 [111] | Residential area, New Zealand  (Median pop. = 90) |  |  |  | NZDep96 (correspondence with authors):  No access to telephone, people aged 18-59 yrs receiving a means-tested benefit, unemployed aged 18-59 yrs, households below an income threshold, no access to a car, single parent family aged < 60 yrs, no qualifications aged 18-59 yrs, not living in own home, households below bedroom occupancy threshold,  deciles,  D1: least deprived  D10: most deprived |
| Tannenbaum 2014 [112] | Census tract, USA  (Pop. range 1500-8000)^2^ |  | % households in a census tract at or below federal poverty line  grouped into 4 SES categories:  Lowest (≥20%, L)  Middle-low (10-20%, ML)  Middle-high (5-10%, MH)  Highest (<5% H) |  |  |
| Tervonen 2017 [22] | Census collection district  (about 220 dwellings)  Statistical local area  (Median pop. = 21000)  Australia |  |  |  | SEIFA Index of Relative Socio-Economic Disadvantage^c^  quintiles,  Q1: least disadvantaged  Q5: most disadvantaged |
| Vågerö and Persson 1987 [59] | Individual |  |  | White collar workers, blue collar workers, self-employed |  |
| Vercelli 2006 [113] | Country level, Europe |  | Macroeconomic factors:  Gross domestic product,  total health expenditure |  |  |
| Wang 2017a [114] + Wang2017b [115] | County level, USA  (average 90000 residents)^9^ |  | County poverty rate  Percentage of people in the county living below the national poverty threshold in the 2000 U.S. Census  Low-poverty: 10 %  Medium-poverty: 10-19.99 %  High-poverty: 20 % |  |  |
| Wen 2005 [116] | Zip code, USA  (Average pop.: 30000)^7^ |  |  |  | Neighborhood SES:  % residents with household annual income $50000 or over (concentrated affluence),  % households in a neighborhood that were below Federal poverty threshold (concentrated poverty),  % college graduates (aggregate education)  Neighborhood social environment index:  collective efficacy (social cohesion, informal social control), social support, voluntary association, perceived violence |
| Yang 2010 [117] | Census tract, USA  (Pop. range 1500-8000)^2^ |  | % residents in community at or below federal poverty level:  <5%, 5-10%, 10-15%, >15% |  |  |
| Yeole 2004 [61] | Individual | None, <6 yrs, 6-12 yrs, >12 yrs, unknown |  |  |  |
| Yeole 2005 [60] | Individual | None, <6 yrs, 6-12 yrs, >12 yrs, unknown |  |  |  |
| Yim 2012 [62] | Individual |  | Level of premium paid to National Health Insurance  High: 1^st^ quartile  Middle: 2^nd^ to 3^rd^ quartile  Low: 4^th^ quartile |  |  |
| Yu 2008 [119] | Local government area, Australia  (aver. pop = 35954)^10^ |  |  |  | Summary measure of education and occupational levels  quintiles  Q1: most disadvantaged  Q5: least disadvantaged |
| Yu 2014 [118] | Census tract, USA  (Pop. range 1500-8000) |  |  |  | Krieger´s Index:  occupation, unemployed, poverty, income, education, house, ownership, living crowdedness  Yost´s Index:  occupation, unemployed, poverty, income, education, house |
| Zhang-Salomons 2006 [43] | Census tract, USA/Canada  (Mean pop. Detroit=36600, Toronto=4800) |  | Median household income adjusted to household size by an equivalence scale  Poverty: % households living below the Canadian low-income cutoff level in Toronto and % households living below the US poverty line in Detroit |  |  |
