# Supplementary material for: Socioeconomic Differences and Lung Cancer Survival—Systematic Review and Meta-Analysis
Source: Front Oncol. 2018 Nov 27;8:536. doi: 10.3389/fonc.2018.00536 (PMC6277796; doi:10.3389/fonc.2018.00536)
Supplement: Supplementary file 3 [file Table_3.docx]

**Supplement: Table S3.** Association (hazard ratios) of individual measurements of socioeconomic status with survival after lung cancer. ^1^ unless otherwise noted, fully adjusted model; ^2^Multivariable HRs including education were not significant (results not shown in article); Abbreviations: CI = Confidence interval; HR = Hazard ratio; mths = Months; NA = Not available; NSCLC = Non-small cell lung cancer; yrs = Years of age

| **Paper**  **Country** | **Level** | **Hazard Ratio (95 % Confidence Interval)^1^** | |
| --- | --- | --- | --- |
| **Education** |  |  | |
| Europe, north | | | |
| Dalton 2015 [48]  Denmark | Short  Medium  Higher | All-cause survival:  Low stage High stage  1.00 (0.88-1.15) 1.05 (0.98-1.13)  1.03 (0.92-1.16) 1.00 (0.94-1.05)  1.00 1.00 | |
| Kravdal 2000 [55]  Norway | 7-9 yrs  10-12 yrs  13-16 yrs  17+ yrs | All-cause survival: |  |
|  |  | Women | Men |
|  |  | 1.00 | 1.00 |
|  |  | 1.04 (0.96-1.12) | **0.93 (0.89-0.97)** |
|  |  | 1.15 (0.99-1.34) | **0.87 (0.81-0.94)** |
|  |  |  | **0.82 (0.72-0.93)** |
| Berglund 2010 [45]  Sweden | Low  Middle  High | Cause-specific survival:  1.00  0.99 (0.89-1.09)  1.02 (0.87-1.19) | |
| Hussain 2008 [54]  Sweden | <9 yrs  9-11 yrs  12-13 yrs  University graduate  Linear trend | Cause-specific survival:  Women Men  1.00 1.00  0.94 (0.88-1.00) **0.91 (0.86-0.97)**  **0.85 (0.77-0.94) 0.85 (0.80-0.90)**  0.89 (0.78-1.00) **0.87 (0.79-0.96)**  p = 0.0012 p < 0.0001 | |
| Europe, other | | | |
| Di Maio 2012 [50]  Italy | Low  High | All-cause survival:  1.00  **0.85 (0.73-0.99)** | |
| Pagano 2010 [56]  Italy | Low  Intermediate  High  Missing | All-cause survival:  Early stage Advanced stage  1.01 (0.84-1.21) 0.87 (0.73-1.04)  1.12 (0.92-1.37) 0.96 (0.79-1.16)  1.00 1.00  1.15 (0.91-1.46) 1.18 (0.95-1.47) | |
| Aarts 2013 [44]  The Netherlands | Level 1 (low)  Level 2  Level 3  Level 4 (high) | All-cause survival:  0.90 (0.50-1.50)  0.70 (0.40-1.20)  0.80 (0.40-1.40)  1.00 | |
| USA | | | |
| Clement-Duchene 2016 [47]  USA | Some grade school  High school graduate  Some college or more | All-cause survival:  1.00  **1.22 (0.65-2.29)**  **2.00 (1.02-3.93)** | |
| Herndon 2008 [53]  USA | Grades 1-8  Grades 9-11  High school graduate  Some college  College degree | All-cause survival, unadjusted HRs²  1.00  1.04 (0.86-1.26)  1.06 (0.90-1.26)  0.94 (0.78-1.12)  1.00 (0.81-1.23) | |
| Asia | | | |
| Fujino 2007a [51]  Japan | Age at graduation  ≤15  16-18  ≥19 | All-cause survival:  Women Men  1.00 1.00  **0.66 (0.47-0.92)** 0.84 (0.71-1.01)  0.85 (0.49-1.46) **0.73 (0.58-0.93)** | |
| Yeole 2004 [61]  India | None  <6yrs  6-12yrs  >12yrs  Unknown | All-cause survival:  1.00  0.85 (0.69-1.02)  0.97 (0.81-1.15)  0.92 (0.69-1.22)  **1.18 (1.02-1.38)** | |
| Yeole 2005 [60]  India | None  <6yrs  6-12yrs  >12yrs  Unknown | All-cause survival:  1.00  1.05 (0.79-1.37)  1.01 (0.79-1.30)  1.04 (0.71-1.51)  1.21 (0.97-1.50) | |
| **Income** |  |  | |
| Europe, north | | | |
| Dalton 2015 [48]  Denmark | Low  Medium  High | All-cause survival:  Low stage High stage  1.18 (0.99-1.42) **1.12 (1.05-1.19)**  1.14 (0.99-1.32) **1.08 (1.02-1.15)**  1.00 1.00 | |
| Berglund 2010 [45]  Sweden | Low  High | Cause-specific survival:  1.00  **0.89 (0.83-0.97)** | |
| USA | | | |
| Clement-Duchene 2016 [47]  USA | < $20000  $20000-$40000  $40000-$60000  $60000 or more | All-cause survival:  1.00  0.82 (0.45-1.46)  0.71 (0.32-1.57)  0.56 (0.26-1.20) | |
| Greenwald 1994 [34]  USA | Model 1 (only individual)  Income (continuous) | **0.82 (0.71-0.95)** per $5000 (yearly income) | |
| Asia | | | |
| Yim 2012 [62]  Korea | Low  Middle  High | Cause-specific survival:  1.46 (0.99-2.14)  1.11 (0.78-1.57)  1.00 | |
| Chang 2012 [46]  Taiwan | Age <65 yrs  Low  Moderate  High  Age ≥ 65 yrs  Low  Moderate  High | All-cause survival:  Advantaged Neighborhood Disadvantaged Neighborhood  **1.36 (1.10–1.67**) 1.23 (1.00–1.52)  1.13 (0.90-1.42) 1.24 (0.96–1.60)  1.00 0.91 (0.68–1.22)    0.92 (0.67–1.26) 1.03 (0.76–1.41)  1.03 (0.72–1.48) 0.84 (0.55–1.29)  1.00 0.83 (0.51–1.37) | |
| **Occupation** |  |  | |
| Europe, north | | | |
| Berglund 2010 [45]  Sweden | Low  High  Unknown | Cause-specific survival:  1.00  0.93 (0.85-1.01)  1.11 (0.79-1.57) | |
| Kravdal 2000 [55]  Norway | Low education (7-12 yrs)  Manual (exept §)  Non-manual (exept §)  §: hotel and restaurant  workers, ship´s officers,  deck and engine-room  crew  Farmer  Fisherman  No occupation recorded  Medium education (13-16 yrs) (Largely) manual  Non-manual, low level  Non-manual, high level  Teacher  High education (17+ yrs)  Teacher  Physician  Other groups  Medium or high education No occupation recored | Occupation (combined with education), only men  All-cause survival:    1.00  **0.96***        **0.92***  **1.08***  0.97  **1.18***    1.15  0.95  **0.84***  0.93    0.90  0.73  **0.85***    0.68  *p<0.05 | |
| Asia | | | |
| Fujino 2007b [32]  Japan | Type of employment  Employed  Part time  Self-employed  Housewife  Unemployed  Others  Type of jobs (1)  Office work  Manual work  Others  Type of jobs (2)  Sedentary work  Sedentary and standing  Standing position  Moving | All-cause survival:  Women Men    1.00 1.00  1.11 (0.43-2.82) 1.44 (0.91-2.28)  1.83 (0.89-3.76) 1.13 (0.91-1.41)  **2.09 (1.05-4.13)** 1.25 (0.17-8.97)  **2.13 (1.04-4.38)** 1.12 (0.87-1.45)  2.18 (0.94-5.06) 1.17 (0.84-1.62)      1.00 1.00  0.58 (0.32-1.06) 1.07 (0.83-1.36)  0.72 (0.36-1.40) 1.18 (0.82-1.70)      1.00 1.00  0.95 (0.57-1.57) 0.96 (0.74-1.26)  0.39 (0.13-1.11) 0.86 (0.58-1.29)  0.96 (0.62-1.47) 1.06 (0.86-1.31) | |
