# Supplementary material for: Socioeconomic Differences and Lung Cancer Survival—Systematic Review and Meta-Analysis
Source: Front Oncol. 2018 Nov 27;8:536. doi: 10.3389/fonc.2018.00536 (PMC6277796; doi:10.3389/fonc.2018.00536)
Supplement: Supplementary file 4 [file Table_4.docx]

**Supplement: Table S4.** Association (hazard ratios) of aggregated measurements of socioeconomic status with survival after lung cancer. ^1^unless otherwise noted, fully adjusted model; ^2^according to correspondence with author; ^3^upper limit of the confidence interval is 0.995; ^4^approximated from figure in paper;^5^derived from log scale; CD = Census collection district; CI = Confidence interval; CSS = Cause-specific survival; HR = Hazard ratio; NSCLC = Non-small cell lung cancer; RS =Relative survival; SEIFA = Socioeconomic indexes for areas; SES = Socioeconomic status; SLA = Statistical local area

| **Paper**  **Country**  **SES level** | **Level** | **Hazard Ratio (95 % Confidence Interval)^1^** |
| --- | --- | --- |
| **Education** |  |  |
| **USA** | | |
| Johnson 2014 [88]  USA  Census tract | Q4 (low)  Q3  Q2  Q1 (high) | All-cause survival:  Stage I and II Stage III  **1.36 (1.24-1.50) 1.13 (1.02-1.25)**  **1.17 (1.07-1.29) 1.12 (1.02-1.23)**  **1.18 (1.08-1.29) 1.17 (1.07-1.28)**  1.00 1.00 |
| Johnson 2016 [89]  USA  Census tract | Q4 (low)  Q3  Q2  Q1 (high) | All-cause survival:  **1.30 (1.16-1.46)**  **1.13 (1.01-1.26)**  **1.12 (1.01-1.24)**  1.00 |
| Khullar 2015 [90]  USA  Zip code | No high school  ≥29 %  20-28.9 %  14-19.9 %  <14 % | All-cause survival:  **1.11 (1.06-1.16)**  **1.06 (1.02-1.09)**  **1.05 (1.02-1.08)**  1.00 |
| **Income** |  |  |
| Europe | | |
| Berglund 2012 [64]  England  Lower super output area | Q5 (low)  Q4  Q3  Q2  Q1 (high) | All-cause survival:  Early-stage NSCLC Stage III disease Advanced disease/SCLC  1.24 (0.98-1.56) **1.16 (1.01-1.34) 1.12 (1.05-1.20)**  1.15 (0.91-1.46) 1.15 (1.00-1.32) **1.16 (1.08-1.25)**  1.18 (0.93-1.51) 1.14 (0.99-1.32) **1.13 (1.05-1.22)**  1.09 (0.85-1.40) **1.20 (1.03-1.39) 1.17 (1.08-1.26)**  1.00 1.00 1.00  Trend p = 0.11 p = 0.70 p = 0.17 |
| Canada/USA | | |
| Mackillop 1997 [95]  Canada  Postal code | <$20000  $20000-$30000  $30000-$40000  $40000-$50000  >$50000  p Trend | Cause-specific survival:  **1.13 (1.06-1.22)**  **1.14 (1.08-1.21)**  **1.14 (1.08-1.22)**  **1.10 (1.03-1.17)**  1.00  **1.10 (1.07-1.14)** |
| Booth 2010 [66]  Canada  Community | Q1 (low)  Q2  Q3  Q4  Q5 (high) | All-cause survival:  **1.09 (1.02-1.16)**  1.05 (0.98-1.12)  1.03 (0.96-1.10)  0.98 (0.91-1.05)  1.00 |
| Dabbikeh 2017 [73]  Canada  Enumeration/  dissemination area | Constant dollar  (per $10000) | All-cause survival:  1.00 (p = 0.60) |
| Boyd 1999 [67]  Canada/USA  USA: census tract  Canada: enumeration area | Q5 (low)  Q4  Q3  Q2  Q1 (high) | Cause-specific survival:  Canada USA  **1.04 (1.01-1.08) 1.13 (1.10-1.16)**  1.04 (1.00-1.07) **1.05 (1.02-1.08)**  1.01 (0.98-1.05) 1.00 (ref)  0.98 (0.94-1.02) **0.95 (0.93-0.98)**  **0.93 (0.89-0.98) 0.93 (0.90-0.96)**  p < 0.0001 |
| Zhang-Salomons 2006 [43]  Canada/USA  Census tract | Income (quintiles)  Q1 (low)  Q5 (high)  Poverty (quintiles)  Q1 (high)  Q5 (low)  Poverty (tertile)  T1 (high)  T3 (low) | Cause-specific survival:  Canada USA  **1.13 1.39**  1.00 1.00  1.07 **1.38**  1.00 1.00  1.05 **1.29**  1.00 1.00 |
| Khullar 2015 [90]  USA  Zip code | <$30000  $30000-$34999  $35000-45999  $46000+ | All-cause survival:  **1.08 (1.03-1.13)**  **1.07 (1.03-1.11)**  **1.05 (1.02-1.09)**  1.00 |
| McMillan 2017 [96]  USA  Zip code | <$63000  ≥$63000  Unknown | All-cause survival:  1.00  **0.94 (0.89-0.99)**  **1.74 (1.53-1.98)** |
| Greenwald 1994 [34]  USA  Census tract | Model 2  (only census tract)  Median income  Model 3  (both individual and census tract)  Individual income  Median income | All-cause survival:  0.87 (0.65-1.15) per US$5000 increment  **0.82 (0.71-0.95)** per US$5000 increment  1.01 (0.77-1.32) |
| Greenwald 1998 [80]  USA  Census tract | Median income | All-cause survival:  HR = **0.98 (p < 0.0003)** per decile increase |
| Johnson 2014 [88]  USA  Census tract | Q4 (low)  Q3  Q2  Q1 (high) | All-cause survival:  Stage I and II Stage III  1.03 (0.94-1.12) **1.12 (1.03-1.23)**  1.06 (0.97-1.15) **1.10 (1.01-1.20)**  1.08 (1.00-1.17) 1.08 (0.99-1.18)  1.00 1.00  (patients who died within 2 weeks of diagnosis excluded, n = 1889) |
| Johnson 2016 [89]  USA  Census tract | Q4 (low)  Q3  Q2  Q1 (high) | All-cause survival:  1.06 (0.94-1.18)  1.05 (0.96-1.15)  1.04 (0.96-1.13)  1.00 |
| Niu 2010 [98]  USA  Census tract | Poverty level  ≥20 %  10-20 %  5-10 %  <5 % | Cause-specific survival:  Men Women  **1.23 (1.15-1.31) 1.18 (1.09-1.28)**  **1.09 (1.04-1.14) 1.12 (1.06-1.18)**  1.05 (1.00-1.09) 1.04 (0.99-1.09)  1.00 1.00 |
| Shugarman 2008 [109]  USA  Census tract | <$29 000  $29 000-41 000  >$41 000 | All-cause survival:  1.00  0.98  0.95  p < 0.05 |
| Tannenbaum 2014 [112]  USA  Census tract | Low  Middle-low  Middle-high  High | All-cause survival:  1.00  **0.96 (0.94-0.99)**  **0.92 (0.89-0.94)**  **0.87 (0.84-0.91)** |
| Yang 2010 [117]  USA  Census tract | Poverty level  ≥15 %  10-15 %  5-10 %  <5 % | All-cause survival:  **1.05 (1.02-1.09)**  1.03 (1.00-1.06)  1.01 (0.98-1.03)  1.00 |
| Wang 2017a [114]  USA  County | Poverty  Medium-high  Low | Cause-specific survival:  **1.06 (1.06-1.07)**  1.00 |
| Wang 2017b [115]  USA  County | Poverty  Medium-high  Low | Cause-specific survival:  **1.07 (1.06-1.08)**  1.00 |
| Australia | | |
| Bonett 1984 [65]  Australia  Collection district |  | No difference in CSS by income (results not shown in article) |
| **Index** |  |  |
| Europe | | |
| Chouaid 2017 [70]  France  Commune | Q1 (low)  Q2  Q3  Q4 (high)  Q1 (low)  Q2  Q3  Q4 (high) | 1-year all-cause survival  Non-Metastatic Metastatic  disease disease  **1.25 (1.16-1.35) 1.19 (1.13-1.26)**  **1.19 (1.10-1.29) 1.13 (1.07-1.20)**  **1.14 (1.05-1.24) 1.11 (1.04-1.18)**  1.00 1.00  2-year all-cause survival  Non-Metastatic Metastatic  disease disease  **1.21 (1.13-1.30) 1.19 (1.13-1.25)**  **1.15 (1.08-1.23) 1.14 (1.08-1.20)**  **1.10 (1.03-1.18) 1.10 (1.04-1.16)**  1.00 1.00 |
| Aarts 2015 [63]  The Netherlands  Postal code | Low  Intermediate  High  Institutionalized  Unknown | All-cause survival:  1.00  0.90 (0.90-1.00)  0.92 (0.85-0.99)^2^  1.00 (0.80-1.10)  0.90 (0.70-1.00) |
| Louwman 2010 [94]  The Netherlands  Postal code | Lowest SES  Highest SES | All-cause survival:  Men Women  1.11 (1.0-1.2) 1.09 (1.0-1.2)  1.00 1.00 |
| Schrijvers 1995a [106]  The Netherlands  Postal code | Q5 (low)  Q4  Q3  Q2  Q1 (high)  Trend | All-cause survival:  **1.16 (1.03-1.31)**  **1.15 (1.02-1.30)**  1.07 (0.94-1.21)  1.02 (0.87-1.19)  1.00  **1.04 (1.01-1.07)** |
| Iyen-Omofoman 2011 [86]  United Kingdom  Output area | Q5 (low)  Q4  Q3  Q2  Q1 (high)  Missing | All-cause survival, HR unadjusted  1.01 (0.94-1.09)  0.94 (0.88-1.01)  1.03 (0.96-1.10)  0.98 (0.91-1.05)  1.00  **0.78 (0.68-0.88)** |
| Schrijvers 1995b [107]  England  Enumeration district | Q5 (low)  Q4  Q3  Q2  Q1 (high) | All-cause survival:  1.11 (1.00-1.23)  **1.13 (1.04-1.22)**  **1.09 (1.01-1.18)**  1.04 (0.96-1.12)  1.00 |
| Rich 2011 [105]  England  Lower super output area | Q5 (low)  Q4  Q3  Q2  Q1 (high) | All-cause survival:  1.00 (0.95-1.06)  1.03 (0.99-1.06)  1.02 (0.99-1.05)  1.03 (1.00-1.06)  1.00 |
| Canada/USA | | |
| Dabbikeh 2017 [73]  Canada  Enumeration/  dissemination area | Q1 (low)  Q2  Q3  Q4  Q5 (high) | All-cause survival:  1.00 (p = 0.80)  0.97 (p = 0.19)  0.99 (p = 0.70)  0.97 (p = 0.23)  1.00 |
| Gomez 2016 [79]  USA  Census block group | Q1 (low)  Q2  Q3  Q4  Q5 (high) | All-cause survival:  Men Women  1.16 (0.98-1.38) **1.38 (1.10-1.72)**  1.12 (0.95-1.32) **1.22 (1.01-1.47)**  1.11 (0.95-1.28) 1.18 (0.98-1.41)  1.06 (0.93-1.22) 0.97 (0.82-1.15)  1.00 1.00 |
| Hastert 2015 [82]  USA  Census block group | Q1 (low)  Q2  Q3  Q4  Q5 (high)  Trend | Cause-specific survival:  **2.21 (1.69-2.90)**  **2.00 (1.51-2.65)**  **1.64 (1.22-2.19)**  **1.62 (1.21-2.17)**  1.00  p<0.001 |
| Lara 2017 [92]  USA  Census block group | Low SES (Q1-Q3)  High SES (Q4, Q5) | Cause-specific survival:  **1.05 (1.02-1.09)**  1.00 |
| Ou 2007 [5]  USA  Census block group | Q1 (low)  Q2  Q3  Q4  Q5 (high) | All-cause survival:  Stage IA Stage IB  1.00 1.00  0.91 (0.81-1.03) **0.91 (0.82-1.00^3^)**  **0.87 (0.78-0.98) 0.90 (0.81-0.98)**  **0.76 (0.68-0.86) 0.86 (0.78-0.94)**  **0.79 (0.70-0.89) 0.75 (0.68-0.82)** |
| Ou 2008 [101]  USA  Census block group | Q1 (low)  Q2  Q3  Q4  Q5 (high) | All-cause survival:  1.00  **0.91 (0.85-0.98)**  **0.90 (0.84-0.97)**  **0.83 (0.77-0.89)**  **0.78 (0.72-0.84)** |
| Ou 2009 [6]  USA  Census block group |  | All-cause survival:  **0.97** **( 0.94-0.99)** (increase per SES score)  p (trend) = 0.01 |
| Lara 2014 [93]  USA  Census tract | Lowest SES  Mid SES  Highest SES | Cause-specific survival:  1.00  **0.96 (0.94-0.98)**  **0.90 (0.89-0.92)** |
| Wen 2005^4^ [116]  USA  Zip code | Social index | Cause-specific survival:  1.02 (0.94-1.10)^5^ |
| Australia/New Zealand | | |
| Hall 2004 [81]  Australia  Collection district | Q5 (low)  Q4  Q3  Q2  Q1 (high) | All-cause survival:  1.05 (0.93-1.20)  1.03 (0.92-1.15)  1.09 (0.98-1.20)  1.03 (0.92-1.16)  1.00 |
| Tervonen 2017 [22]  Australia  Collection district and Statistical local area | Q5 (low)  Q4  Q3  Q2  Q1 (high) | SHR (95 % CI) full model, all-cause survival  SLA CD  **1.14 (1.08-1.19) 1.21 (1.15-1.27)**  **1.15 (1.09-1.21) 1.13 (1.08-1.19)**  **1.13 (1.08-1.19) 1.10 (1.05-1.16)**  1.06 (1.00-1.11) **1.06 (1.01-1.12)**  1.00 1.00 |
| Currow 2014 [72]  Australia  Postal code area | Q5 (low)  Q4  Q3  Q2  Q1 (high) | SHR (CSS, 95 % CI) full model, all-cause survival  1.24 (0.97-1.59)  1.19 (0.94-1.52)  1.00 (0.79-1.27)  1.02 (0.82-1.28)  1.00 |
| Denton 2017 [74]  Australia  Postal code area | Q1 (low)  Q2  Q3  Q4  Q5 (high) | All-cause survival:  1.10 (0.89-1.30)  1.00 (0.84-1.20)  1.00 (0.83-1.20)  1.00 (0.85-1.20)  1.00 |
| Haynes 2008 [83]  New Zealand  Census area unit | Low  Medium  High  Highest | All-cause survival:  1.00  1.06  **1.11** (p<0.05)  **1.21** (p<0.01) |
| Asia | | |
| Kwak 2017a [91]  Korea  Dong | Q4 (low)  Q3  Q2  Q1 (high) | All-cause survival:  1.06 (0.87–1.30)  1.18 (1.00–1.40)  1.01 (0.87–1.17)  1.00 |
| Kwak 2017b [37]  Korea  Dong | Q4 (low)  Q3  Q2  Q1 (high) | Cause-specific survival:  **1.08 (1.01-1.15)**  **1.11 (1.05-1.17)**  **1.08 (1.03-1.13)**  1.00 |
