# Supplementary material for: Socioeconomic Differences and Lung Cancer Survival—Systematic Review and Meta-Analysis
Source: Front Oncol. 2018 Nov 27;8:536. doi: 10.3389/fonc.2018.00536 (PMC6277796; doi:10.3389/fonc.2018.00536)
Supplement: Supplementary file 5 [file Table_5.docx]

**Supplement: Table S5.** Survival after lung cancer stratified by individual measurements of socioeconomic status. ^1^No numbers reported, but approximated from figure for selected papers and survival rates; ^2^according to correspondence with author; Adv. N. = Advantaged neighborhood; CI = Confidence interval; CSS = Cause-specific survival; Disadv. N. = Disadvantaged neighborhood; HR = Hazard ratio; KM = Kaplan-Meier curves; NA = Not available; OR = Odds ratio; OS = Overall survival; RER = Relative excess risk; SE = Standard error; Yrs = Years of age

| **Paper**  **Country** | **Level** | **Survival** | | | | |
| --- | --- | --- | --- | --- | --- | --- |
|  |  | **Median (months, % (95% CI)** | **1-year  (%, 95% CI)** | **3-year  (%, 95% CI)** | **5-year  (%, 95% CI)** | **Other** |
| **Education** |  |  |  |  |  |  |
| Europe, north | | | | | | |
| Dalton 2008 [49]  Denmark | Basic/high school  Vocational education  Higher education  Unknown | KM | Relative survival:  Men Women  28 (27-30) 33 (32-34)  30 (29-32) 36 (34-38)  34 (32-37) 34 (30-37)  28 (22-35) 37 (28-47) | KM | Relative survival:  Men Women  7 (6-8) 9 (8-10)  8 (7-8) 9 (8-10)  10 (8-12) 10 (8-12)  7 (4-12) 10 (6-18) |  |
| Pokhrel 2010 [39]  Finland | Basic (<10yrs)  Secondary (10-12 yrs)  High (≥13 yrs |  |  |  | Cause-specific survival:  Men Women  9.2 12.3  9.5 13.5  10.6 18.8 |  |
| Skyrud 2016 [58]  Norway | low: <10 yrs  middle: 10-12 yrs  high: ≥13 yrs |  |  |  |  | RER (95 % CI), RS  1.00  **0.95 (0.92-0.98)**  **0.89 (0.84-0.95)** |
| Berglund 2010 [45]  Sweden | Low  Middle  High |  | Cause-specific survival:  39  44  50 | Cause-specific survival:  18  23  25 |  |  |
| Europe, other | | | | | | |
| Di Maio 2012 [50]  Italy | Low  High | Overall survival:  7.6 (7.2-8.1)  9.4 (8.2-11.1) | KM | KM |  |  |
| Smailyte 2016 [42]  Lithuania | Lower than secondary (≤9 yrs)  Secondary (10-13 yrs)  Higher (≥14 yrs) |  |  |  | RS % (SE)  Men Women  7.7 (0.5) 11.2 (1.7)  11.3 (0.8) 14.1 (2.0)  11.6 (1.8) 23.3 (4.3) |  |
| Aarts 2013^2^ [44]  The Netherlands | Level 1 (low)  Level 2  Level 3  Level 4 (high) | KM | Overall survival:  44  50  50  35 | Overall survival:  23  32  35  20 | KM | 50 months OS (%):  20  24  20  20 |
| USA | | | | | | |
| Herndon 2008 [53]  USA | Grades 1-8  Grades 9-11  High school grade  Some college  College degree | Overall survival:  11.3 (9.7-13.9)  11.0 (9.8-12.9)  10.5 (9.5-11.4)  11.3 (10.3-12.6)  11.1 (10.0-13.1) | KM | KM | KM |  |
| Asia | | | | | | |
| Yeole 2004 [61]  India | None  <6 yrs  6-12 yrs  >12 yrs  Unknown |  |  |  | Overall survival:  8.5  8.9  4.6  6.9  4.6 |  |
| Yeole 2005 [60]  India | None  <6yrs  6-12yrs  >12yrs  Unknown |  |  |  | Overall survival:  13.1  18.8  14.1  9.3  10.9 |  |
| **Income** |  |  |  |  |  |  |
| Europe, north | | | | | | |
| Dalton 2008 [49]  Denmark | Low  Medium  High |  | Relative survival:  Men Women  27 (26-29) 32 (30-34)  30 (29-32) 33 (32-35)  33 (31-36) 36 (33-39) |  | Relative survival:  Men Women  7 (6-7) 9 (8-10)  8 (7-9) 8 (8-9)  8 (7-9) 10 (8-12) |  |
| Skyrud 2016 [58]  Norway | Low: <20th percentile  Middle: 20-80th perc.  High: >80th percentile |  |  |  |  | RER (95 % CI), RS  1.00  **0.90 (0.86-0.94)**  **0.84 (0.79-0.90)** |
| Berglund 2010 [45]  Sweden | Low  High |  | Overall survival:  38  45 | Overall survival:  18  22 |  |  |
| USA | | | | | | |
| Chirikos 1984 [30]  USA | Low  High | KM | Stage (CSS^1^)  localized regional  /distant  65 44  74 39 | Stage (CSS^1^)  localized regional  /distant  33 12  44 10 |  |  |
| Asia | | | | | | |
| Yim 2012 [62]  Korea | Low: 4^th^ quartile  Middle: 2^nd^- 3^rd^ quartile  High: 1^st^ quartile | KM | KM | Overall survival^1^:  25.0  24.5  31.3 |  |  |
| Chang 2012 [46]  Taiwan | Low  Moderate  High  Low  Moderate  High | KM | KM | KM | Overall survival:  Adv. N. Disadv. N.  Age <65 yrs  20.9 22.5  25.4 25.8  29.4 35.3  Age ≥ 65 yrs  20.2 17.0  18.1 28.3  23.6 21.9 |  |
| **Occupation** |  |  |  |  |  |  |
| Europe, north | | | | | | |
| Dalton 2008 [49]  Denmark | *Occupation 1*  Working  Unemployed  Early retirement  *Occupation 2*  creative core  creative professionals  bohemians  service  manual  agricultural  unknown |  | Relative survival:  Men Women  37 (35-38) 41 (39-42)  29 (27-32) 38 (35-41)  23 (21-26) 35 (33-38)  29 (24-35) 36 (26-52)  31 (29-34) 37 (32-42)  30 (20-45) 24 (24-42)  31 (29-33) 35 (33-36)  29 (28-31) 34 (32-37)  30 (26-34) 29 (21-41)  27 (24-30) 31 (29-33) |  | Relative survival:  Men Women  10 (9-11) 11 (11-12)  8 (6-10) 11 (10-13)  8 (7-10) 10 (8-12)  8 (6-12) 14 (7-30)  8 (7-10) 9 (7-12)  10 (5-18) NA  8 (7-9) 9 (8-10)  7 (7-8) 9 (7-11)  9 (7-13) 5 (2-14)  6 (5-8) 8 (7-10) |  |
| Berglund 2010 [45]  Sweden | Low  High  Unknown |  | Overall survival:  41  46  34 | Overall survival:  20  22  17 |  |  |
| Vågerö and Persson 1987 [59]  Sweden | White collar workers  Blue collar workers  Self-employed farmers | KM | KM | KM | Relative survival^1^  (only men):  12  10  13 |  |
| Europe, other | | | | | | |
| Sloggett 2007 [41]  England/Wales | Social class (per group) |  |  |  |  | RER (95 % CI), RS  **1.04 (1.01–1.08)** |
| Grivaux 2011 [52]  France | Farmer  Manager  Service provider  Self-employed  Unemployed  Employee  Manual worker |  |  |  | Overall survival:  6.4  12.0  10.8  11.4  7.5  11.8  10.4 |  |
| Pastorino 1990 [57]  Italy | Low social class  Mid social class  High social class |  |  |  | Overall survival:  11  3  NA |  |
