# Supplementary material for: Socioeconomic Differences and Lung Cancer Survival—Systematic Review and Meta-Analysis
Source: Front Oncol. 2018 Nov 27;8:536. doi: 10.3389/fonc.2018.00536 (PMC6277796; doi:10.3389/fonc.2018.00536)
Supplement: Supplementary file 6 [file Table_6.docx]

**Supplement: Table S6.** Survival after lung cancer stratified by aggregated measurements of socioeconomic status. ^1^No numbers reported, but approximated from figure(s) for selected papers and survival rates; ^2^Fitted deprivation gap in survival between most affluent and most deprived groups was estimated using variance-weighted linear regression; ^3^according to correspondence with author; ^4^Conditional on 3 months RS; ^5^Conditional on 1-year RS; ^6^Calculated from information on patients that died within 1-30 and 31-90 days; ^7^Calculated from % deceased at 5 years; Abbreviations: CI = Confidence interval; CSS = Cause-specific survival; F.-up = Follow-up length; GDP = Gross domestic product; HR = Hazard ratio; KM = Kaplan-Meier curves; mths = Months; NA = Not available; NL = The Netherlands; NSCLC = Non-small cell lung cancer; OR = Odds ratio; OS = Overall survival; QS = Quality score; RS =Relative survival; SEER = Surveillance, Epidemiology, and End Results Program; SEIFA = Socioeconomic indexes for areas; SES = Socioeconomic status; Yrs = Years of age

| **Paper**  **Country**  **SES level** | **Level** | **Survival** | | | | |
| --- | --- | --- | --- | --- | --- | --- |
|  |  | **Median**  **(months)** | **1-year  (%, 95% CI)** | **3-year**  **(%, 95% CI)** | **5-year**  **(%, 95% CI)** | **Other** |
| **Education** |  |  |  |  |  |  |
| USA | | | | | | |
| Erhunmwunsee 2012^1^ [76]  USA  Census tract | Education 1  Q1 (low)  Q3 (high)  Education 2  Q1 (low)  Q3 (high) | CSS:  14.03  16.20  14.27  15.50 | CSS:  54  59  55  58 | CSS:  26  31  29  31 | CSS:  17  21  18  20 | KM |
| Khullar 2015 [90]  USA  Zip code | No high school  ≥29 %  20-28.9 %  14-19.9 %  <14 % | KM | Overall survival:  87.7 (87.0-88.4)  89.0 (88.4-89.5)  89.4 (88.9-90.0)  90.4 (90.0-90.9) | Overall survival:  70.1 (69.1-71.1)  72.0 (71.2-72.8)  72.6 (71.8-73.4)  75.2 (74.5-75.8) | Overall survival:  55.9 (54.8-57.0)  58.5 (57.6-59.4)  59.4 (58.5-60.3)  63.1 (62.3-63.8) |  |
| Melvan 2015 [97]  USA  Zip code | No high school  ≥29 %  20-28.9 %  14-19.9 %  <14 % |  |  |  |  | 30-day OS (%):  96.56  96.79  96.98  97.39  p < 0.001 |
| **Income** |  |  |  |  |  |  |
| Europe | | | | | | |
| Berglund 2012 [64]  England  Lower super output area | Q5 (low)  Q1 (high) | KM | KM | Overall survival:  39  50  (early stage NSCLC) | KM |  |
| Rachet 2010 [40]  England/Wales  Lower super output area | Most affluent  Most affluent |  | Men Women  Year 1996 (RS)  24.8 24.7  Year 2006 (RS)  27.4 30.9 |  |  | Deprivation gap^2^ (%):  Year Men Women  1996 **-3.3** -1.5  2006 -1.6 **-3.1** |
| Forrest 2015 [78]  United Kingdom  Lower super output area | Q5 (low)  Q4  Q3  Q2  Q1 (high) |  |  |  |  | 2-year OS (%):  14.4  15.1  15.3  15.7  17.6 |
| Rachet 2008 [103]  England/Wales  Lower super output area |  |  |  |  | Figure 2 (5-year relative survival) by gender and period, no difference by deprivation | Deprivation gap^2^ (95 % CI) 1991-1995:  1-year RS  Men **-1.1 (-1.9- -0.3)**  Women **-1.9 (-3.0- -0.7)**  5-year RS  Men -0.5 (-1.0-0.0)  Women **-1.2 (-1.9- -0.6)**  10-year RS  Men -0.4 (-0.9-0.2)  Women **-1.5 (-2.2- -0.8)** |
| Riaz 2011 [104]  England  Lower super output area | Q5 (low)  Q4  Q3  Q2  Q1 (high)  Q5 (low)  Q4  Q3  Q2  Q1 (high)  Q5 (low)  Q4  Q3  Q2  Q1 (high)  Q5 (low)  Q4  Q3  Q2  Q1 (high) |  | Overall survival:  Men  Urban  26 (25-26)  26 (25-26)  25 (25-26)  26 (25-27)  27 (26-28)  Rural  25 (21-28)  25 (23-27)  25 (24-26)  26 (25-28)  29 (28-31)  Women  Urban  28 (27-29)  28 (27-29)  29 (28-30)  29 (28-30)  31 (30-32)  Rural  27 (23-31)  26 (24-28)  28 (26-30)  29 (28-31)  32 (30-34) |  |  |  |
| Vercelli 2006 [113]  Europe  Country level | Country  (GDP rank order; 1=high, 19=low)  Switzerland (1)  Germany (2)  France (3)  Denmark (4)  Norway (5)  NL (6)  Austria (7)  Iceland (8)  Sweden (9)  Italy (10)  Finland (11)  UK (12)  Spain (13)  Portugal (14)  Slovenia (15)  Czeck Republic (16)  Slovakia (17)  Poland (18)  Estonia (19) |  |  |  | Relative survival:  Men Women  8 13  10 9  14 16  6 5  7 9  10 10  10 16  8 9  7 10  9 8  7 9  6 6  12 10  NA NA  7 8  4 7  6 10  6 6  6 11 |  |
| Evans and Pritchard 2000 [77]  Europe/USA  Country level | Country Rank order  (GDP)  USA 1  France 2  Germany 3  NL 4  Switzerland 5  Italy 6  Finland 7  Denmark 8  England 9  Spain 10 |  |  |  | Rank order (RS)  Men Women  1 (11.8) 1 (15.7)  5 (8.7) 5 (13.0)  6 (8.1) 4 (10.0)  3 (10.5) 2 (NA)  1 (11.8) NA (12.3)  8 (6.0) NA (11.4)  4 (9.1) 6 (10.3)  7 (6.2) 7 (6.4)  10 (3.8) 8 (3.5)  9 (5.0) 3 (NA) |  |
| Canada/USA | | | | | | |
| Mackillop 1997 [95]  Canada  Postal code | <$20000  $20000-$30000  $30000-$40000  $40000-$50000  >$50000  (Canadian $) | KM | KM | KM | Cause-specific survival:  11  11  12  13  15 |  |
| Booth 2010 [66]  Canada  Community | Q1 (low)  Q2  Q3  Q4  Q5 (high) |  |  | CSS:  25.8 (24.1-27.6)  26.1 (24.5-27.8)  27.4 (25.6-29.3)  27.3 (25.3-29.3)  27.9 (25.7-30.1) | OS:  15.4 (14.2-16.8)  16.5 (15.2-17.8)  16.4 (15.0-17.8)  17.3 (15.8-18.9)  18.6 (16.9-20.4) |  |
| Boyd 1999 [67]  Canada/USA  USA: Census tract  Canada: Enumer-  ation area | Q1 (low)  Q2  Q3  Q4  Q5 (high) | KM | KM | KM | Canada USA (both CSS)  15.3 12.0  15.4 14.3  16.5 15.6  16.8 17.0  18.5 17.8 |  |
| Gorey 1997 [33]  Canada/USA  Census tract | Low  Middle  High  Low  Middle  High  Low  Middle  High  Low  Middle  High |  | Overall survival:  Canada  Men Women  35.4 40.7  35.9 39.6  36.8 39.1  USA  Men Women  29.7 34.1  33.9 39.6  36.4 43.8 |  | Overall survival:  Canada  Men Women  13.2 15.0  14.2 17.6  11.1 18.4  USA  Men Women  6.7 9.5  8.8 10.4  11.4 15.7 | Survival rate ratios (SRR)  Canada: (1-year survival)  Men Women  0.96 (0.88-1.05) 1.04 (0.94-1.15)  0.98 (0.91-1.05) 1.01 (0.93-1.10)  1.00 1.00  5-year survival  Men Women  1.19 (0.91-1.55) 0.82 (0.61-1.10)  1.28 (0.98-1.67) 0.96 (0.76-1.21)  1.00 1.00  USA: (1-year survival)  Men Women  **0.82 (0.76-0.88) 0.78 (0.71-0.85)**  0.93 (0.86-1.01) **0.90 (0.82-0.98)**  1.00 1.00  5-year survival  Men Women  **0.59 (0.45-0.78) 0.61 (0.43-0.86)**  0.77 (0.55-1.08) 0.66 (0.42-1.03)  1.00 1.00 |
| Zhang-Salomons 2006^1^ [43]  Canada/USA  Census tract | Q5 (low)  Q1 (high) |  |  |  | Canada USA (both CSS)  15 11  18 20 |  |
| Caposole 2014 [68]  USA  Census tract | SES4 (low)  SES3  SES2  SES1 (high) | Overall survival:  9.00 (6.98-11.03)  11.00 (9.99-12.01)  12.00 (10.50-13.50)  12.00 (10.06-13.94) |  |  |  |  |
| Erhunmwunsee 2012^1^ [76]  USA  Census tract | Income 1  Q3 (low)  Q1 (high)  Income 2  Q3 (low)  Q1 (high) | CSS:  13.30  16.70  13.87  16.40 | CSS:  53  59  57  63 | CSS:  28  31  29  32 | CSS:  19  20  19  21 | KM |
| Greenwald 1998 [80]  USA  Census tract | D1 (low)  D2  D3  D4  D5  D6  D7  D8  D9  D10 (high) |  |  |  | Overall survival:  22.4  25.0  32.3  30.9  33.3  34.1  30.4  37.4  32.2  45.3 |  |
| Lipworth 1970 [38]  USA  Census tract | (US $)  < $5000  >$5000 |  | Relative survival:  Men Women  18.1 20.5  21.2 29.2 | Relative survival:  Men Women  9.6 5.4  10.0 15.3 |  | 3-year relative survival conditional on 1-year survival:  Men Women  53.4 26.3  47.1 52.4 |
| Niu 2010 [98]  USA  Census tract | Poverty level  >15%  10-15%  5-10%  <5% |  |  |  | Cause-specific survival:  1986-91/1993-99:  Men Women  8.8/10.3 13.7/15.3  12.6/12.8 13.7/15.6  11.0/13.7 15.9/18.0  14.2/15.2 18.6/19.5 |  |
| Tannenbaum 2014 [112]  USA  Census tract | Low  Middle-low  Middle-high  High | Overall survival:  8.1  9.6  10.9  12.1 | Overall survival:  40.3  44.2  47.5  50.0 | Overall survival:  17.5  21.2  24.7  26.9 | Overall survival:  11.4  14.5  16.9  19.4 | KM |
| Yang 2010 [117]  USA  Census tract | Below poverty level  >15%  10-15%  5-10%  <5% | Overall survival:  7.5  8.3  9.1  9.3 | KM | KM | KM |  |
| Khullar 2015 [90]  USA  Zip code | <$30000  $30000-$34999  $35000-$45999  >$46000  (US $) | KM | Overall survival:  87.5 (86.7-88.3)  88.5 (87.8-89.1)  89.1 (88.6-89.6)  90.7 (90.3-91.1) | Overall survival:  69.7 (68.6-70.7)  70.7 (69.8-71.6)  72.7 (72.0-73.4)  75.4 (74.8-76.0) | Overall survival:  55.5 (54.2-56.7)  56.9 (55.9-57.9)  59.6 (58.7-60.4)  63.2 (62.5-63.9) |  |
| Melvan 2015 [97]  USA  Zip code | <$30000  $30000-$34999  $35000-$45999  >$46000  (US $) |  |  |  |  | 30-day OS (%):  96.51  96.69  96.94  97.38 |
| Wang 2017a [114]  USA  County | RS (% ± SE)  high poverty  medium poverty  Low poverty  high poverty  medium poverty  Low poverty  high poverty  medium poverty  Low poverty |  | 1983-1992  33.7±1.2  38.4±0.2  41.0±0.2  1993-2002  37.0±0.5  40.1±0.2  42.5±0.2  2003-2012  40.5±0.3  44.8±0.1  48.0±0.2 |  |  |  |
| Wang 2017b [115]  USA  County | high poverty  medium poverty  Low poverty  high poverty  medium poverty  Low poverty  high poverty  medium poverty  Low poverty |  | RS (% ± SE)  1983-1992  28.4±2.4  31.6±0.5  34.1±0.5  1993-2002  30.8±1.0  32.1±0.4  35.1±0.4  2003-2012  31.3±0.6  32.5±0.3  34.9±0.4 | RS (% ± SE)  1983-1992  4.7±1.2  6.4±0.3  7.9±0.3  1993-2002  6.8±0.6  8.2±0.2  9.5±0.3  2003-2012  8.7±0.4  8.9±0.2  10.1±0.2 | RS (% ± SE)  1983-1992  1.8±0.7  4.3±0.2  5.5±0.2  1993-2002  4.2±0.4  5.6±0.2  6.6±0.2  2003-2012  5.7±0.3  6.2±0.2  6.9±0.2 | 2-year RS (% ± SE):  1983-1992  8.1±1.5  10.5±0.3  12.2±0.3  1993-2002  11.4±0.7  12.7±0.3  14.4±0.3  2003-2012  13.7±0.5  13.6±0.2  15.4±0.3 |
| Australia | | | | | | |
| Bonett 1984 [65]  Australia  Collection district |  |  |  |  |  | No difference in CSS by income (results not shown in article) |
| **Occupation** |  |  |  |  |  |  |
| Cheyne 2013 [69]  United Kingdom  Lower super output area | Wealthy achievers  Urban professional  Comfortably off  Moderate means  Hard pressed | Median OS (days):  212  289  196  207  235 | Overall survival:  32  41  36  35  39 |  |  |  |
| **Index** |  |  |  |  |  |  |
| Europe | | | | | | |
| Jansen 2014 [35]  Germany  District level | Q5 (low)  Q4  Q3  Q2  Q1 (high) |  |  |  | RS (% ± SE)  14.4 ± 0.4  16.5 ± 0.4  16.1 ± 0.4  17.8 ± 0.5  18.0 ± 0.5 | RER (95 % CI) full model (Q5 vs. Q1-Q4)  3-months RS 5-year RS  **1.21 (1.12-1.30) 1.07 (1.04-1.09)**  cond. 1-year RS^4^ cond. 5-year RS^5^  **1.16 (1.10-1.21) 1.10 (1.04-1.16)** |
| Aarts 2015 [63]  The Netherlands  Postal code | Low  Intermediate  High  Institutionalized  Unknown | Overall survival:  16  20  23  12  24 | Overall survival:  16  21  23  13  28 |  |  | 6-months overall survival (%):  36  41  46  26  45 |
| Louwman 2010 [94]  The Netherlands  Postal code | Low SES  Inter-mediate SES  High SES  Low SES  Inter-mediate SES  High SES |  | Overall survival:  Men  36  39  41  Women  41  42  46 |  |  |  |
| Schrijvers 1995a [106]  The Netherlands  Postal code | Q5 (low)  Q4  Q3  Q2  Q1 (high) |  |  |  | Relative survival:  11 (9-13)  12 (9-15)  14 (11-17)  17 (13-21)  15 (12-18) |  |
| Pollock 1997 [102]  England  Enumeration district | D1 (low)  D2  D3  D4  D5  D6  D7  D8  D9  D10 (high) | KM | KM | KM | Relative survival:  7 (5-9)  7 (5-9)  5 (3-6)  7 (6-9)  5 (4-7)  6 (5-8)  6 (5-8)  6 (5-8)  5 (4-6)  6 (5-7) |  |
| Schrijvers 1995b [107]  England  Enumeration district | Q5 (low)  Q4  Q3  Q2  Q1 (high) |  |  |  | Relative survival:  6.5  6.3  7.2  7.3  8.0 |  |
| Nur 2015 [99]  England  Lower super output area | Q5 (low)  Q4  Q3  Q2  Q1 (high)  Q5 (low)  Q4  Q3  Q2  Q1 (high) | KM | Reported for different age groups: here only ranges.  Net survival:  Men (range)  19.4-33.8  18.9-42.4  19.3-45.7  19.2-37.5  20.7-47.2  Women (range)  19.6-39.8  19.7-49.3  21.2-50.9  20.2-50.0  22.4-53.1 | KM | KM |  |
| Coleman 2001 [71]  England/Wales  Enumeration district | Affluent |  |  |  | Relative survival  6.0 | Deprivation gap^2^  -1.0 |
| Sloggett 2007 [41]  England/Wales  Ward | Carstairs score |  |  |  |  | RER (95 % CI), relative survival  **1.06 (1.03–1.09)** |
| Coleman 2004 [31]  England/Wales  Electoral ward |  |  |  |  |  | Deprivation gap³, relative survival  Men **-1.4 (2.2 – -0.7)**  Women -0.6 (-1.6 – 0.3) |
| Campbell 2000 [29]  Scotland  Output area | Q5 (most deprived)  Q4  Q3  Q2  Q1 (least deprived) |  | Overall survival:  21.2  20.6  21.2  23.5  24.0 |  |  |  |
| Shack 2007 [108]  Scotland  Postcode sector |  |  |  |  |  | Deprivation gap^2^ (5 year RS)  Men **-1.6 (-3.1- -0.1)**  Women -1.5 (-3.3-0.4) |
| Iyen-Omofoman 2011 [86]  United Kingdom  Output area | Q5 (low)  Q4  Q3  Q2  Q1 (high)  Missing | Median OS (days, IQR):  221 (72-608)  242 (76-666)  224 (67-587)  232 (79-640)  223 (78-593)  296 (116-1032) | 37  39  36  36  37  44 |  | 10  12  9.9  10  9.7  18 | 6-mths OS:  55  58  56  57  56  64 |
| O’Dowd 2015 [100]  United Kingdom  Output area | Q5 (low)  Q4  Q3  Q2  Q1 (high)  Missing |  |  |  |  | OS (%): 1-month^6^ 3-month^6^  88.71 72.51  89.60 73.84  85.30 63.70  89.22 74.10  89.47 74.26  92.55 79.38 |
| Cheyne 2013 [69]  United Kingdom  Lower super output area | Q5 (low)  Q4  Q3  Q2  Q1 (high) | Median OS (days):  192  220  233  217  238 | OS:  33  38  40  37  39 |  |  |  |
| Ellis 2014 [75]  United Kingdom  Lower super output area | Q5 (low)  Q4  Q3  Q2  Q1 (high) (Deprivation-specific life tables) |  | Relative survival:  Men Women  27.1 28.7  27.4 27.7  28.0 30.2  27.4 29.6  29.1 32.2  Deprivation gap^2^  -1.5 -3.3 |  | Relative survival:  Men Women  15.1 15.7  14.5 15.3  15.3 16.5  14.9 17.0  16.0 17.8  Deprivation gap^2^  -0.8 -2.2 |  |
| Jack 2006 [87]  United Kingdom  Ward level | Q5 (low)  Q4  Q3  Q2  Q1 (high)  Not known |  | Overall survival:  21  22  25  33  29  20 |  |  |  |
| Canada/USA | | | | | | |
| Dabbikeh 2017^1^ [73]  Canada  Enumeration/dis-semination area | Q1 (low)  Q2  Q3  Q4  Q5 (high) |  |  |  | Cause-specific survival:  Year of diagnosis 2007  21.0  22.5  22.0  24.0  25.0 |  |
| Gomez 2016 [79]  USA  Census block group | Q1 (low)  Q2  Q3  Q4  Q5 (high)  Q1 (low)  Q2  Q3  Q4  Q5 (high) | Overall survival:  Men  10.4 (8.3- 13.3)  11.0 (8.9- 13.2)  13.3 (11.2- 16.9)  13.9 (11.7-16.2)  15.2 (12.8- 17.9)  Women  10.9 (8.4- 15.1)  17.3 (13.1- 21.7)  17.3 (13.5- 23.4)  19.8 (16.7- 22.9)  23.0 (19.7- 26.7) |  |  |  |  |
| Ou 2008 [101]  USA  Census block group | Q1 (low)  Q2  Q3  Q4  Q5 (high) | Overall survival:  39  48  50  58  65 | KM | KM | Overall survival:  38.5  42.8  44.6  49.0  52.3 |  |
| Ou 2009 [6]  USA  Census block group | Q1 (low)  Q2  Q3  Q4  Q5 (high) | Overall survival:  4  5  5  6  7 | Overall survival:  19.2  18.7  17.8  17.7  28.0 |  |  | 2-year overall survival (%):  7.0  5.0  5.8  5.1  6.2 |
| Lara 2014 [93]  USA  Census tract | Lowest SES  Mid SES  Highest SES | CSS:  11 (10-12)  12 (11-14)  16 (15-17) |  |  |  |  |
| Yu 2014 [118]  USA  Census tract |  |  |  |  | CSS, Figure 5, no numbers reported |  |
| Australia/New Zealand | | | | | | |
| Hall 2004 [81]  Australia  Collection district | Q1 (low)  Q2  Q3 Q4 Q5 (high) |  |  |  | Overall survival^7^:  10.4  12.3  14.0  13.6  12.3 |  |
| Denton 2017 [74]  Australia  Postal code area | Low SES  High SES  Low SES  High SES |  |  |  | Overall survival:  Stage I Stage II  55 45  58 37  Stage III Stage IV  23 9.6  15 9.4 |  |
| Hui 2005^1^ [84]  Australia  Postal code area | Q1 (low)  Q2  Q3 Q4 Q5 (high) | No significant difference in median or overall survival (p = 0.20) | KM | Overall survival:  8  14  13  18  14 |  |  |
| Stanbury 2016 [110]  Australia  Local government area | Q5 (low)  Q4  Q3  Q2  Q1 (high)  Q5 (low)  Q4  Q3  Q2  Q1 (high) |  |  |  | Relative survival:  1996-2000  14.5  15.0  14.5  16.4  16.2  2004-2008  14.2  17.0  16.3  17.2  18.1 | RER (95 % CI), relative survival  1996-2000  **1.17 (1.10-1.25)**  **1.12 (1.05-1.20)**  **1.12 (1.05-1.20)**  **1.12 (1.05-1.19)**  1.00  2004-2008  **1.26 (1.19-1.34)**  **1.19 (1.12-1.27)**  **1.18 (1.11-1.26)**  **1.12 (1.05-1.19)**  1.00 |
| Yu 2008 [119]  Australia  Local government area | Q1 (low)  Q2  Q3  Q4  Q5 (high)  Q1 (low)  Q2  Q3  Q4  Q5 (high) |  |  |  | Relative survival:  Men  12.2  10.7  11.5  15.3  14.0  Women  16.9  14.6  16.0  17.0  17.1 | RER, relative survival  1.18  1.16  1.21  1.07  1.00 |
| Jeffreys 2009 [36]  New Zealand  Meshblock | D9-10  (most deprived)  D7-8  D5-6  D1-4  (least deprived) |  |  |  | Relative survival:  8 (7-9)  8 (7-9)  10 (9-11)  13 (11-14) | Deprivation gap^2^ (95 % CI)  -0.07 (-0.14-0.00) |
| Sutherland 2008^3^ [111]  New Zealand  Meshblock | Depscore  D8-D10 (low)  D4-D7  D1-D3 (high) |  | Overall survival:  23  23  33 |  | Overall survival:  4  15  7 | Overall survival:  6 weeks 3 mths 6 mths 2 yrs (%)  75 62 45 11  85 54 46 15  87 73 53 13 |
| Asia | | | | | | |
| Ito 2014 [85]  Japan  Cho-Aza level | Q1 (low)  Q2  Q3 Q4 Q5 (high)  Q1 (low)  Q2  Q3 Q4 Q5 (high) |  | Net survival:  Men  45.4 (43.5-47.4)  46.6 (44.5-48.7)  48.7 (46.5-50.8)  51.2 (48.9-53.4)  53.7 (51.4-56.0)  Women  54.5 (51.5-57.6)  57.9 (54.7-61.2)  60.7 (57.5-64.0)  58.6 (55.1-62.0)  60.2 (56.6-63.9) |  | Net survival:  Men  13.6 (12.2-15.1)  15.5 (13.8-17.1)  16.3 (14.6-18.0)  18.2 (16.4-20.1)  20.8 (18.7-22.8)  Women  25.5 (22.7-28.3)  26.0 (23.0-29.0)  28.4 (25.2-31.5)  29.2 (25.9-32.6)  30.0 (26.5-33.6) | 5-year conditional net survival on being alive at one year (cohort approach, %, 95 % CI):  Men  30.9 (27.9-33.9)  34.2 (30.9-37.5)  34.3 (31.0-37.6)  36.6 (33.3-40.0)  39.8 (36.3-43.3)  Women  47.6 (43.1-52.0)  45.7 (41.0-50.3)  47.4 (42.8-52.0)  50.7 (45.6-55.7)  50.6 (45.4-55.7)  Deprivation gap^2^ (95 % CI):  1-year net survival  Men  **-5.9 (-7.6- -4.2)**  Women **-4.4 (-7.0- -1.7)**  5-year net survival  Men **-4.9 (-6.3- -3.6)**  Women **-4.7 (-7.0- -2.4)**  5-year conditional net survival  Men **-6.7 (-9.3- -4.0)**  Women **-5.8 (-9.7- -1.9)** |
| Kwak 2017a [91]  Korea  Dong | Q4 (most deprived)  Q3  Q2  Q1 (least deprived) |  | Overall survival:  57.0  48.7  50.2  51.4 | Overall survival:  27.9  22.1  28.0  26.4 | Overall survival:  19.2  17.0  21.6  18.6 |  |
| Kwak 2017b [37]  Korea  Dong | Q4 (most deprived)  Q3  Q2  Q1 (least deprived) | 10.4  8.7  10.2  11.8 | Overall survival:  45.3  44.6  46.0  49.4 | Overall survival:  17.7  17.4  18.9  21.1 | KM | 2-year overall survival:  28.9  28.8  30.3  33.3 |
