# Supplementary material for: Socioeconomic Differences and Lung Cancer Survival—Systematic Review and Meta-Analysis
Source: Front Oncol. 2018 Nov 27;8:536. doi: 10.3389/fonc.2018.00536 (PMC6277796; doi:10.3389/fonc.2018.00536)
Supplement: Supplementary file 7 [file Table_7.docx]

**Supplement: Table S7.** Risk of bias assessment for cohort studies according to a modified Newcastle-Ottawa-Scale. Abbreviations: FU = Follow-up; SES=Socioeconomic status; ^a^If the study was population-based, a point was assigned; ^b^If socioeconomic status was assessed by official statistics/registry/census, the study was awarded with one point; ^c^If the study is based on data by a cancer registry, one point was assigned, as only incident cases were registered, other studies were assigned with one point if it was reported that only incident cases were included; ^d^If the study adjusted for at least age one point was assigned. If the analyses were adjusted for additional factors like gender, stage or smoking one additional point was assigned; ^e^If there was independent/blind assessment of outcome (survival), record linkage or authors explained how the outcome was assessed, one point was assigned; ^f^If follow-up was at least as long as the shortest survival rate (for example 3 months) that was reported in article, one point was assigned; ^g^If FU rate ≥90 % or information was ascertained through registration or other administrative offices, one point was assigned; ^h^Information was not given in article but registry is part of EUROCARE or GLOBOCAN; ^i^As stated in Johnson 2014; ^k^As stated in McMillan 2017; ^m^Study is part of The Surveillance, Epidemiology, and End Results Program; ^n^As stated in Ou 2007; ^o^As stated in Tannenbaum 2014

| **SES Level** | **Paper** | **Representative-ness of the exposed cohort^a^** | **Ascertainment of exposure^b^** | **Demonstration that outcome of interest was not present at the start of study^c^** | **Comparability of cohort on the basis of the design or analysis^d^** | **Assessment of outcome^e^** | **Was FU long enough for outcomes to occur^f^** | **Adequacy of FU’s of cohorts^g^** | **Total score** |
| --- | --- | --- | --- | --- | --- | --- | --- | --- | --- |
| **Individual** | Aarts 2013 [44] | 1 | 0 | 1 | 2 | 1 | 1 | 1 | 7 |
|  | Berglund 2010 [45] | 1 | 1 | 1 | 2 | 1 | 1 | 1 | 8 |
|  | Chang 2012 [46] | 1 | 1 | 1 | 2 | 1 | 1 | 1 | 8 |
|  | Chirikos 1984 [30] | 1 | 1 | 1 | 2 | 1 | 1 | 1 | 8 |
|  | Clément-Duchêne 2016 [47] | 1 | 1 | 1 | 2 | 1 | 1 | 1 | 8 |
|  | Dalton 2008 [49] | 1 | 1 | 1 | 2 | 1 | 1 | 1 | 8 |
|  | Dalton 2015 [48] | 1 | 1 | 1 | 2 | 1 | 1 | 1 | 8 |
|  | Di Maio 2012 [50] | 0 | 1 | 0 | 2 | 0 | 1 | 0 | 4 |
|  | Fujino 2007a [51] | 0 | 0 | 0 | 2 | 1 | 1 | 1 | 5 |
|  | Fujino 2007b [32] | 0 | 0 | 0 | 2 | 1 | 1 | 1 | 5 |
|  | Grivaux 2011 [52] | 0 | 0 | 1 | 0 | 1 | 1 | 1 | 4 |
|  | Herndon 2008 [53] | 0 | 0 | 0 | 0 | 0 | 1 | 0 | 1 |
|  | Hussain 2008 [54] | 1 | 1 | 1 | 2 | 1 | 1 | 1 | 8 |
|  | Kravdal 2000 [55] | 1 | 1 | 1 | 2 | 1 | 1 | 1 | 8 |
|  | Pagano 2010 [56] | 1 | 1 | 1 | 2 | 1 | 1 | 1 | 8 |
|  | Pastorino 1990 [57] | 1 | 1 | 1 | 0 | 1^h^ | 1 | 1 | 6 |
|  | Pokhrel 2010 [39] | 1 | 1 | 1 | 2 | 1 | 1 | 1 | 8 |
|  | Skyrud 2016 [58] | 1 | 1 | 1 | 2 | 1^h^ | 1 | 1^h^ | 8 |
|  | Smailyte 2016 [42] | 1 | 1 | 1 | 2 | 1 | 1 | 1 | 8 |
|  | Vågerö 1987 [59] | 1 | 1 | 1 | 1 | 1^h^ | 1 | 1^h^ | 7 |
|  | Yeole 2004 [61] | 1 | 1 | 1 | 2 | 1 | 1 | 1 | 8 |
|  | Yeole 2005 [60] | 1 | 1 | 1 | 2 | 1 | 1 | 1 | 8 |
|  | Yim 2012 [62] | 0 | 1 | 1 | 2 | 1 | 1 | 1 | 7 |
| **Aggregated** | Aarts 2015 [63] | 1 | 1 | 1 | 2 | 1 | 1 | 1 | 8 |
|  | Berglund 2012 [64] | 1 | 1 | 1 | 2 | 1 | 1 | 1 | 8 |
|  | Bonett 1984 [65] | 1 | 1 | 1 | 2 | 1 | 1 | 1 | 8 |
|  | Booth 2010 [66] | 1 | 1 | 1 | 2 | 1^h^ | 1 | 1^h^ | 8 |
|  | Boyd 1999 [67] | 1 | 1 | 1 | 2 | 1 | 1 | 1 | 8 |
|  | Campbell 2000 [29] | 1 | 1 | 1 | 2 | 1^h^ | 1 | 1^h^ | 8 |
|  | Caposole 2014 [68] | 1 | 1 | 1 | 1 | 1 | 1 | 0 | 6 |
|  | Cheyne 2013 [69] | 0 | 1 | 1 | 0 | 1 | 1 | 0 | 4 |
|  | Chouaid 2017 [70] | 1 | 1 | 1 | 2 | 1 | 1 | 0 | 7 |
|  | Coleman 2001 [71] | 1 | 1 | 1 | 1 | 1 | 1 | 1 | 7 |
|  | Coleman 2004 [31] | 1 | 1 | 1 | 1 | 1 | 1 | 1 | 7 |
|  | Currow 2014 [72] | 1 | 1 | 1 | 2 | 1 | 1 | 1 | 8 |
|  | Dabbikeh 2017 [73] | 1 | 1 | 1 | 2 | 1 | 1 | 1 | 8 |
|  | Denton 2017 [74] | 0 | 1 | 1 | 2 | 1 | 1 | 1 | 7 |
|  | Ellis 2014 [75] | 1 | 1 | 1 | 2 | 1 | 1 | 1 | 8 |
|  | Erhunmwunsee 2012 [76] | 1 | 1 | 1 | 0 | 0 | 1 | 1 | 5 |
|  | Evans 2000 [77] | 1 | 1 | 1 | 2 | 1 | 1 | 1 | 8 |
|  | Forrest 2015 [78] | 1 | 1 | 1 | 2 | 1^h^ | 1 | 1^h^ | 8 |
|  | Gomez 2016 [79] | 1 | 1 | 1 | 2 | 1 | 1 | 1 | 8 |
|  | Gorey 1997 [33] | 1 | 1 | 1 | 2 | 1^h^ | 1 | 1^h^ | 8 |
|  | Greenwald 1994 [34] | 1 | 1 | 1 | 2 | 1 | 1 | 1 | 8 |
|  | Greenwald 1998 [80] | 1 | 1 | 1 | 2 | 1 | 1 | 1 | 8 |
|  | Hall 2004 [81] | 1 | 1 | 1 | 2 | 1 | 1 | 1 | 8 |
|  | Hastert 2015 [82] | 1 | 1 | 1 | 2 | 1 | 1 | 1 | 8 |
|  | Haynes 2008 [83] | 1 | 1 | 1 | 2 | 1 | 1 | 1 | 8 |
|  | Hui 2005 [84] | 1 | 1 | 1 | 0 | 1 | 1 | 1 | 6 |
|  | Ito 2014 [85] | 1 | 1 | 1 | 1 | 1 | 1 | 1 | 7 |
|  | Iyen-Omofoman 2011 [86] | 1 | 1 | 1 | 0 | 1 | 1 | 1 | 6 |
|  | Jack 2006 [87] | 1 | 1 | 1 | 2 | 1 | 1 | 1 | 8 |
|  | Jansen 2014 [35] | 1 | 1 | 1 | 2 | 1 | 1 | 1 | 8 |
|  | Jeffreys 2009 [36] | 1 | 1 | 1 | 1 | 1 | 1 | 1 | 7 |
|  | Johnson 2014 [88] | 1 | 1 | 1 | 2 | 1 | 1 | 1 | 8 |
|  | Johnson 2016 [89] | 1 | 1 | 1 | 2 | 1 | 1 | 1^i^ | 8 |
|  | Khullar 2015 [90] | 1 | 1 | 1 | 2 | 1 | 1 | 1^k^ | 8 |
|  | Kwak 2017 a [91] | 1 | 1 | 1 | 2 | 1 | 1 | 1 | 8 |
|  | Kwak 2017b [37] | 1 | 1 | 1 | 2 | 1 | 1 | 1 | 8 |
|  | Lara 2014 [93] | 1 | 1 | 1 | 2 | 1^m^ | 1 | 1^m^ | 8 |
| **Aggregated** | Lara 2017 [92] | 1 | 1 | 1 | 2 | 1^m^ | 1 | 1^m^ | 8 |
|  | Lipworth 1970 [38] | 1 | 1 | 1 | 1 | 0 | 1 | 0 | 5 |
|  | Louwman 2010 [94] | 1 | 1 | 1 | 2 | 1^h^ | 1 | 1^h^ | 8 |
|  | Mackillop 1997 [95] | 1 | 1 | 1 | 2 | 1 | 1 | 1 | 8 |
|  | McMillan 2017 [96] | 1 | 1 | 1 | 2 | 1 | 1 | 1 | 8 |
|  | Melvan 2015 [97] | 1 | 1 | 1 | 0 | 1 | 1 | 1 | 6 |
|  | Niu 2010 [98] | 1 | 1 | 1 | 2 | 1 | 1 | 1 | 8 |
|  | Nur 2015 [99] | 1 | 1 | 1 | 2 | 1 | 1 | 1 | 8 |
|  | O`Dowd 2015 [100] | 1 | 1 | 1 | 0 | 1 | 1 | 1 | 6 |
|  | Ou 2007 [5] | 1 | 1 | 1 | 2 | 1 | 1 | 1 | 8 |
|  | Ou 2008 [101] | 1 | 1 | 1 | 2 | 1^n^ | 1 | 1 | 8 |
|  | Ou 2009 [6] | 1 | 1 | 1 | 2 | 0 | 1 | 1 | 7 |
|  | Pollock 1997 [102] | 1 | 1 | 1 | 2 | 1^h^ | 1 | 1^h^ | 8 |
|  | Rachet 2008 [103] | 1 | 0 | 1 | 1 | 1^h^ | 1 | 1^h^ | 6 |
|  | Rachet 2010 [40] | 1 | 1 | 1 | 2 | 1^h^ | 1 | 1 | 8 |
|  | Riaz 2011 [104] | 1 | 1 | 1 | 1 | 1^h^ | 1 | 1^h^ | 7 |
|  | Rich 2011 [105] | 1 | 1 | 1 | 2 | 1 | 1 | 1 | 8 |
|  | Schrijvers 1995a [106] | 1 | 1 | 1 | 2 | 1 | 1 | 1 | 8 |
|  | Schrijvers 1995b [107] | 1 | 1 | 1 | 2 | 1^h^ | 1 | 1^h^ | 8 |
|  | Shack 2007 [108] | 1 | 1 | 1 | 2 | 1 | 1 | 1 | 8 |
|  | Shugarman 2008 [109] | 1 | 1 | 1 | 2 | 1^m^ | 0 | 1^m^ | 7 |
|  | Sloggett 2007 [41] | 1 | 1 | 1 | 2 | 1 | 1 | 1 | 8 |
|  | Stanbury 2016 [110] | 1 | 1 | 1 | 2 | 1 | 1 | 1 | 8 |
|  | Sutherland 2008 [111] | 1 | 1 | 1 | 0 | 0 | 1 | 0 | 4 |
|  | Tannenbaum 2014 [112] | 1 | 1 | 1 | 2 | 1 | 1 | 1 | 8 |
|  | Tervonen 2017 [22] | 1 | 1 | 1 | 2 | 1 | 1 | 1 | 8 |
|  | Vercelli 2006 [113] | 1 | 1 | 1 | 1 | 1^h^ | 1 | 1^h^ | 7 |
|  | Wang 2017a [114] | 1 | 1 | 1 | 2 | 1^m^ | 1 | 1^m^ | 8 |
|  | Wang 2017b [115] | 1 | 1 | 1 | 2 | 1^m^ | 1 | 1^m^ | 8 |
|  | Wen 2005 [116] | 1 | 1 | 1 | 0 | 1 | 1 | 1 | 6 |
|  | Yang 2010 [117] | 1 | 1 | 1 | 2 | 1^o^ | 1 | 1^o^ | 8 |
|  | Yu 2008 [119] | 1 | 1 | 1 | 2 | 1 | 1 | 1 | 8 |
|  | Yu 2014 [118] | 1 | 1 | 1 | 1 | 1^m^ | 1 | 1^m^ | 7 |
|  | Zhang-Salomons 2006 [43] | 1 | 1 | 1 | 2 | 1^h^ | 1 | 1^h^ | 8 |
