# Supplementary material for: Socioeconomic Differences and Lung Cancer Survival—Systematic Review and Meta-Analysis
Source: Front Oncol. 2018 Nov 27;8:536. doi: 10.3389/fonc.2018.00536 (PMC6277796; doi:10.3389/fonc.2018.00536)
Supplement: Supplementary file 8 [file Data_Sheet_1.DOCX]

Supplementary Material

**Socioeconomic Differences and Lung Cancer Survival – Systematic Review and Meta-Analysis**

Isabelle Finke, Gundula Behrens, Linda Weißer, Hermann Brenner, Lina Jansen*

*** Correspondence:** Dr. Lina Jansen, [l.jansen@dkfz.de](mailto:l.jansen@dkfz.de), Tel: +49 6221 42-1357
Division of Clinical Epidemiology and Aging Research, German Cancer Research Center (DKFZ), Im Neuenheimer Feld 581, 69120 Heidelberg, Germany

**CODING MANUAL MODIFIED NEWCASTLE-OTTAWA QUALITY ASSESSMENT SCALE**

**COHORT STUDIES**

Note: A study can be awarded a maximum of one star for each numbered item within the Selection and Outcome categories. A maximum of two stars can be given for Comparability.

**SELECTION**

1. Representativeness of the cohort
   1. Truly representative of the average residents in the community*

*Defined catchment area/geographic boundaries, whole area (complete enumeration) or random sample/census*

- 1. Somewhat representative of the average residents in the community*

*Membership in health maintenance organizations*

- 1. Selected group of users e.g. nurses, volunteers
  2. No description of the derivation of the cohort

1. Ascertainment of exposure (Socioeconomic status or Index measure)
   1. Secure record*

*E.g. surgical records, registry data, data from national/official statistics (aggregated or individual data from census)*

- 1. Structured interview*
  2. Written self-report (questionnaire)
  3. No description

1. Demonstration that outcome of interest was not present at start of study
   1. Yes*

*In the case of mortality/survival studies, outcome of interest is still the presence of a disease/incident, rather than death. That is to say that a statement of no history of disease or incident earns a star.*

*Cancer registries always get a “yes” because they only register incident cases.*

- 1. No

**COMPARABITLITY**

1. Comparability of cohorts on the basis of the design or analysis

*A maximum of 2 stars can be allotted in this category. Both exposed and non-exposed individuals must be matched in the design and/or confounders must be adjusted for in the analysis. Statements of no differences between groups or that differences were not statistically significant are not sufficient for establishing comparability. Note: If the relative risk (or hazard ratio, HR) for the exposure of interest is adjusted for the confounders listed, then the groups will be considered to be comparable on each variable used in the adjustment. Stratification of results e.g. by age groups or gender is also considered as adjustment.*

- 1. Study controls for age*
  2. Study controls for any additional factor (for example: sex/gender, smoking, stage, treatment)*

**OUTCOME**

1. Assessment of outcome
   1. Independent blind assessment*

*Independent or blind assessment stated in the paper, or confirmation of the outcome by reference to secure records (medical records etc.)*

- 1. Record linkage*

*E.g. identified through ICD codes on database records (= registry data), death certificates*

- 1. Self-report
  2. No description

1. Was follow-up long enough for outcomes to occur
   1. Yes (adequate follow-up period: long enough to reach study aim, for example, if study reported 3 month survival rates, follow-up has to be at least 3 months)*
   2. No
2. Adequacy of follow up of cohorts

*This item assesses the follow-up of the cohort to ensure that losses are not related to either the exposure or the outcome.*

- 1. Complete follow up – all subjects accounted for*

*E.g. Information was ascertained through registration of other administrative offices*

- 1. Subjects lost to follow up unlikely to introduce bias – small number lost -> 90% follow up, or description provided of those lost*
  2. Follow up rate < 90 % and no description of those lost
  3. No statement
