# Supplementary material for: Socioeconomic Differences and Lung Cancer Survival—Systematic Review and Meta-Analysis
Source: Front Oncol. 2018 Nov 27;8:536. doi: 10.3389/fonc.2018.00536 (PMC6277796; doi:10.3389/fonc.2018.00536)
Supplement: Supplementary file 9 [file Image_1.PDF]

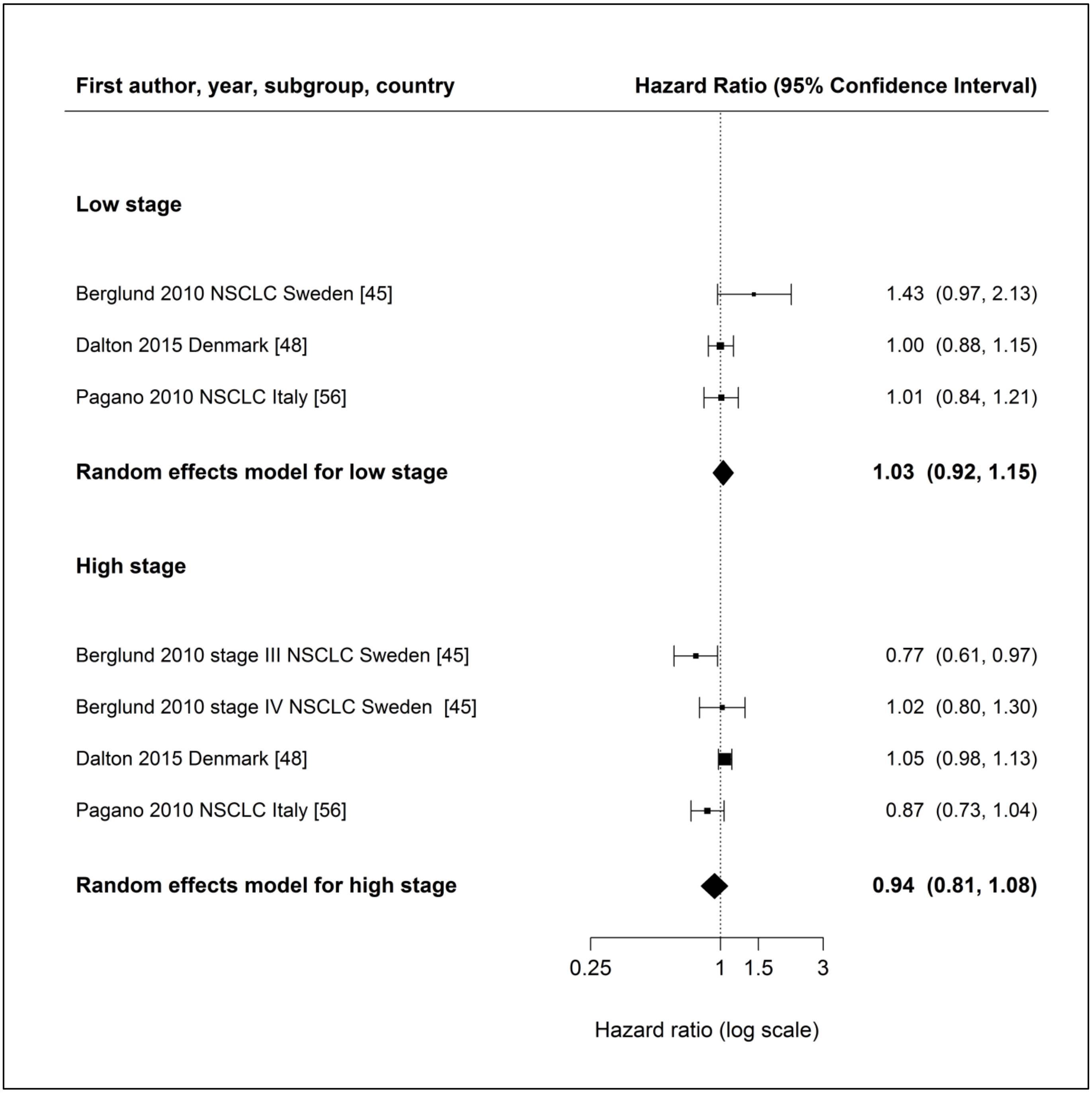

Supplement: Figure S1. Meta-analyses of the association between individual education (reference: high education) and lung cancer survival stratified by stage (low stage = stage I/II (TNM version 5/6) and high stage = stage III/IV)
