# Supplementary material for: Socioeconomic Differences and Lung Cancer Survival—Systematic Review and Meta-Analysis
Source: Front Oncol. 2018 Nov 27;8:536. doi: 10.3389/fonc.2018.00536 (PMC6277796; doi:10.3389/fonc.2018.00536)
Supplement: Supplementary file 13 [file Image_5.PDF]

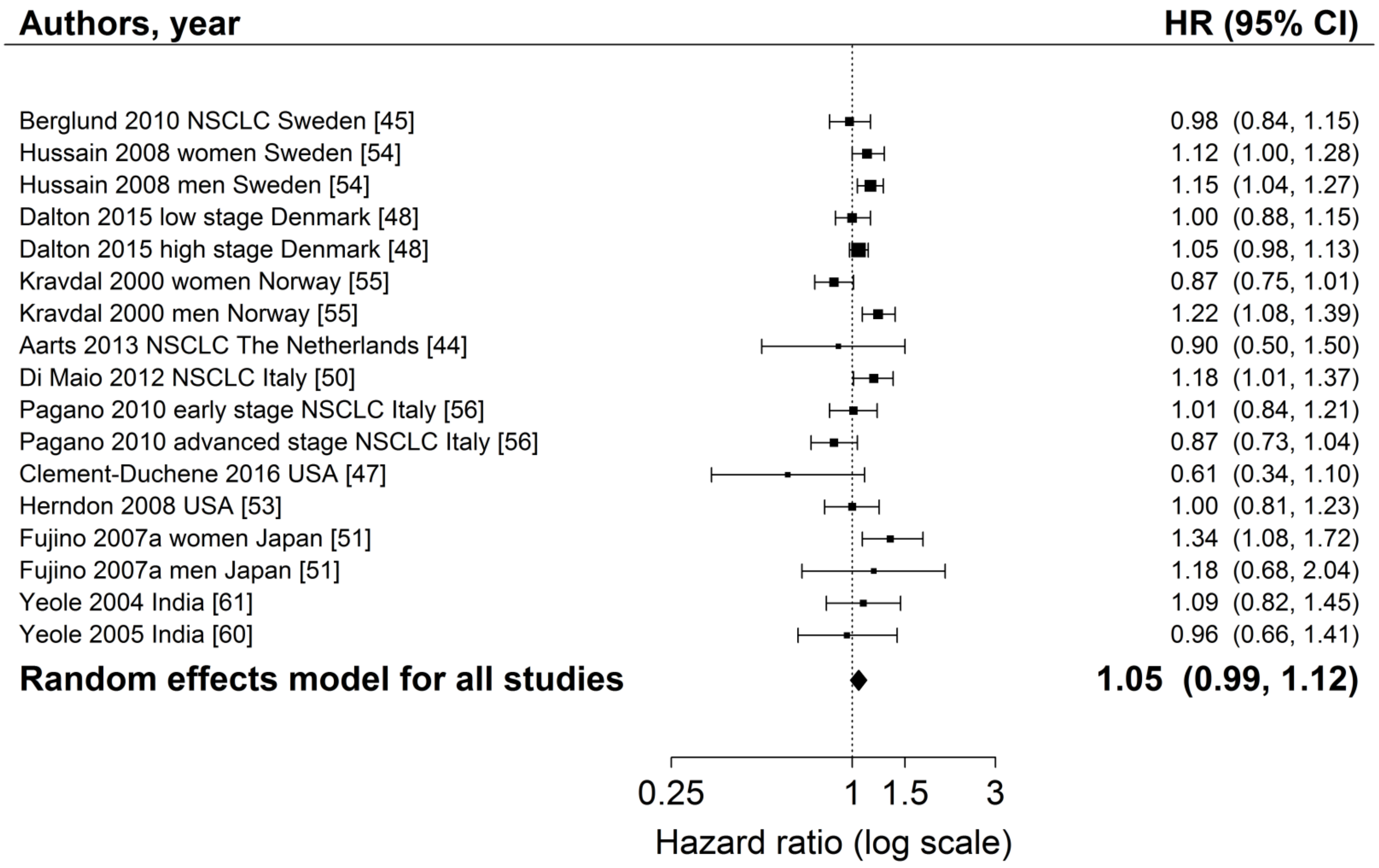

Figure S5. Sensitivity analysis: Meta-analysis of the association between individual education (reference: high education) and lung cancer survival, including three studies excluded in main analysis because of low quality score. NSCLC = non-small cell lung cancer. Kravdal 2000: highest educational group, men = 17+ years, women = 13-17+ years.
