# Supplementary material for: Socioeconomic Differences and Lung Cancer Survival—Systematic Review and Meta-Analysis
Source: Front Oncol. 2018 Nov 27;8:536. doi: 10.3389/fonc.2018.00536 (PMC6277796; doi:10.3389/fonc.2018.00536)
Supplement: Supplementary file 15 [file Image_7.PDF]

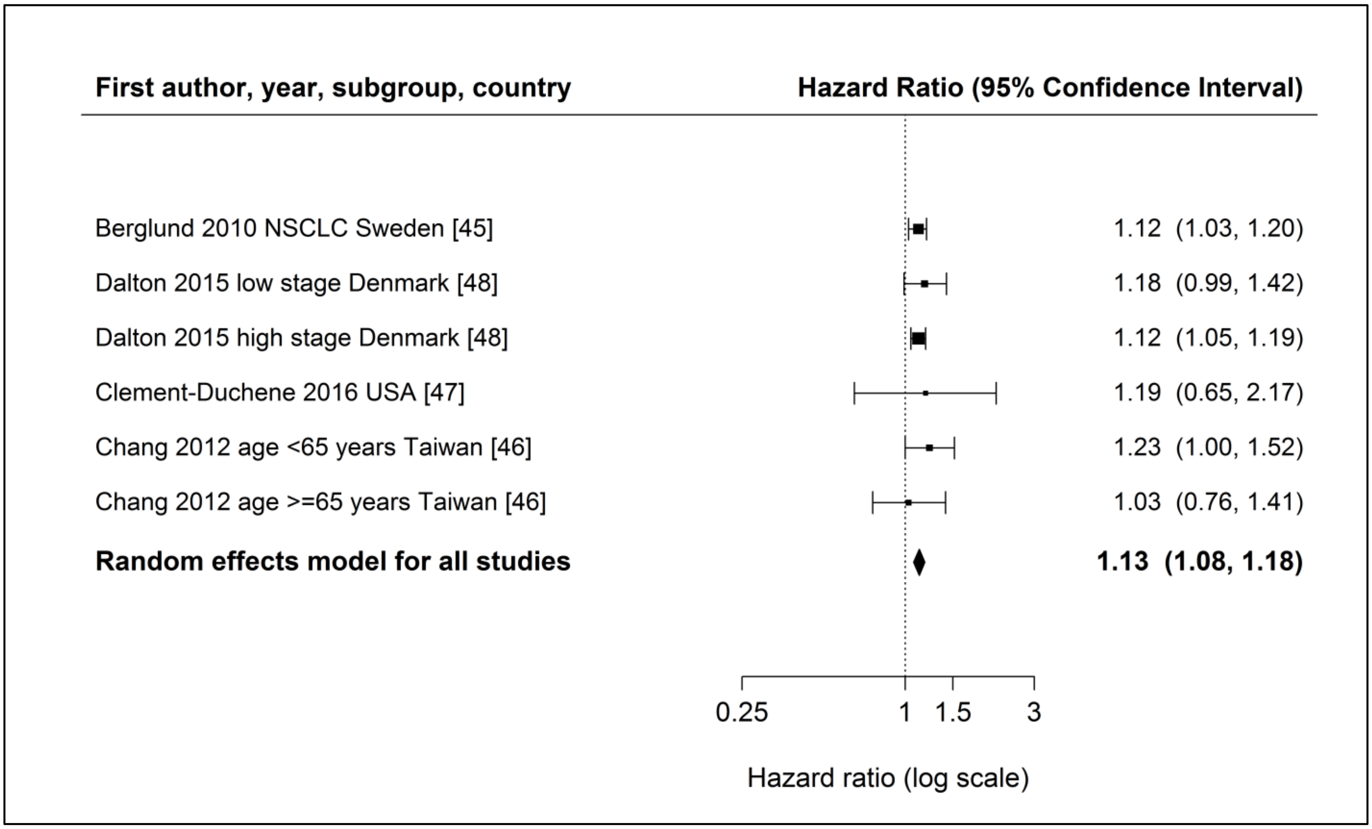

Figure S7. Meta-analysis of the association between individual income (reference: high income) and lung cancer survival including studies with treatment adjustment, order by region: Europe, USA, Asia. NSCLC = non-small cell lung cancer. Clement-Duchene 2016 included non-smokers only in their analysis.
