# Supplementary material for: Socioeconomic Differences and Lung Cancer Survival—Systematic Review and Meta-Analysis
Source: Front Oncol. 2018 Nov 27;8:536. doi: 10.3389/fonc.2018.00536 (PMC6277796; doi:10.3389/fonc.2018.00536)
Supplement: Supplementary file 16 [file Image_8.PDF]

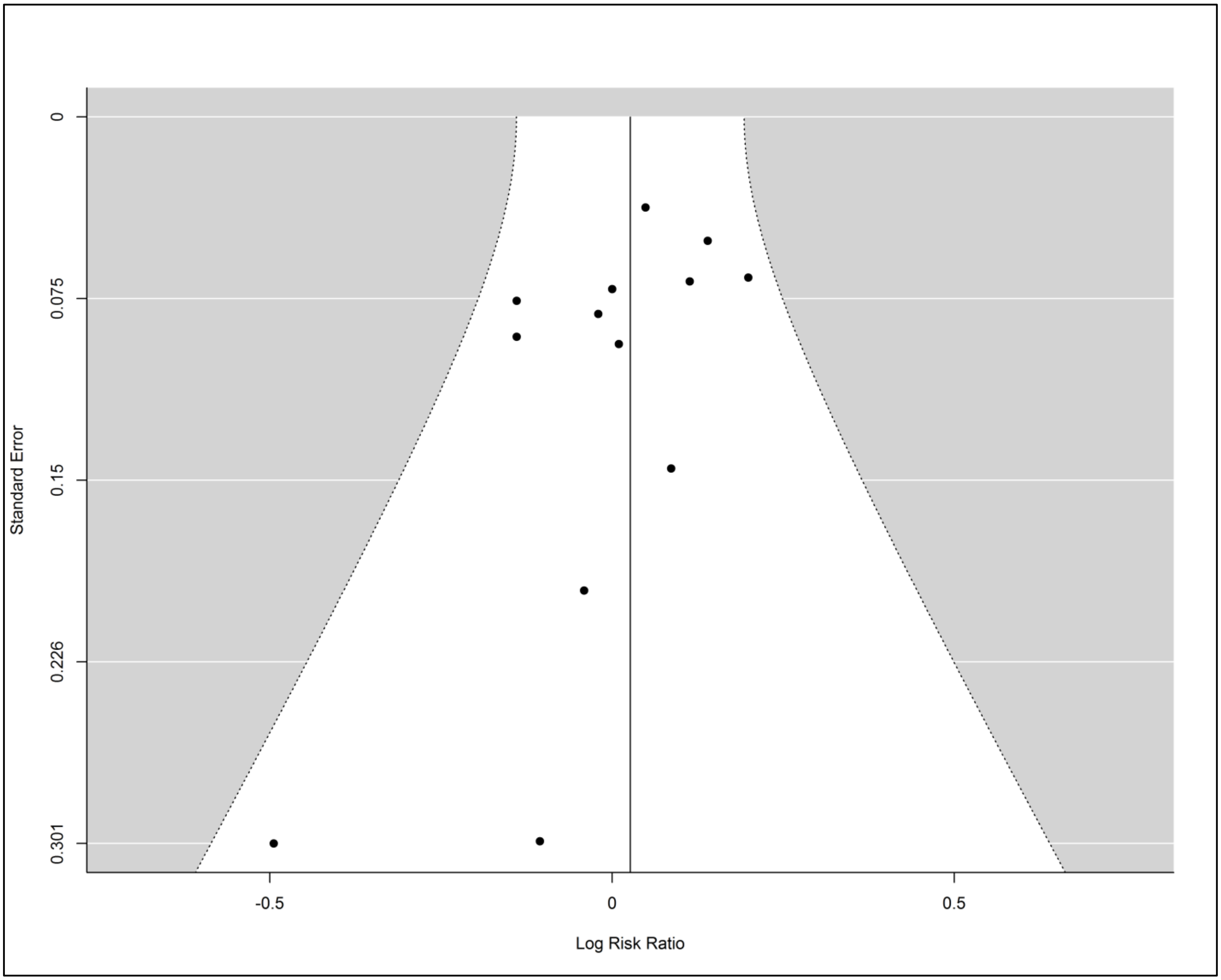

Figure S8. Funnel plot of the meta-analysis on individual education and survival after lung cancer. Begg's test: p = 0.13, Egger's test: p = 0.07.
