# Supplementary material for: Socioeconomic Differences and Lung Cancer Survival—Systematic Review and Meta-Analysis
Source: Front Oncol. 2018 Nov 27;8:536. doi: 10.3389/fonc.2018.00536 (PMC6277796; doi:10.3389/fonc.2018.00536)
Supplement: Supplementary file 17 [file Image_9.pdf]

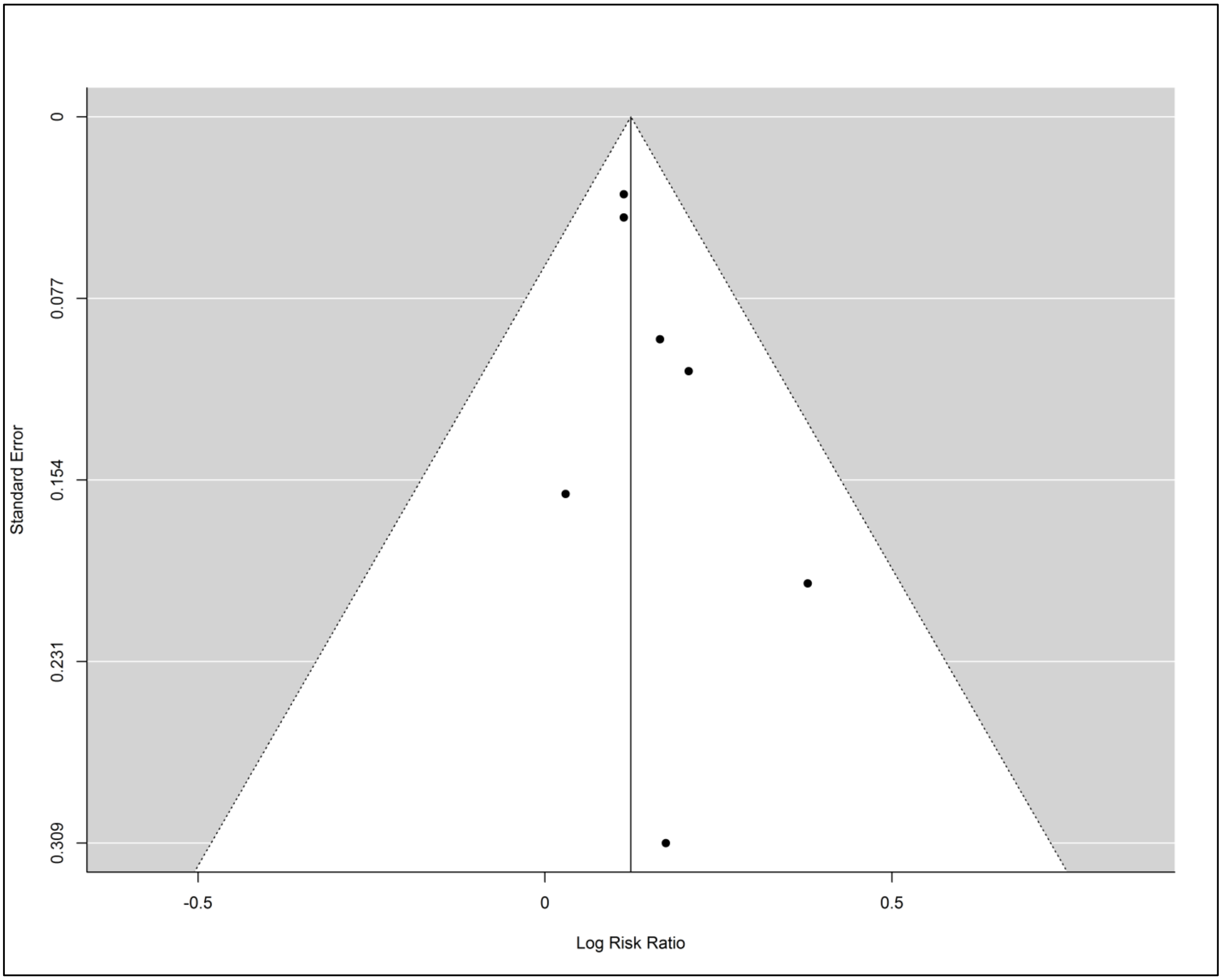

Figure S9. Funnel plot of the meta-analysis on individual income and survival after lung cancer. Begg’s test: p = 0.38, Egger’s test: p = 0.34.
